# The correlation between the MYBL2/CDCA8 signaling pathway of malignant melanoma

**DOI:** 10.1016/j.heliyon.2024.e32485

**Published:** 2024-06-05

**Authors:** Chen Wu, Jiahui Jiang, Chao Ci

**Affiliations:** Department of Dermatology, The First Affiliated Hospital of Wannan Medical College, No. 2 Zheshan West Road, Wuhu, Anhui, 241001, China

**Keywords:** Malignant melanoma, MYB proto-oncogene like 2, Cell division cycle associated 8

## Abstract

**Objective:**

Investigating the effects of MYB proto-oncogene like 2 (MYBL2)-mediated regulation of Cell division cycle associated 8 (CDCA8) expression on the biological activity of cutaneous malignant melanoma cells.

**Methods:**

A375 cells with MYBL2 and CDCA8 overexpression and knockdown were evaluated using migration, invasion, and proliferation assays. Besides, cell apoptosis was quantified by flow cytometry. To investigate the tumorigenic effects of MYBL2 knockdown *in vivo*, A375 cells with MYBL2 knockdown were injected in BALB/C nude mice.

**Results:**

The levels of MYBL2 and CDCA8 gene expression were notably elevated in A375 cells in comparison to HaCat cells (P < 0.05). Downregulation of MYBL2 led to a notable reduction in the migratory and invasive capability of A375 cells in vitro (P < 0.001). On the contrary, overexpression of MYBL2 enhanced migration and invasion ability (P < 0.001). There existed a positive correlation between CDCA8 and MYBL2 gene and protein expression levels after overexpression or knockdown of MYBL2 (P < 0.001). In the *in vivo* tumorigenic study, the MYBL2 knockdown group displayed a substantial decrease in tumor volume (P < 0.01) and exhibited decreased CDCA8 expression in tumors in comparison to the control group.

**Conclusion:**

We arrived at such a conclusion that MYBL2 promoted the migration, invasion and proliferation ability of cutaneous malignant melanoma cells by targeted regulation of CDCA8 expression in this study.

## 1 Introduction

Malignant melanoma (MM) represents the most formidable manifestation of skin cancer in humans and is characterized by rapid progression, early metastasis, high likelihood of recurrence, and unfavorable prognosis. The pathogenesis of melanoma involves a combination of factors, such as genetic predisposition, exposure to ultraviolet radiation, and immune dysregulation within the local tumor microenvironment [1]. Currently, the primary treatment for MM involves extensive surgical resection, chemotherapy, immunotherapy and so on [2]. Surgical resection is typically used for the management of early-stage melanoma and generally yields favorable outcomes. However, the therapeutic alternatives for advanced-stage MM are limited, resulting in a poor prognosis. If radical surgical resection is carried out following early diagnosis, the survival rate over a five-year period for individuals with MM can reach 91 % [3]. Another study reported that the rate of survival over a five-year period could reach 52 % for patients who were diagnosed with advanced metastatic MM with a mix of immune therapy medications [4]. Therefore, elucidating the fundamental mechanisms of melanoma pathogenesis is critical to facilitating the development of targeted therapeutic agents and improving patient prognosis.

Cell division cycle associated 8 (CDCA8), is an important regulator of cell division and is crucial for the physiological process of mitosis. Numerous investigations had demonstrated that CDCA8 was implicated in the pathogenesis of diverse malignancies. Jiao DC et al. [5] reported high expression of CDCA8 in breast cancer tissues and its negative correlation with overall survival. Lee S et al. [6] analyzed the gene expression profiles of 556 skin melanoma patients from three databases (TCGA, GSE19243, and GSE22153) and found that patients exhibiting elevated expression levels of CDCA8 demonstrated a poorer prognosis. These studies indicated that CDCA8 maybe regarded as a molecular marker for malignant tumors [[7], [8], [9]]. We previously reported high CDCA8 mRNA and protein levels in skin melanoma tissues. We also discovered that high CDCA8 expression was related to increased migration, invasion and proliferation of skin melanoma cell lines. Mechanistically, CDCA8 upregulated the expression of ROCK1 and phosphorylated myosin light chain, two key proteins involved in the ROCK signaling pathway [10].

Despite previous indications from our research suggesting the potential involvement of CDCA8 overexpression in melanoma pathogenesis, the regulatory mechanisms governing CDCA8 remain elusive. Subsequent exploration of the Cistrome and JASPAR databases revealed MYB proto-oncogene like 2 (MYBL2) is a crucial transcriptional regulator of CDCA8. MYBL2 plays a significant role in cell viability, cell cycle progression, and cellular differentiation, implying that malfunction of MYBL2 may contribute to the progression and initiation in tumor [11,12]. Numerous studies had linked MYBL2 gene overexpression to the development of various malignancies. Nakajima et al. [13] found MYBL2 gene amplification in 36 out of 66 primary liver cancer patients. Qin et al. [14] conducted whole-genome and whole-exome sequencing on paired samples from 10 esophageal cancer patients and found MYBL2 gene amplification in 57 esophageal cancer tissue samples. Qi et al. [15] demonstrated that MYBL2 regulates CDCA8 and was implicated in the migration, invasion and proliferation of breast cancer cells. MYBL2 knockout markedly reduced CDCA8 mRNA expression and protein secretion in breast cancer tissues, while MYBL2 overexpression significantly upregulated CDCA8 gene expression and protein level. Vera et al. [16] reported the increased MYBL2 gene expression in melanoma cell lines; however, its role in melanoma pathogenesis remains elusive.

From the aforementioned studies, MYBL2 and CDCA8 may have significant roles on the initiation and progression of melanoma. Herein, we hypothesized that MYBL2 may modulate CDCA8 expression and contribute to the development of cutaneous melanoma. Our study findings may serve as a foundation for future molecular targeting approaches for melanoma treatment.

## 2 Methods

### 2.1 Cell lines and cell culture

The human immortalized keratinocyte cell line HaCat (Saibai Kang, iCell-h066) and human MM cell line A375 (Saibai Kang, iCell-h007) were cultured in DMEM with the addition of fetal bovine serum (FBS) at a concentration of 10 %, and kept at 37 °C with 5 % CO_2_.

### 2.2 RT-qPCR

The primer sequences for MYBL2, CDCA8 and GAPDH are provided in Table 1. The 2^-ΔΔCt^ approach was used to assess the relative gene expression levels, which were subsequently normalized against GAPDH expression. Each sample underwent triplicate analysis.

**Table 1:** Primer sequence.

Primer	Sequence (5′-3′)
MYBL2-F	TCTGGACGCAGTGCGAACACCA
MYBL2-R	CCCACTTGTAAGGCAGGCTCGTTT
CDCA8-F	ATGAACTGGCTTGACTACTTCGC
CDCA8-R	CCTGTATTACCTTTCGTGTTTTGG
GAPDH-F	AATCCCATCACCATCTTCCA
GAPDH-R	AAATGAGCCCCAGCCTTCT

### 2.3 siRNAs and plasmids

siRNAs targeting MYBL2 (Gene ID: 4605) and CDCA8 (Gene ID: 55143) genes were obtained from General Biosynthesis. The siRNA sequences are presented in Table 2.

**Table 2:** siRNA Sequence.

	sense (5′-3′)	antisense (5′-3′)
MYBL2 (human) siRNA- 799	CCAUAAAGGAGGAGGAAAATT	UUUUCCUCCUCCUUUAUGGTT
MYBL2 (human) siRNA- 1792	CCGAGAAGCAGAAGAGGAATT	UUCCUCUUCUGCUUCUCGGTT
MYBL2 (human) siRNA- 358	CCAAAGAGGAAGACCAAAATT	UUUUGGUCUUCCUCUUUGGTT
CDCA8 (human) siRNA- 546	AGGAAAAGGAAAAGGGAAATT	UUUCCCUUUUCCUUUUCCUTT
CDCA8 (human) siRNA- 174	GGAAAUACGAAUCAAGCAATT	UUGCUUGAUUCGUAUUUCCTT
CDCA8 (human) siRNA- 437	AUGAAAUGAUAGUGGAAGATT	UCUUCCACUAUCAUUUCAUTT
Negative control	UUCUCCGAACGUGUCACGUTT	ACGUGACACGUUCGGAGAATT

### 2.4 Cell transfection

Cell transfection was carried out using Lipofectamine 2000 when cell confluence reached 60 %. Briefly, 5 μL of siRNA was diluted in 100 μL of Opti-MEM and gently mixed by pipetting three times. Separately, 2 μL of Lipofectamine 2000 was diluted in 100 μL of Opti-MEM and gently mixed by pipetting three times. The transfection reagent was mixed with the siRNA diluent by pipetting three times and incubated 20 min at room temperature. The resultant transfection complex was delivered to the cellular environment, followed by addition of fresh complete culture medium and gentle agitation. Cells were harvested and analyzed after culturing for 48 h.

### 2.5 Western blot

The cells were gathered and ruptured in 500 μL of RIPA solution with 1 mM PMSF on ice for half an hour. The supernatant was harvested after centrifuging, and protein content was quantified employing a BCA protein analysis kit. Equitable quantities of protein were combined with 5 × Denaturing Loading Buffer, heated in water for 5 min, and cooled on ice for another 5 min. Specimens (20 μg) were segregated by SDS-PAGE at 80V for 30 min and subsequently at 120V for 2 h. The gel was moved onto a PVDF membrane in chilled water using the damp transfer technique at a constant electrical flow of 300 mA for 1 h. Prior to transfer, the PVDF membrane was activated by soaking in methanol for 30 s. Following transfer, the PVDF membrane is subjected to a single TBST wash. Subsequently, it is immersed in a sealing liquid, gently agitated for 2 h, and subjected to another TBST wash. The membrane was subjected to incubation with the primary antibodies specifically directed towards CDCA8 (1:1000), MYBL2 (1:1000), Beta Tubulin (1:2000), and GAPDH (1:2000). The membrane was rinsed in TBST three times, each for 10 min, then exposed to secondary antibodies including anti-mouse IgG-HRP (1:2000) and anti-rabbit IgG-HRP (1:2000) at room temperature for 1 h. After additional cleansing with TBST on five occasions, lasting 10 min for each cycle, ECL detection reagent was added for band detection and imaging.

### 2.6 Cell proliferation assay

A375 cells were cultured in a 96-well plate at the density of 10000 cells per well with six replicate wells for each group, and cultured for 24 h. Subsequently, 10 μL of CCK8 solution was added to each well, followed by an incubation period of 1 h. The absorbance values at 450 nm was assessed utilizing an ELISA plate reader.

### 2.7 Cell migration assay

Cells were harvested and reconstituted in serum-depleted medium at a density of 2 × 10^5^ cells/mL. Subsequently, 600 μL of 10 % FBS DMEM was dispensed into each compartment of a 24-well plate, and a transwell PET membrane insert with an 8 μm pore size from Falcon was placed inside. Following that, 200 μL of the cellular mixture was included into the upper partition of the insertion device. The cells were incubated for 48 h. The transwell insert was removed and the top chamber was discarded. The cells adherent to the lower surface of the barrier were immobilized with 4 % paraformaldehyde for a duration of 15 min, subsequently subjected to staining with a 0.1 % solution of crystal violet for 20 min. Enumeration of the stained cells ensued, and photomicrographs were captured utilizing an inverted microscope.

### 2.8 Cell invasion assay

The transwell invasion assay followed the same procedure as the migration assay, with the only difference being that the top surface of the Transwell chamber membrane was coated with Matrigel matrix. Matrigel matrix was diluted 1:8 with serum-free medium, and 80 μL of the matrix gel solution was applied on the upper surface of the PET membrane evenly. After 1 h incubation in a cell incubator, the matrix gel solution was removed, and the cell suspension was added. The subsequent steps were identical to those of the migration assay.

### 2.9 Scratch assay

Cells were grown in a six-well plate for one day. A linear incision was created in the cellular stratum utilizing a pipette tip. Subsequent to two washes with PBS, the medium was transitioned to a serum-deprived medium. Photographs were obtained at 0 h, 24 h and 48 h.

### 2.10 Cell apoptosis detection

Cells (including cells in the supernatant) were harvested by centrifugation, then washed with pre-chilled PBS, and 1 × 10^5^ cells were collected. Subsequently, resuspending cells with 500 μL of 1 × Binding Buffer and adding 5 μL of Annexin V-FITC and 10 μL of PI to each tube. Following gentle vortexing, the cells were incubated under dark conditions at room temperature for 5 min. On the flow cytometer, Annexin V-FITC fluorescence was detected via the FITC emission channel (Excitation wavelength = 488 nm, Emission wavelength = 530 nm), while PI fluorescence was detected via the PI emission channel (Excitation wavelength = 535 nm, Emission wavelength = 615 nm).

### 2.11 Construction of MYBL2 shRNA plasmid

Two shRNA sequences targeting the MYBL2 gene were designed using the online software from Thermo official website and the NCBI human MYBL2 gene sequence (NM_001278610.2). The sequences were as follows: MYBL2-SH1 (1063): ACCGGTGGTGTGACCTGAGTAAATTTGTTCAAGAGACAAATTTACTCAGGTCACACCTTTTTGGATCC; and MYBL2-SH2 (1418): ACCGGTGCCCAAGAGCACACCTGTTAATTCAAGAGATTAACAGGTGTGCTCTTGGGCTTTTTGGATCC. The shRNA sequences were synthesized and inserted into the pHHsi vector by Anhui General Biotechnology Company; the vectors were confirmed by sequencing. The pHHsi vector contains EGFP and puromycin N-acetyltransferase genes for selection. Large-scale plasmid extraction was performed to obtain plasmids with high concentration and no residual endotoxins for subsequent co-transfection experiments.

### 2.12 Construction of the MYBL2 gene knockdown cell line

We seeded 293T cells into a 10-cm culture dish and cultured cells for 24 h. Two EP tubes were prepared, labeled as A and B, and 500 μL of physiological saline solution was added to each. PEI (60 μg) was added to tube A and vortexed thoroughly, while tube B contained shRNA plasmid and two helper plasmids, pSPAX2 and pMD2.G, at a ratio of 4:3:1, totaling 24 μg. The contents of tube A were added to tube B; the sample was gently mixed and incubated for 20 min. The solution was introduced to the cells and left to incubate overnight. The medium was then switched to 5 % FBS DMEM, and the cells underwent extended culture for an additional 48 h. The supernatant was harvested and preserved at 4 °C. New liquid was added to the cells, and they were continued to be grown for another 24 h. The supernatant was collected again, and the two collections were mixed. The supernatants underwent centrifugation at 12,000 rpm for 10 min to separate, followed by the elimination of cellular debris. The resulting combination was then filtered through a 0.45 μm membrane. The PEG8000 method was used to concentrate the virus. For every 30 mL of filtered virus stock, 7.5 mL of a 5X PEG-8000-NaCl solution was added. The mixture was agitated every 30 min five times and then placed at 4 °C for one night. The supernatant was separated via centrifugation, and the tube was left undisturbed for 2 min to allow any residual liquid to settle. An appropriate amount of a slow virus dissolution solution was added to dissolve the slow virus precipitate. A375 cells were placed in a 12-well container, with 1 × 10^5^ cells seeded in each well. The viruses were added (two interference groups and one control), with a final concentration of 8 μg/mL polybrene. After a 24-h period, the medium was exchanged, and the cells underwent extended culture for an additional 24 h. Then the cells were passaged and cultured in different concentrations of puromycin (2 μg/mL, 3 μg/mL, 5 μg/mL, and 6 μg/mL). The medium was refreshed every three days, and puromycin was added at the required concentrations. Cell viability and fluorescence were monitored. Cells showing normal growth were selected for further expansion using a puromycin concentration of 5 μg/mL. After the cells were expanded, they were analyzed by qPCR to confirm gene silencing. Cells showing effective gene silencing and control cells (A375-NC group and A375-si-MYBL2 cells) were expanded and cryopreserved.

### 2.13 Animal experiments

Fourteen male BALB/C nude mice, aged four weeks, were allowed to acclimate for one week prior to experiments. Cells were suspended in PBS (A375-NC group and A375-si-MYBL2, 1 × 10^7^ cells per sample) and inoculated into the mice (n = 7/group). The cells were administered unilaterally into the axillary region, with a dosage of 100 μL per mouse. Tumor formation in the animals was closely monitored. The maximum diameter (D) and minimum diameter (d) of the tumors were assessed every three days utilizing a caliper for measurement, and the body weight of mice was recorded. At last of the observation period, the mice were euthanized via cervical dislocation. Subsequent to this, the tumor tissues were excised, weighed and photographed.

### 2.14 Statistical analysis

The results were expressed as mean ± standard deviation, and significance was defined as p < 0.05. Statistical analyses were conducted using IBM SPSS software version 19.0. The independent sample *t*-test was employed for comparing the data from two groups, while one-way analysis of variance (ANOVA) was utilized for comparing data from multiple groups.

## 3 Results

### 3.1 MYBL2 and CDCA8 gene expression in HaCat and A375 cells

**Fig. 1:**
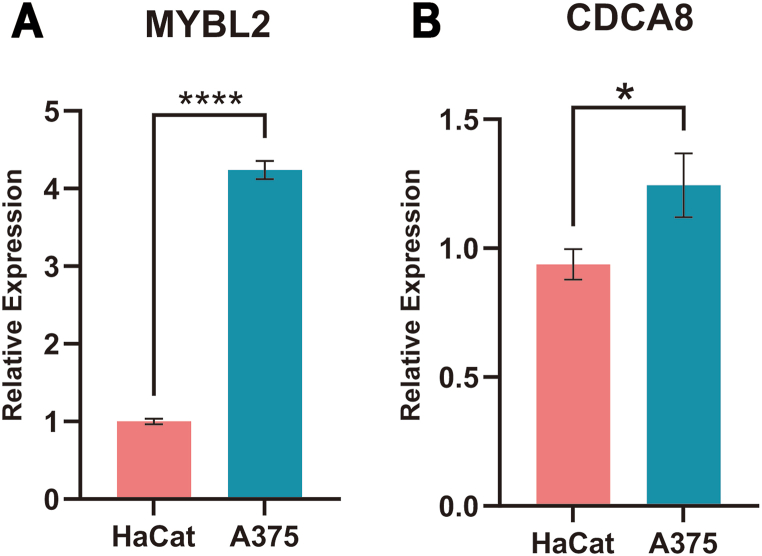
MYBL2 (A) and CDCA8 (B) mRNA expression in HaCat and A375 cells. (n = 3). *P < 0.05, ****P < 0.0001.

RT-qPCR analysis revealed that A375 cells exhibited significantly higher mRNA expression levels of MYBL2 (P = 0.000) (Fig. 1A) and CDCA8 (P = 0.018) (Fig. 1B) compared with HaCat cells.

### 3.2 siRNA-mediated silencing of MYBL2 and CDCA8

**Fig. 2:**
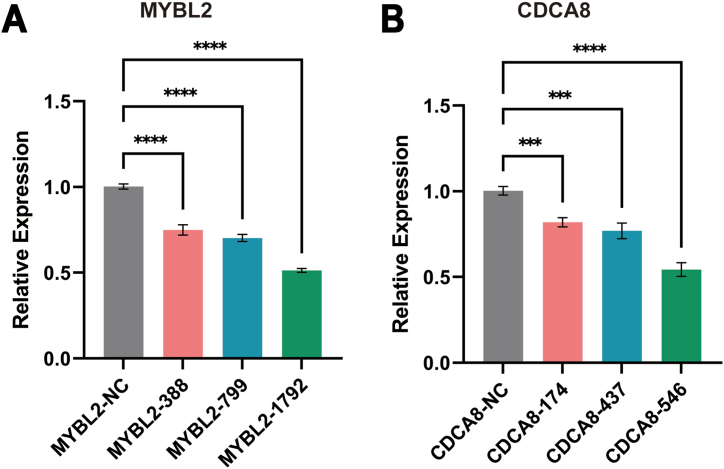
Silencing efficiency of different siRNAs targeting MYBL2 (A) and CDCA8 (B) in A375 cells. (n = 3). ***P < 0.001, ****P < 0.0001.

Three different siRNAs were designed for MYBL2 and CDCA8 and transfected into A375 cells. RT-qPCR revealed that among the three siRNAs tested for each gene, MYBL2-1792 (Fig. 2A) and CDCA8-546 (Fig. 2B) exhibited the highest silencing efficiency. We selected these siRNAs for subsequent experiments.

### 3.3 Overexpression and inhibition of MYBL2 and CDCA8

**Fig. 3:**
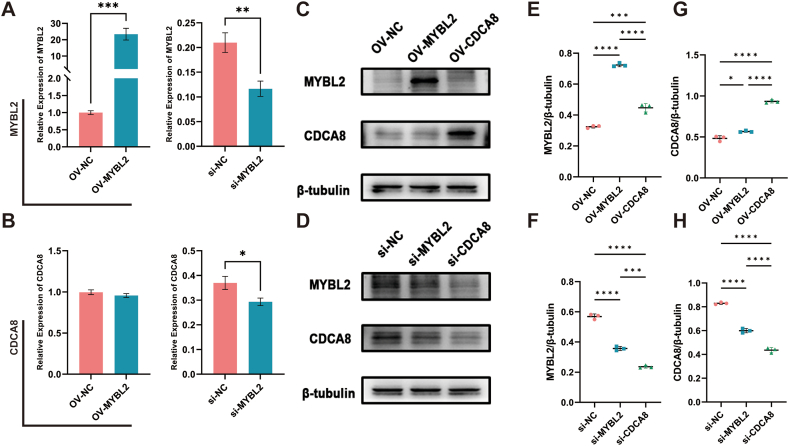
Overexpression and inhibition of MYBL2 and CDCA8 in A375 cells. mRNA expression levels of MYBL2 and CDCA8 after overexpression and silencing MYBL2 (A–B). Protein expression of MYBL2 and CDCA8 after overexpression and inhibition of both MYBL2 and CDCA8 (C–D) (The raw images were provided as Fig. S1). Statistical analysis of results from C and D, presented as MYBL2/β-tubulin and CDCA8/β-tubulin (n = 3) (*E*–G). *P < 0.05, **P < 0.01, ***P < 0.001, ****P < 0.0001.

We next conducted overexpression and inhibition experiments targeting MYBL2 and CDCA8 in A375 cells. Silencing of MYBL2 led to a notable decrease of MYBL2 mRNA expression (P < 0.01) and CDCA8 gene expression (P < 0.05) (Fig. 3A and B). Analysis of protein expression levels revealed a positive correlation between MYBL2 and CDCA8 expression (Fig. 3C and D). Notably, although the overexpression of MYBL2 resulted in an elevation of CDCA8 protein expression, the CDCA8 mRNA expression level did not show any notable changes. Furthermore, a positive relationship was noted between the expression of MYBL2 and the regulation of CDCA8. (Fig. 3E–H), suggesting the possible presence of a bidirectional regulatory mechanism between MYBL2 and CDCA8.

### 3.4 Cell viability assay of A375 cells after MYBL2 silencing or overexpression

**Fig. 4:**
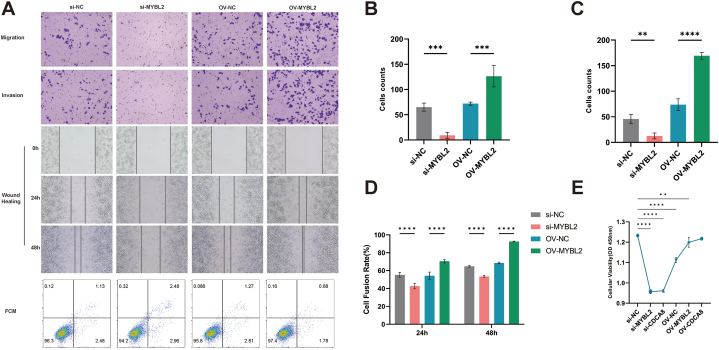
A375 cell activity after MYBL2 overexpression and silencing. Evaluation of A375 cell activity after MYBL2 overexpression and silencing by transwell assay, scratch assay, and flow cytometry (A) (The raw images of migration ability and invasion ability of A375 cells after MYBL2 overexpression and silencing were provided as Fig. S2. The raw images of scratch assay and flow cytometry of A375 cell after MYBL2 overexpression and silencing were provided as Fig. S3). The migration, invasion ability and scratch test results of A375 cells (B–D). CCK8 assay of A375 cells after overexpression and silencing of MYBL2 and CDCA8 (n = 3) (E). **P < 0.01, ***P < 0.001, ****P < 0.0001.

We speculated that MYBL2 may be a crucial element in migration, invasion and proliferation activity of A375 cells. We thus assessed A375 cells with overexpression and inhibition of MYBL2 using transwell, scratch, flow cytometry, and other assays. Increased expression of MYBL2 significantly bolstered the migratory and invasive ability of A375 cells. Conversely, the suppression of MYBL2 resulted in a notable reduction in A375 cell migration and invasion. MYBL2 knockdown also impacted cytoskeletal organization and cell motility, leading to alterations in cell morphology and activity (Fig. 4A–D). While flow cytometry revealed a slightly higher number of apoptotic cells in the si-MYBL2 group compared with that in the control group, the difference was not significant (Fig. 4A). CCK8 proliferation assays demonstrated that MYBL2 and CDCA8 silencing inhibited A375 cell proliferation (Fig. 4E). These findings imply a strong association between elevated MYBL2 expression and cellular proliferation, invasion and migration in skin melanoma cell lines.

### 3.5 Animal experiments

**Fig. 5:**
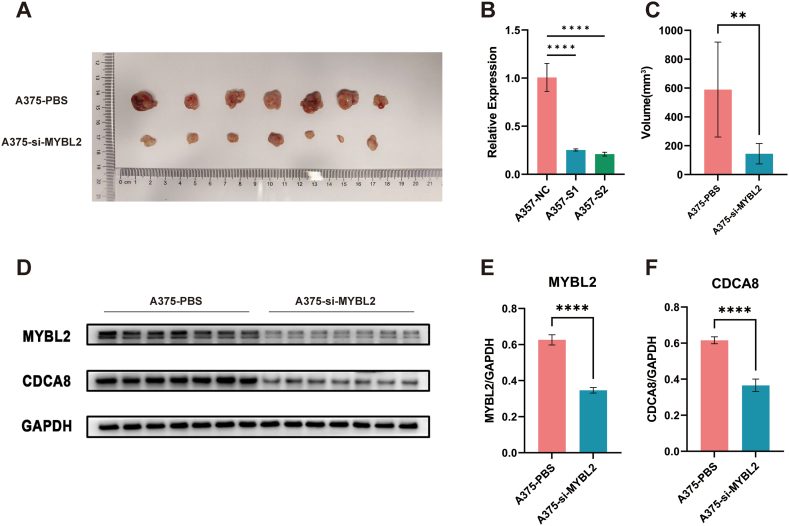
Effects of MYBL2 knockdown on tumor formation in BALB/C nude mice.Tumor sizes in BALB/C nude mice inoculated with A375-PBS or A375-si-MYBL2 cells (A). Validation of si-MYBL2 screening (B). Statistical comparison of tumor sizes (C). MYBL2 and CDCA8 expression in tumor tissues (n = 7) (D–F) (The raw images of Fig. 5D were provided as Fig. S4). **P < 0.01, ***P < 0.001, ****P < 0.0001.

We explored the function of MYBL2 *in vivo*. Mice inoculated with A375-PBS cells exhibited rapid tumor growth and significant tumorigenic effects. In contrast, tumor volume was significantly reduced in mice inoculated with A375 cells with MYBL2 knockdown compared to the group injected with control A375 cells (Fig. 5A–C). These results showed that downregulation of MYBL2 effectively suppressed the proliferation of MM cells. The expressions of MYBL2 and CDCA8 in the tumors were examined; it was indicated that CDCA8 was suppressed in tumors with MYBL2 knockdown (Fig. 5D–F). Together with the outcomes of the in vitro experiments, these findings validate a direct association between MYBL2 and CDCA8 expression in the advancement of malignant melanoma cell proliferation and migration, and CDCA8 could be directly transcriptionally regulated by MYBL2.

### 3.6 The interaction between MYBL2 and CDCA8

**Fig. 6:**
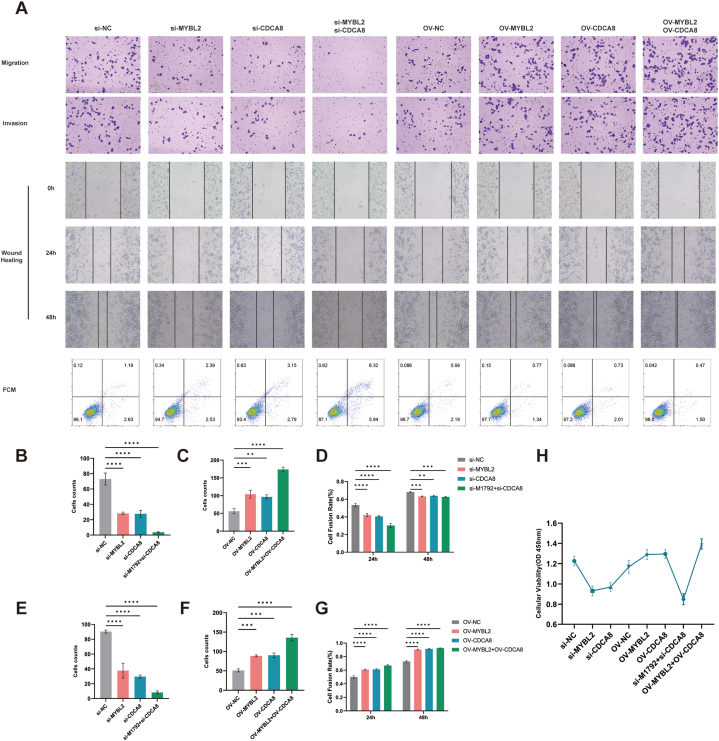
Effects of MYBL2 and CDCA8 on the activity of A375 cells. Detection of migration ability, invasion ability, scratch assay and flow cytometry of A375 cells after MYBL2 and CDCA8 overexpression and silencing (A) (The raw images of migration ability and invasion ability of A375 cells after MYBL2 and CDCA8 overexpression and silencing were provided as Fig. S5. The raw images of scratch assay of A375 cells after MYBL2 and CDCA8 overexpression and silencing were provided as Fig. S6. The raw images of flow cytometry of A375 cells after MYBL2 and CDCA8 overexpression and silencing were provided as Fig. S7). Statistical analysis of migration and invasion experiments of A375 cells after MYBL2 and CDCA8 overexpression and silencing (B–G). CCK8 assays of A375 cells after MYBL2 and CDCA8 overexpression and silencing (H).

To investigate the regulatory mechanism of MYBL2 on CDCA8 expression in human malignant melanoma cells, we transfected MYBL2, CDCA8 genes, and their co-overexpression and knockdown plasmids into A375 cells. In contrast to the untransfected group, there was a notable increase in cell proliferation rate observed in the group overexpressing MYBL2, and the cells exhibited a full and active morphology. Migration and invasion experiments also showed that the MYBL2 overexpression group had significantly enhanced migration and invasion ability (Fig. 6A). Similarly, the CDCA8 overexpression group also exhibited accelerated proliferation. The groups with co-overexpression of MYBL2 and CDCA8 showed the fastest cell proliferation rate compared with groups with single overexpression of MYBL2 or CDCA8 (Fig. 6C–F, H). Cellular morphology changes were also visualized with the highest number of cell clones (Fig. 6G). In contrast, the cell proliferation rate in the MYBL2 knockdown group and CDCA8 knockdown group exhibited a notable decrease (Fig. 6B–E). The cell morphology became flattened, and the mitotic activity decreased. Furthermore, co-knockdown of MYBL2 and CDCA8 resulted in further weakened cell proliferation capacity, irregular cell morphology, and inhibition of mitotic activity (Fig. 6A–D).

## 4 Discussion

MM is a cutaneous malignancy that arises from melanocytes which is highly invasive and prone to metastasis, with poor response to traditional chemotherapy and radiation treatments, resulting in a grim outlook for patients. Recently, there has been a consistent escalation in the global occurrence of MM, imposing a huge economic burden on society [17,18]. Early surgical excision is the primary treatment approach for non-metastatic cutaneous malignant melanoma, yielding favorable survival outcomes [19,20]. In recent years, despite the significant shift in treatment approach for advanced melanoma through the use of targeted therapy and immunotherapy, the risk of mortality in metastatic melanoma remains very high [21]. The absence of specific, early diagnostic, and predictive markers results in most patients presenting with advanced-stage disease at the time of treatment initiation [22]. Hence, early detection and treatment of malignant melanoma is of paramount importance.

MYBL2, belonging to the MYB oncogene family, functions as a transcription factor crucial for regulating cell survival, differentiation and cell cycle progression. Additionally, MYBL2 is implicated in pivotal processes such as DNA impairment mending and spindle shaping, which impact cell cycle progression. MYBL2 activates anti-apoptotic genes like APoJ and Bcl-2, thus inhibiting cell apoptosis. Recent literature had implied that MYBL2 showed high expression in various cancers and negatively associated with survival rates specific to tumors in patients [23,24]. In vitro study had also suggested a close association between MYBL2 with bladder cancer cells's metastasis and proliferation, and inhibiting MYBL2 can substantially reduce proliferation and metastasis [25].

CDCA8, a cytoskeletal protein, plays a role in cytoskeletal assembly and cell movement and has important effects on tumor metastasis and proliferation in breast cancer, bladder cancer [6] and lung cancer [26]. Previous investigation had proved that MYBL2 played a role in proliferation, invasion and migration through the regulation of CDCA8 in breast cancer cell [27]. However, whether MYBL2 and CDCA8 play a role in malignant melanoma of the skin has not been clarified. Herein, we manipulated the MYBL2 and CDCA8 gene expression in A375 cells using overexpression or knockout techniques. The MYBL2 and CDCA8 gene expression levels were significantly higher in A375 cells in comparison to the levels in HaCat cells. Knocking down MYBL2 weakened the A375 cells's invasion and migration ability in vitro, while overexpressing MYBL2 significantly enhanced these abilities. After manipulating MYBL2 expression, We detected a notable affirmative correlation between MYBL2 and both the gene and protein levels of CDCA8. Moreover, it was indicated that low concentrations of MYBL2 were linked to transcriptional dysregulation of DNA repair genes in Bayley et al.’ study [28]. We proposed that CDCA8 may be transcriptionally controlled by MYBL2 in cutaneous malignant melanoma.

We further examined the effects of knocking down MYBL2 on tumor formation in A375 cells through *in vivo* animal experiments. The outcome revealed a notable decrease in tumor volume and CDCA8 protein expression in tumors in the MYBL2 knockdown group in comparison to the findings in the control group. These findings provided further evidence that MYBL2 enhances the invasion, migration and proliferation ability of malignant melanoma cells through the targeting of CDCA8 expression.

## 5 Conclusions

In summary, our research reveals the overexpression of MYBL2 in malignant melanoma cells, which promotes the migration, invasion and proliferation abilities of these cells. Additionally, our study further elucidates the role of MYBL2 in promoting CDCA8 transcription and the role of MYBL2 as an oncogene in CDCA8-dependent cutaneous malignant melanoma. We propose that suppressing MYBL2 expression to modulate and reduce CDCA8 levels may be a strategy for cutaneous malignant melanoma treatment. Thus, efficient CDCA8-targeted therapeutic approaches may represent a strategy to improve patient outcome.

## Data availability statement

The data can be obtained from Mendeley Data at

https://data.mendeley.com/preview/d6pyg7gr3y?a=057f0c85-3158-4716-9ea0-0ddf6dc27326.

## CRediT authorship contribution statement

**Chen Wu:** Writing – original draft. **Jiahui Jiang:** Conceptualization. **Chao Ci:** Writing – review & editing.

## Declaration of competing interest

The authors declare that they have no known competing financial interests or personal relationships that could have appeared to influence the work reported in this paper.

## Figures and Tables

**Figure:**
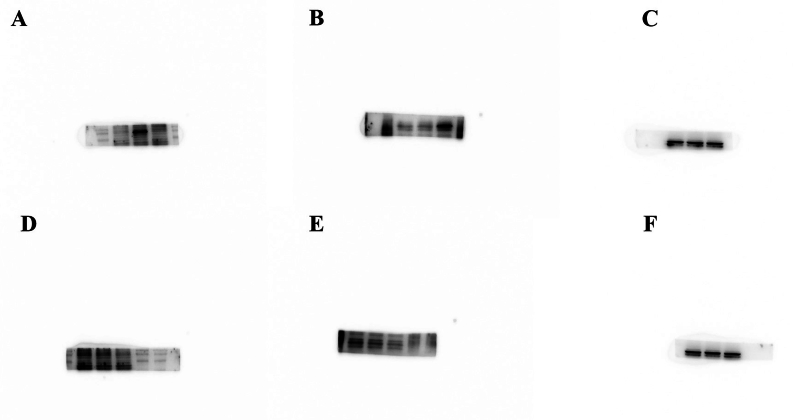
Protein expression of MYBL2 after overexpression of both MYBL2 and CDCA8 (A). Protein expression of CDCA8 after overexpression of both MYBL2 and CDCA8 (B). Protein expression of β-tubulin after overexpression of both MYBL2 and CDCA8 (C). Protein expression of MYBL2 after silence of both MYBL2 and CDCA8 (D). Protein expression of CDCA8 after silence of both MYBL2 and CDCA8 (E). Protein expression of β-tubulin after silence of both MYBL2 and CDCA8 (F).

**Figure:**
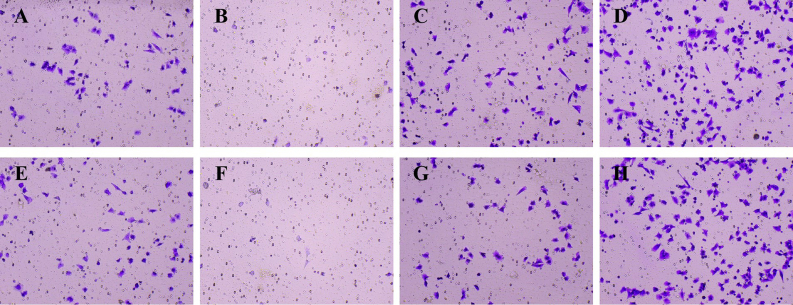
The migration ability of A375 cell of silence control group (A), MYBL2 silencing (B), overexpression control group (C) and MYBL2 overexpression (D). The invasion ability of A375 cell of silence control group (E), MYBL2 silencing (F), overexpression control group (G) and MYBL2 overexpression (H).

**Figure:**
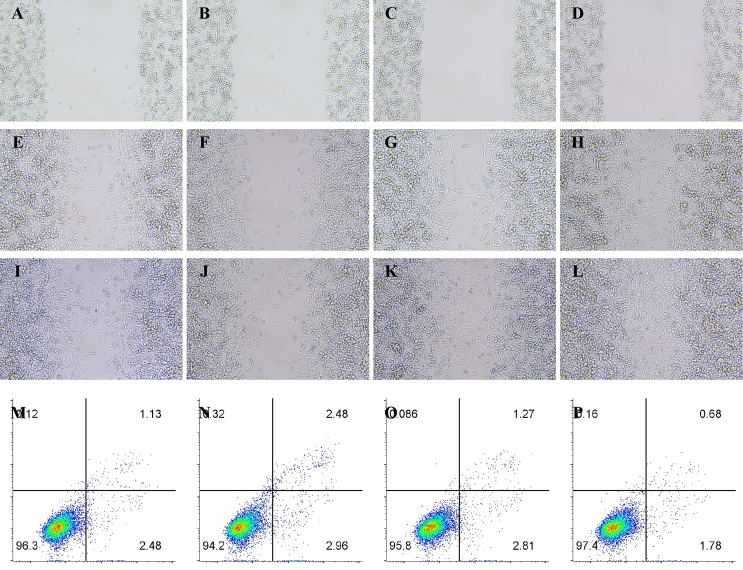
The scratch assay of A375 cell of silence control group (A), MYBL2 silencing (B), overexpression control group (C) and MYBL2 overexpression (D) at 0 h. The scratch assay of A375 cell of silence control group (E), MYBL2 silencing (F), overexpression control group (G) and MYBL2 overexpression (H) at 24 h. The scratch assay of A375 cell of silence control group (I), MYBL2 silencing (J), overexpression control group (K) and MYBL2 overexpression (L) at 48 h. The flow cytometry of A375 cell of silence control group (M), MYBL2 silencing (N), overexpression control group (O) and MYBL2 overexpression (P).

**Figure:**
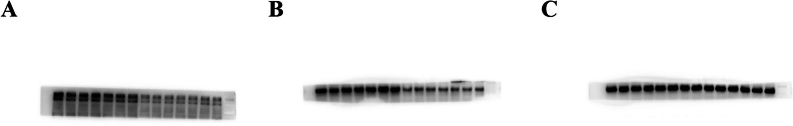
The raw images showcasing the protein expression levels of MYBL2 (A), CDCA8 (B) and GAPDH (C) in tumor tissues as well as their adjacent normal tissues.

**Figure:**
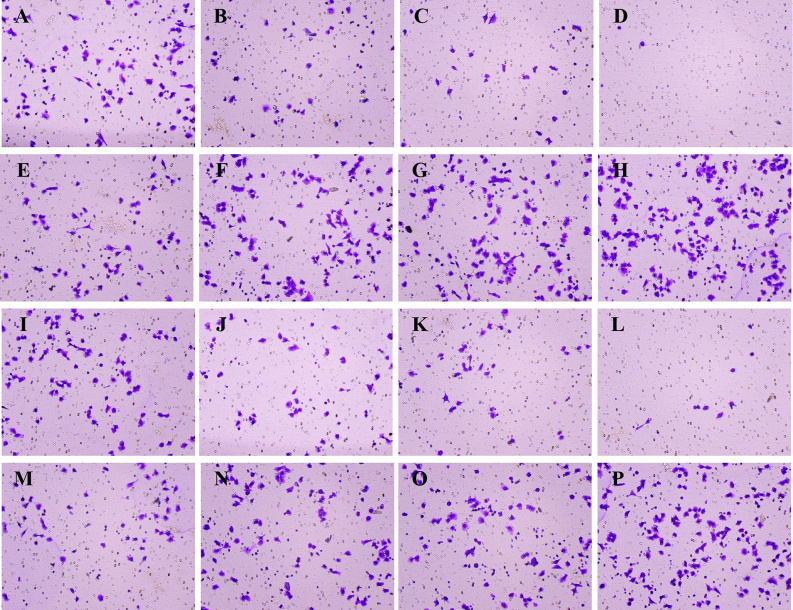
The migration ability of A375 cell of silence control group (A), MYBL2 silencing (B), CDCA8 silencing (C), MYBL2 and CDCA8 both silencing (D), overexpression control group (E), MYBL2 overexpression (F), CDCA8 overexpression (G) and MYBL2 and CDCA8 both overexpression (H). The invasion ability of A375 cell of silence control group (I), MYBL2 silencing (J), CDCA8 silencing (K), MYBL2 and CDCA8 both silencing (L), overexpression control group (M), MYBL2 overexpression (N), CDCA8 overexpression (O) and MYBL2 and CDCA8 both overexpression (P).

**Figure:**
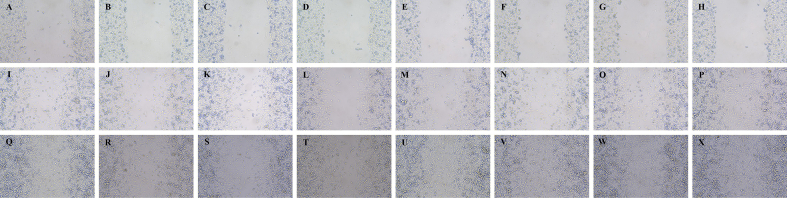
The scratch assay of A375 cell of silence control group (A), MYBL2 silencing (B), CDCA8 silencing (C), MYBL2 and CDCA8 both silencing (D), overexpression control group (E), MYBL2 overexpression (F), CDCA8 overexpression (G) and MYBL2 and CDCA8 both overexpression (H) at 0 h. The scratch assay of A375 cell of silence control group (I), MYBL2 silencing (J), CDCA8 silencing (K), MYBL2 and CDCA8 both silencing (L), overexpression control group (M), MYBL2 overexpression (N), CDCA8 overexpression (O) and MYBL2 and CDCA8 both overexpression (P) at 24 h. The scratch assay of A375 cell of silence control group (Q), MYBL2 silencing (R), CDCA8 silencing (S), MYBL2 and CDCA8 both silencing (T), overexpression control group (U), MYBL2 overexpression (V), CDCA8 overexpression (W) and MYBL2 and CDCA8 both overexpression (X) at 48 h.

**Figure:**
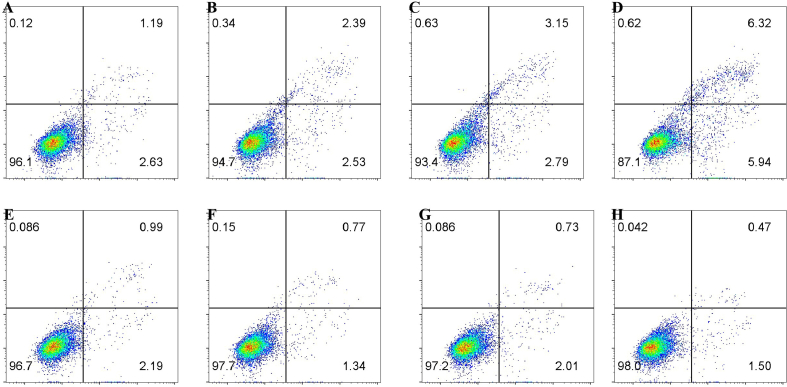
The flow cytometry of A375 cell of silence control group (A), MYBL2 silencing (B), CDCA8 silencing (C), MYBL2 and CDCA8 both silencing (D), overexpression control group (E), MYBL2 overexpression (F), CDCA8 overexpression (G) and MYBL2 and CDCA8 both overexpression (H).
