# Supplementary figures and images for: The correlation between the MYBL2/CDCA8 signaling pathway of malignant melanoma
Source: Heliyon. 2024 Jun 5;10(11):e32485. doi: 10.1016/j.heliyon.2024.e32485 (PMC11219495; doi:10.1016/j.heliyon.2024.e32485)

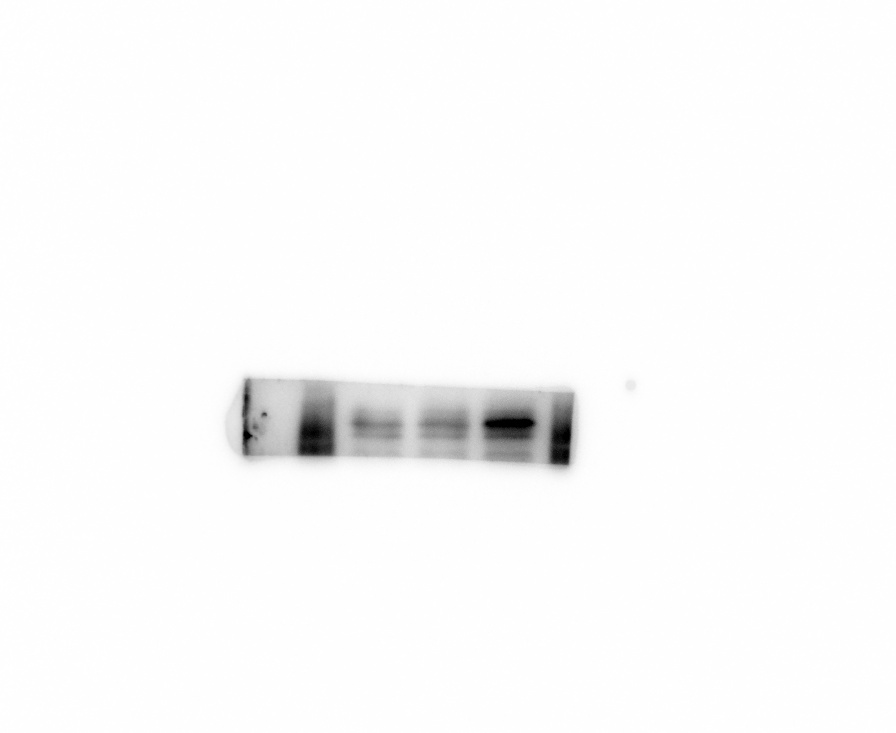

Supplement: Multimedia component 1 [file mmc1.zip › the raw data/Figure 3C/CDCA8-OV.jpg]

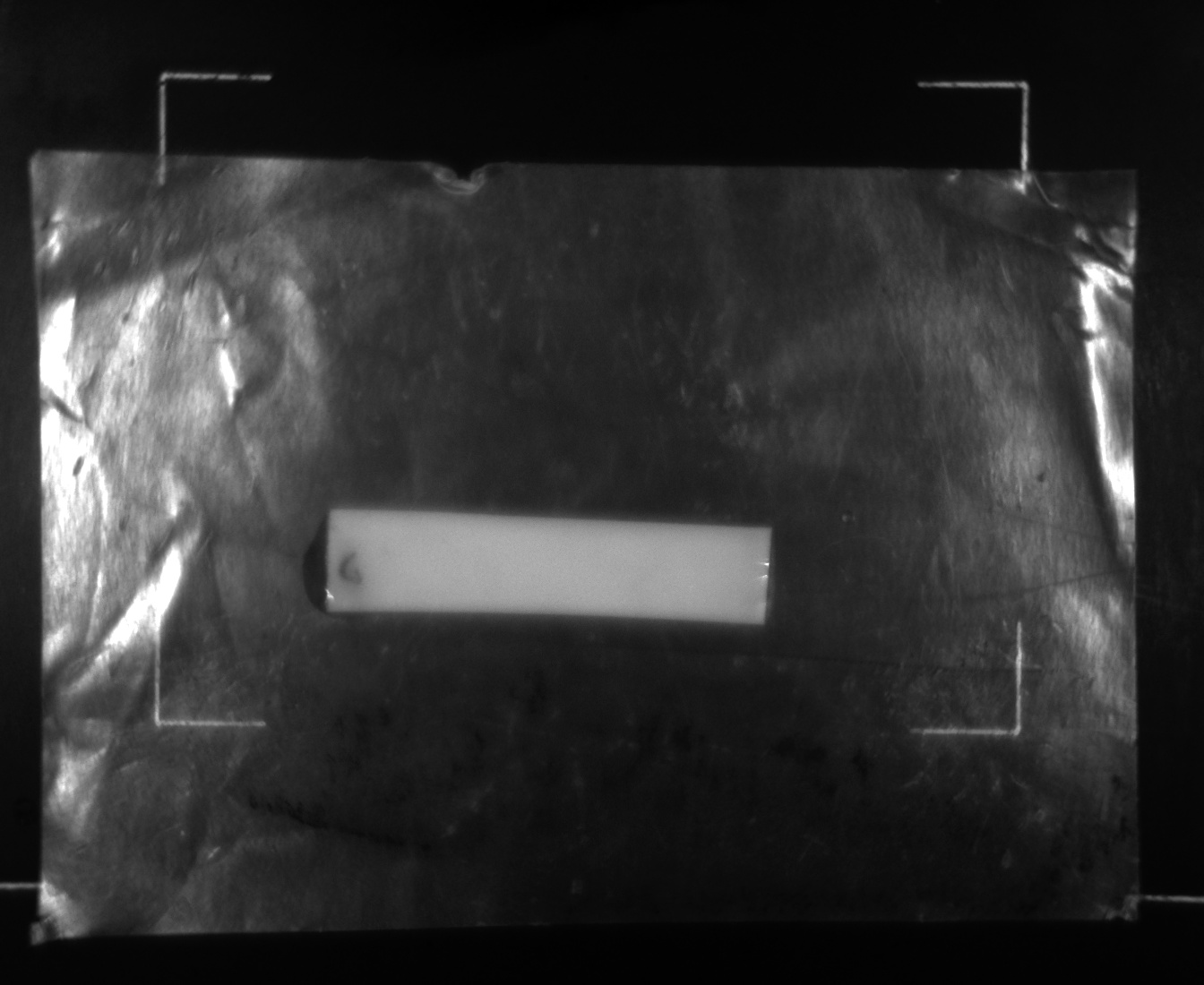

Supplement: Multimedia component 1 [file mmc1.zip › the raw data/Figure 3C/CDCA8-OV-bright-field image of PVDF membrane.jpg]

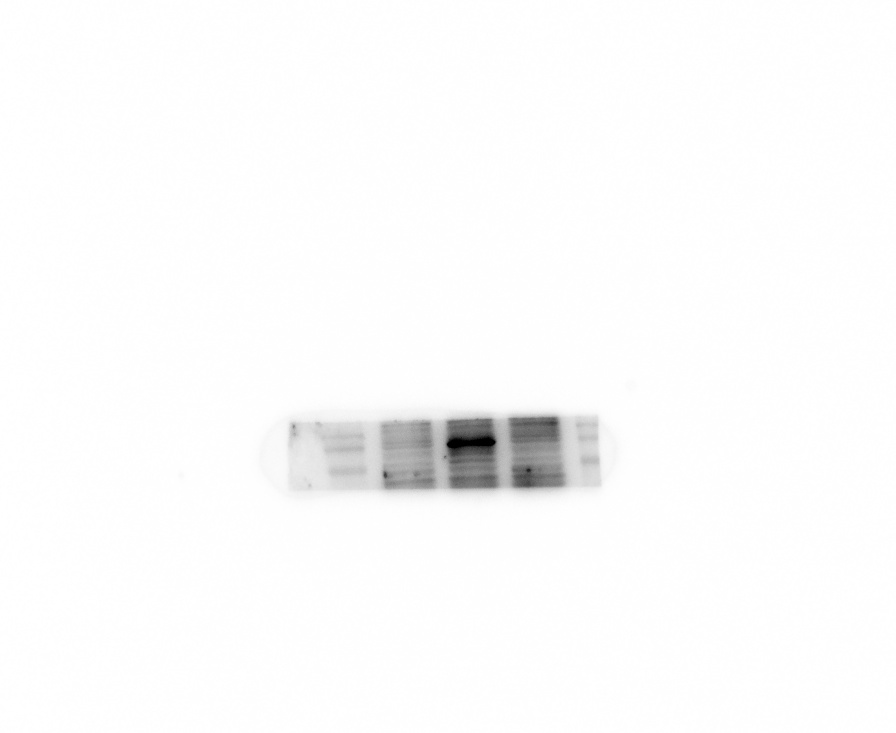

Supplement: Multimedia component 1 [file mmc1.zip › the raw data/Figure 3C/MYBL2-OV.jpg]

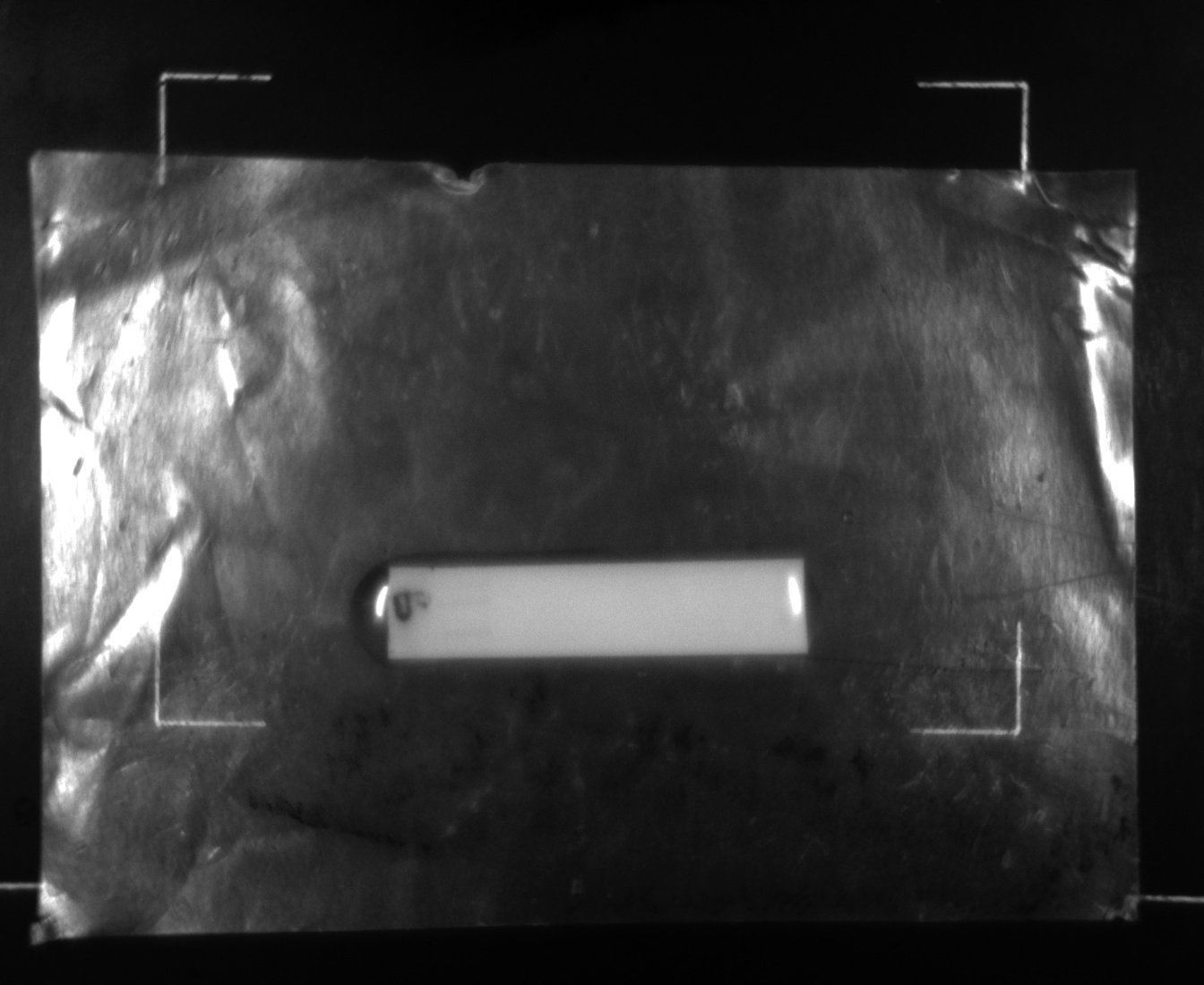

Supplement: Multimedia component 1 [file mmc1.zip › the raw data/Figure 3C/MYBL2-OV-bright-field image of PVDF membrane.jpg]

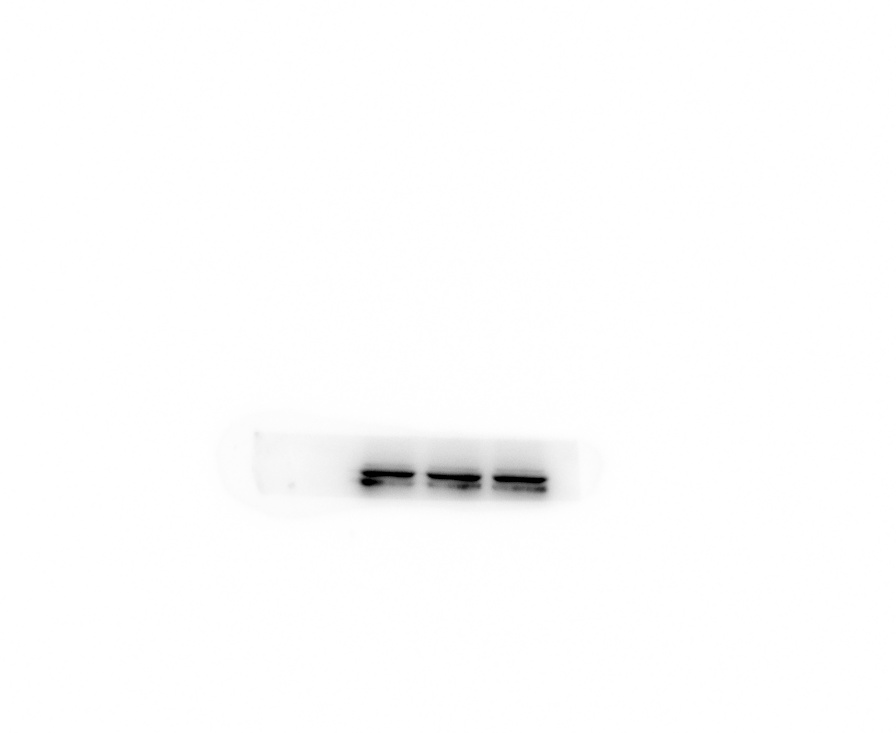

Supplement: Multimedia component 1 [file mmc1.zip › the raw data/Figure 3C/a┬-tubulin-OV.jpg]

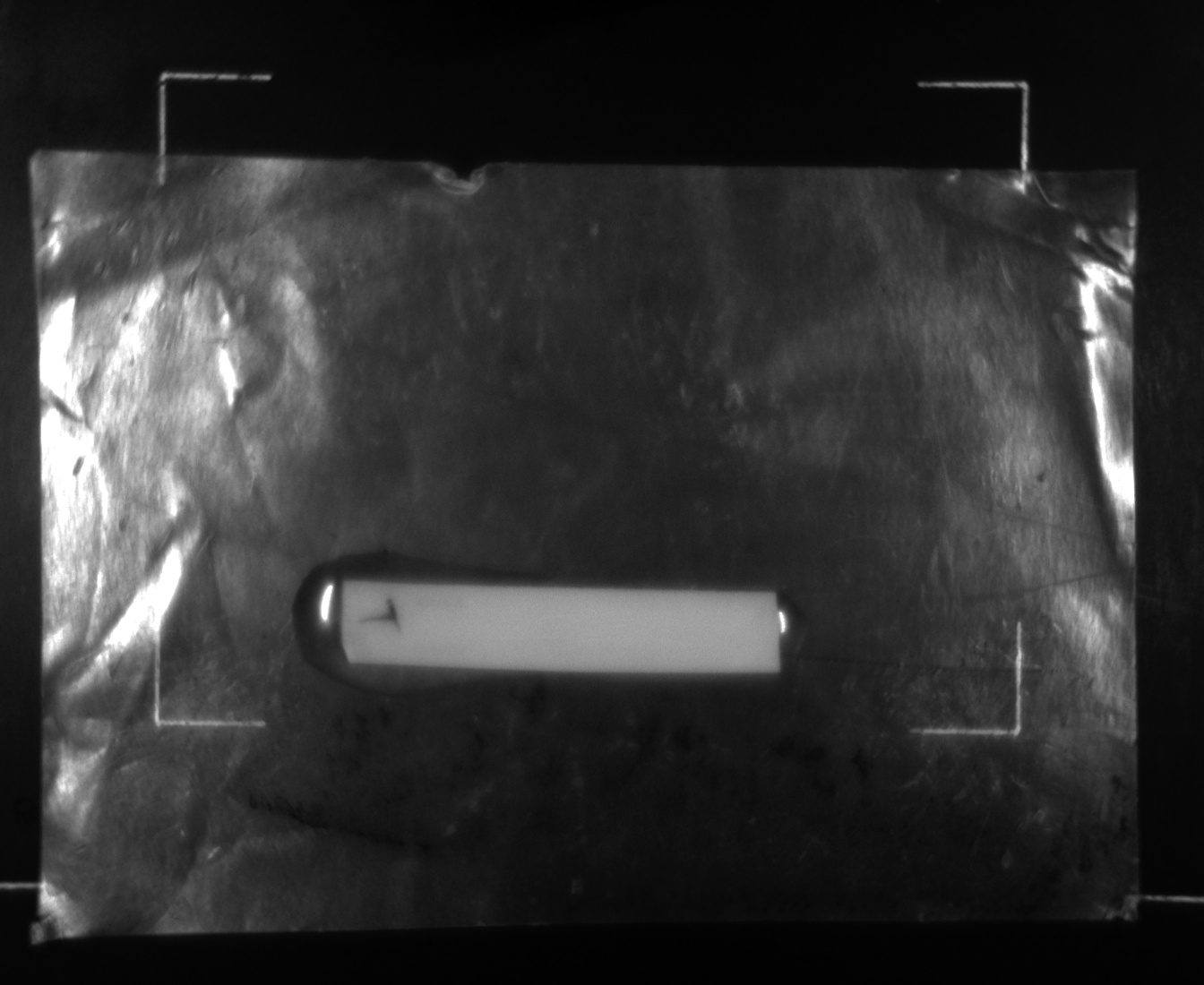

Supplement: Multimedia component 1 [file mmc1.zip › the raw data/Figure 3C/a┬-tubulin-OV-bright-field image of PVDF membrane.jpg]

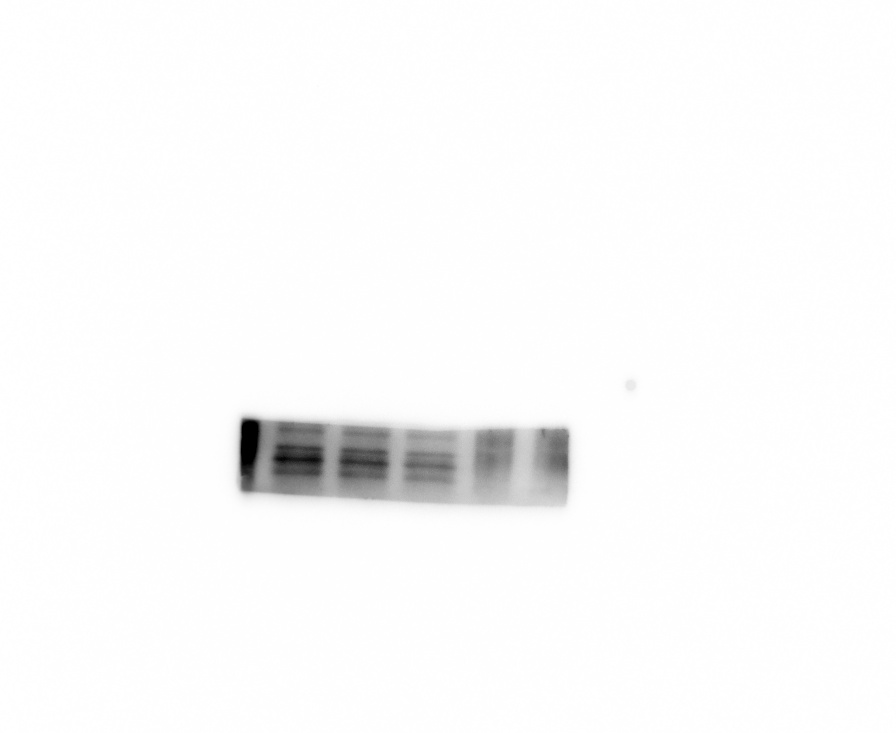

Supplement: Multimedia component 1 [file mmc1.zip › the raw data/Figure 3D/CDCA8-Si.jpg]

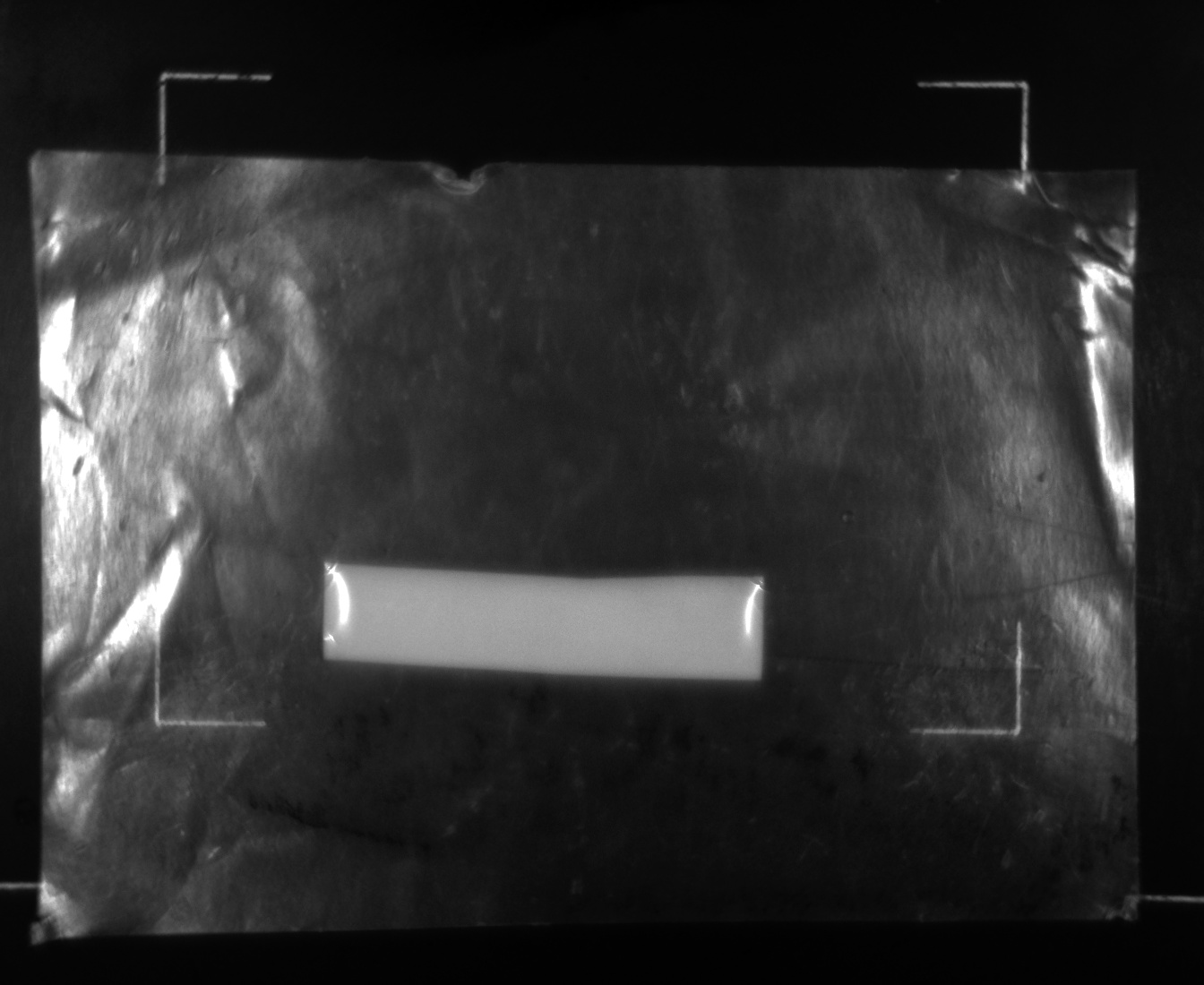

Supplement: Multimedia component 1 [file mmc1.zip › the raw data/Figure 3D/CDCA8-Si-bright-field image of PVDF membrane.jpg]

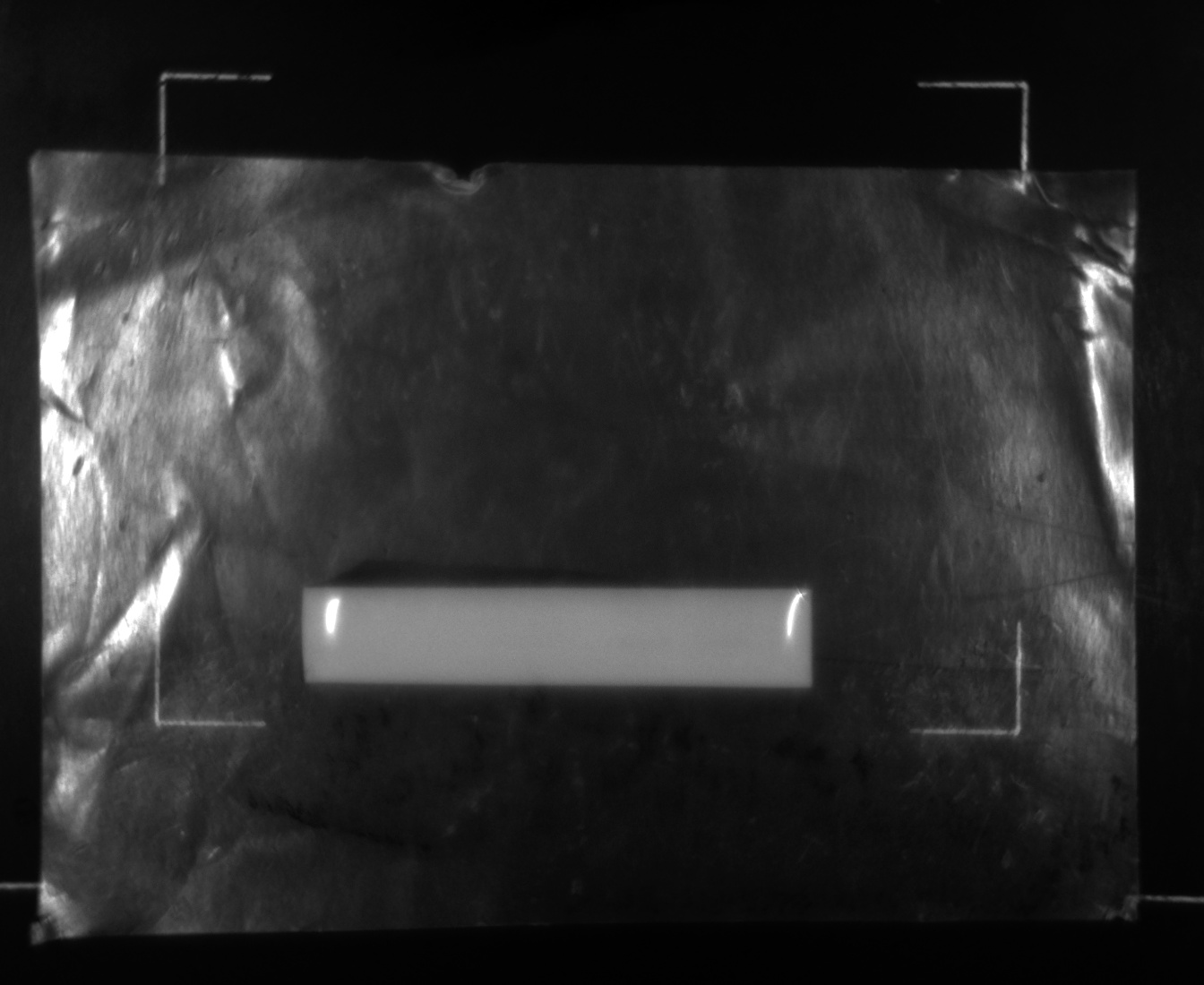

Supplement: Multimedia component 1 [file mmc1.zip › the raw data/Figure 3D/MYBL2-Si- bright-field image of PVDF membrane.jpg]

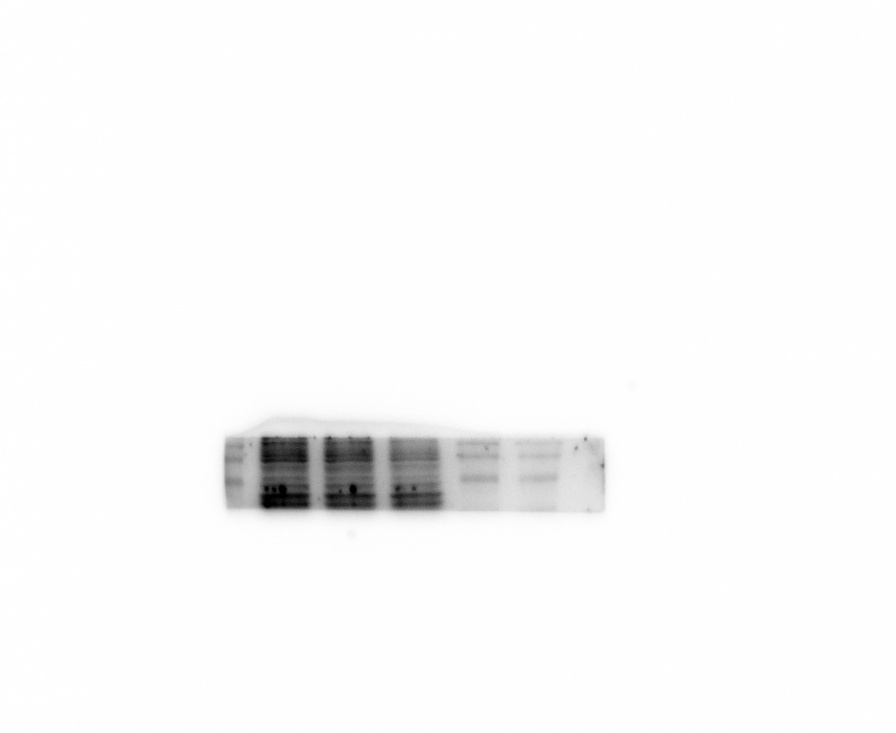

Supplement: Multimedia component 1 [file mmc1.zip › the raw data/Figure 3D/MYBL2-Si.jpg]

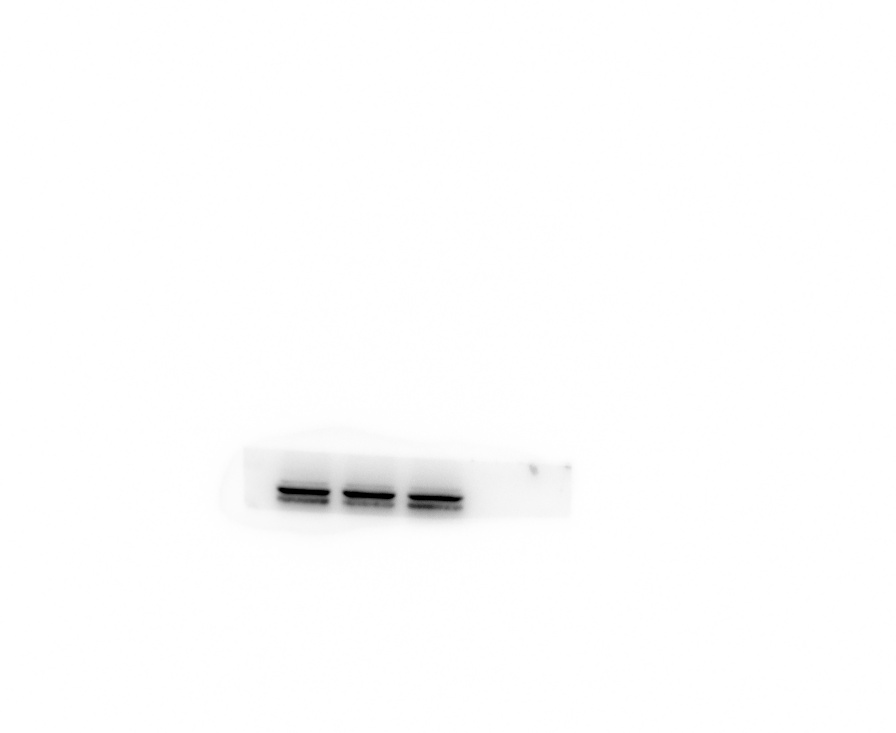

Supplement: Multimedia component 1 [file mmc1.zip › the raw data/Figure 3D/a┬-tubulin-Si.jpg]

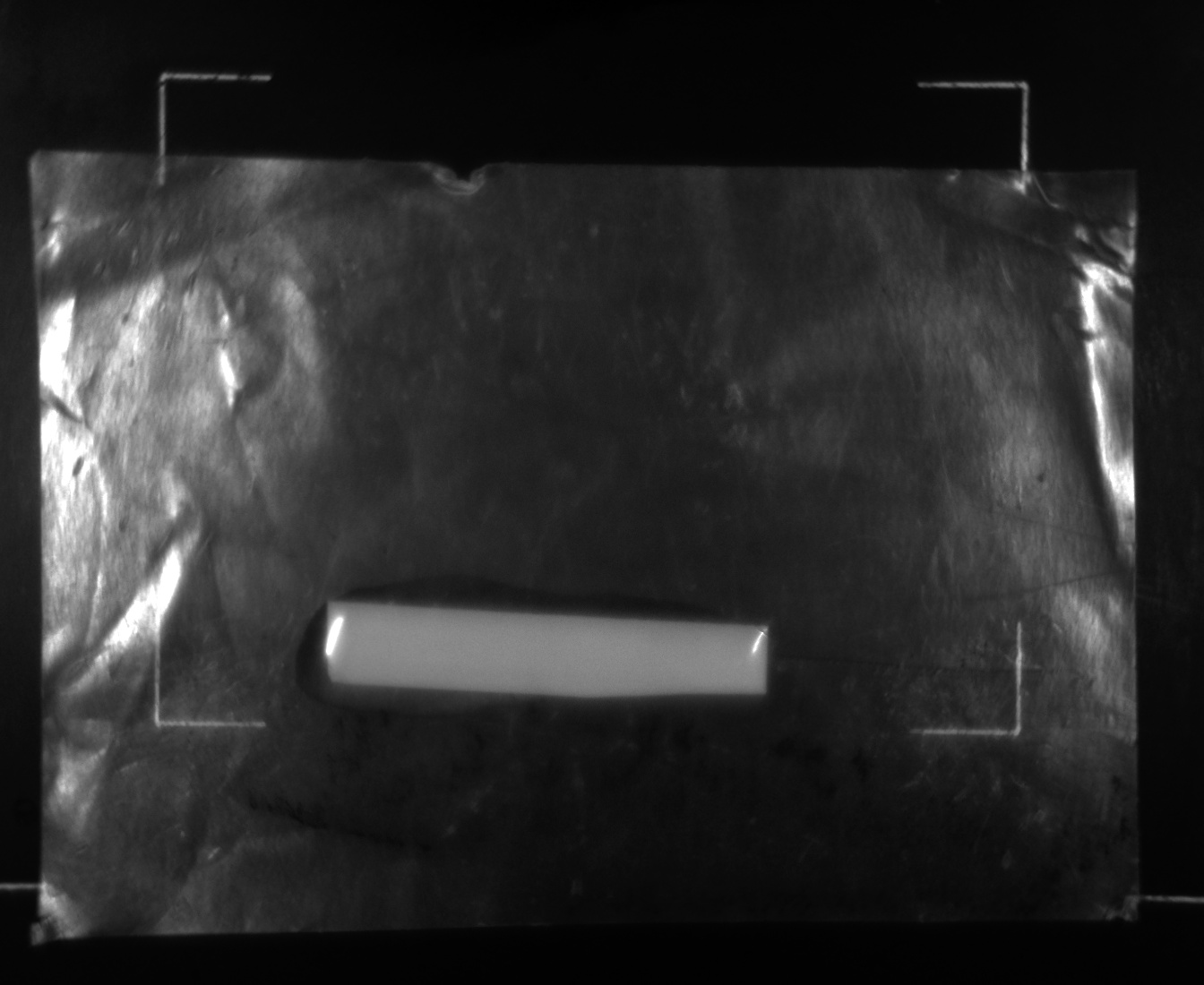

Supplement: Multimedia component 1 [file mmc1.zip › the raw data/Figure 3D/a┬-tubulin-Si-bright-field image of PVDF membrane.jpg]

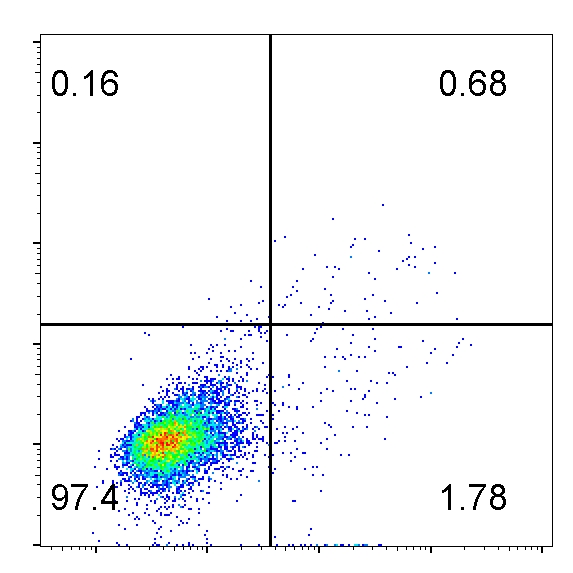

Supplement: Multimedia component 1 [file mmc1.zip › the raw data/Figure 4A/Figure 4 A FCM/OV-MYBL2.jpg]

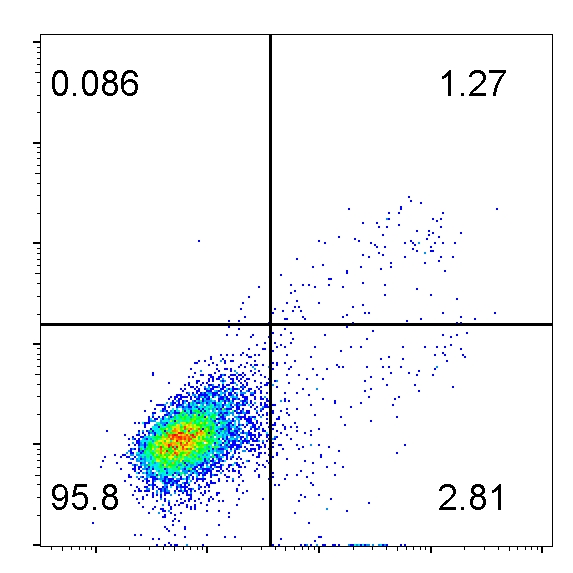

Supplement: Multimedia component 1 [file mmc1.zip › the raw data/Figure 4A/Figure 4 A FCM/OV-NC.jpg]

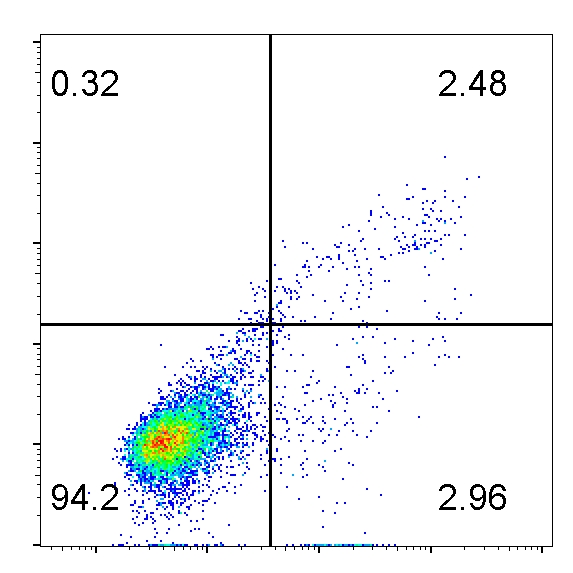

Supplement: Multimedia component 1 [file mmc1.zip › the raw data/Figure 4A/Figure 4 A FCM/Si-MYBL2.jpg]

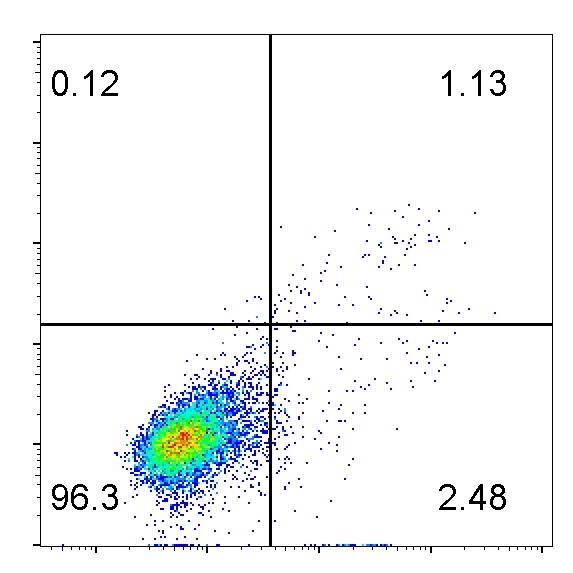

Supplement: Multimedia component 1 [file mmc1.zip › the raw data/Figure 4A/Figure 4 A FCM/Si-NC.jpg]

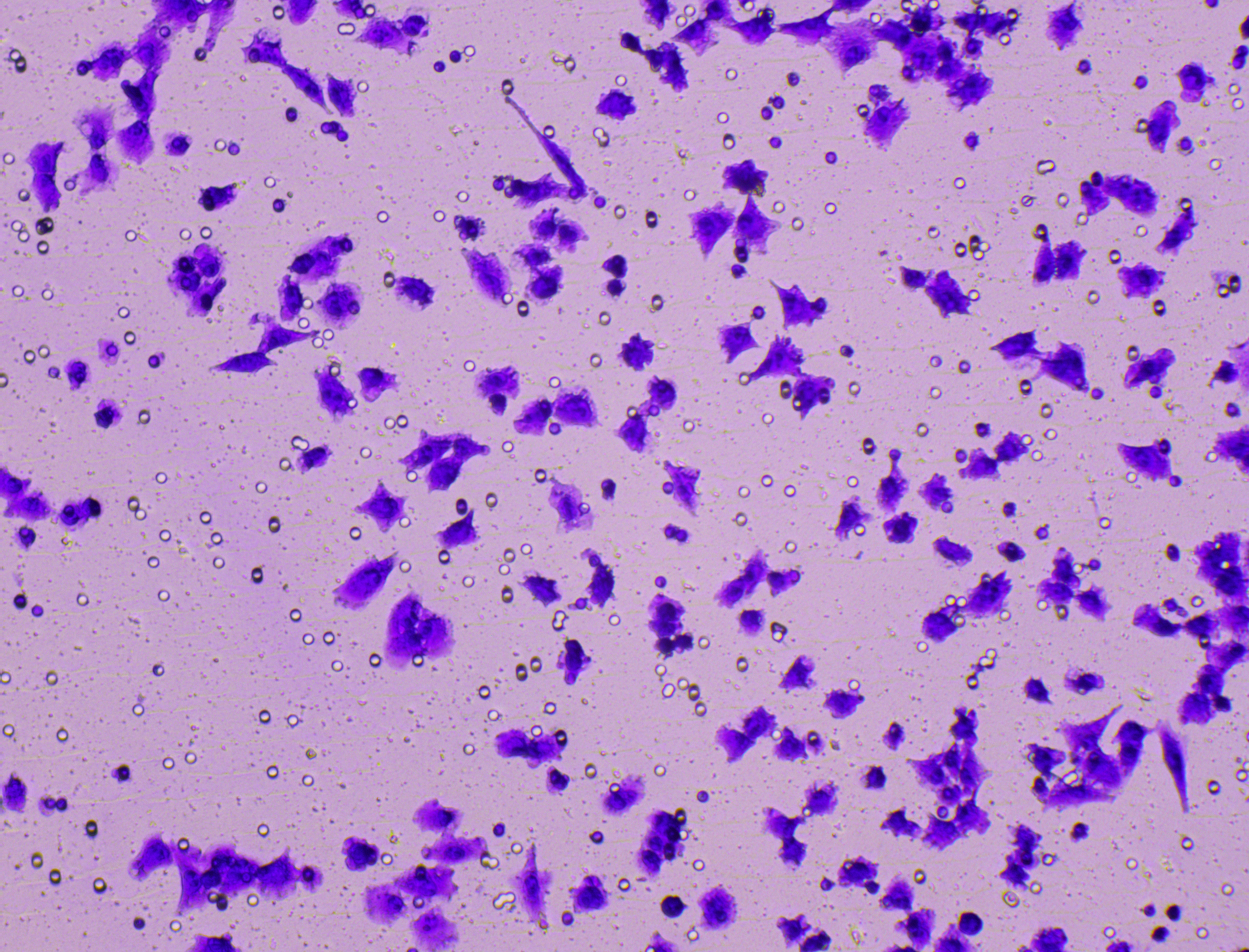

Supplement: Multimedia component 1 [file mmc1.zip › the raw data/Figure 4A/Figure 4A invasion/OV-MBYL2.jpg]

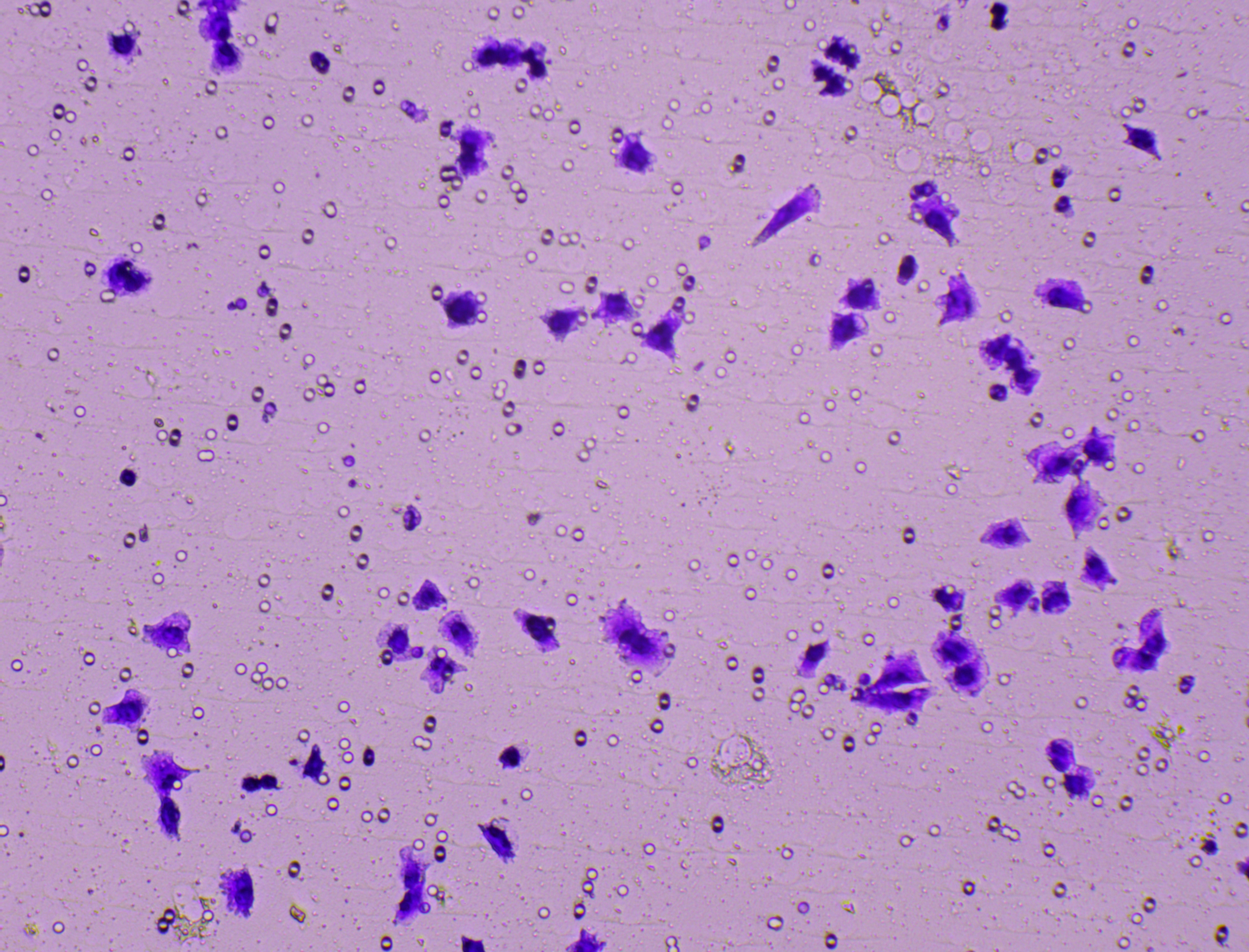

Supplement: Multimedia component 1 [file mmc1.zip › the raw data/Figure 4A/Figure 4A invasion/OV-NC.jpg]

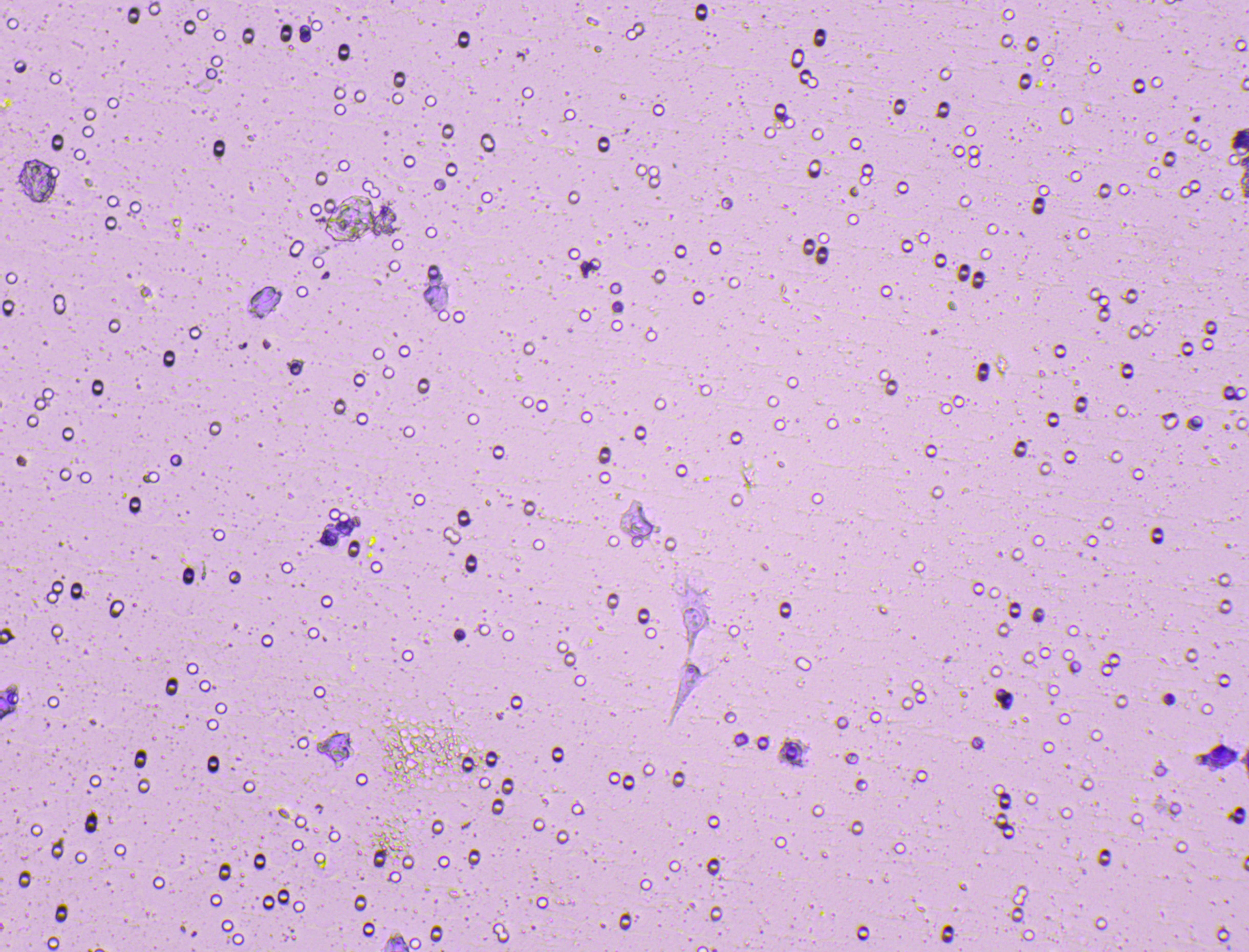

Supplement: Multimedia component 1 [file mmc1.zip › the raw data/Figure 4A/Figure 4A invasion/si-MBYL2.jpg]

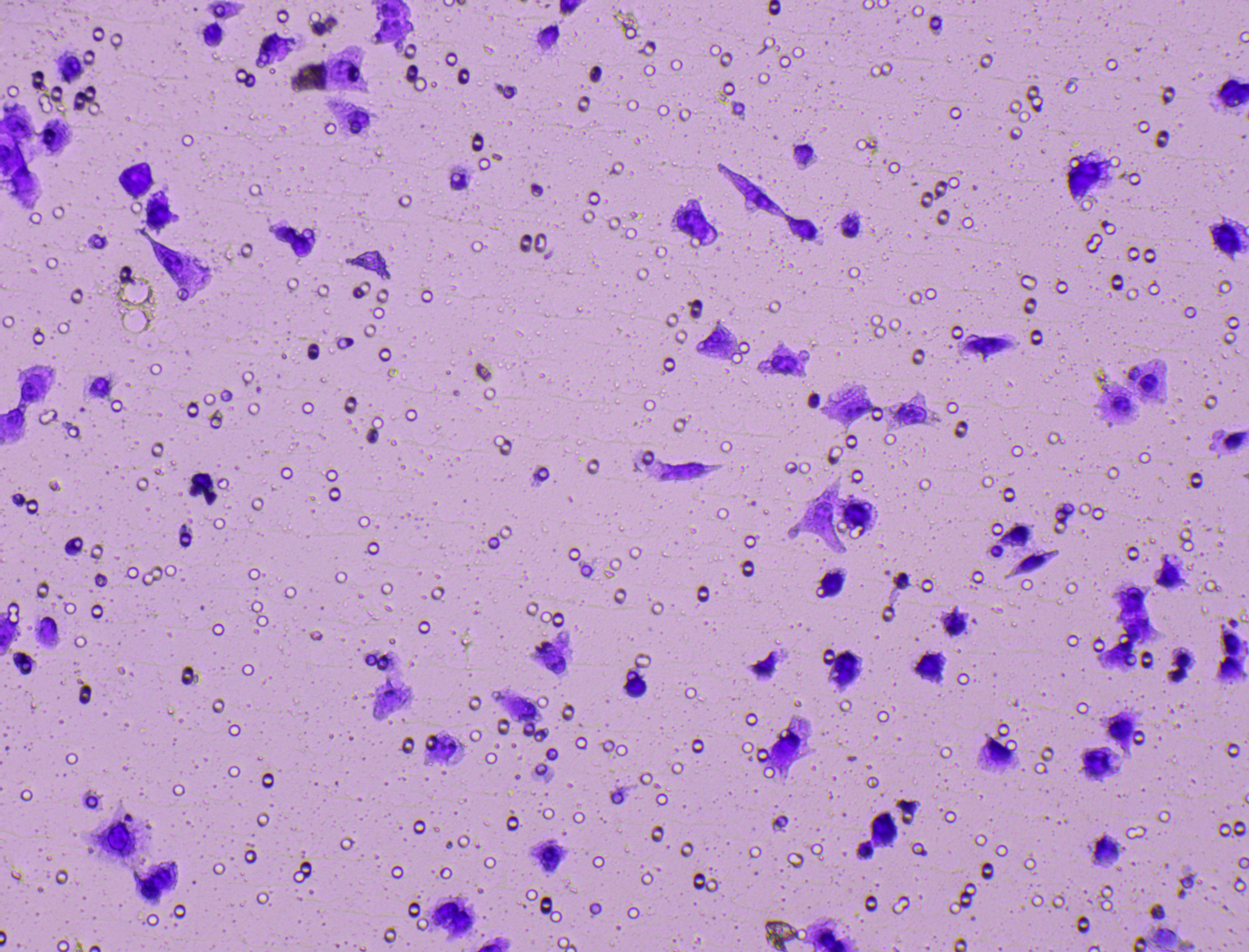

Supplement: Multimedia component 1 [file mmc1.zip › the raw data/Figure 4A/Figure 4A invasion/Si-NC.jpg]

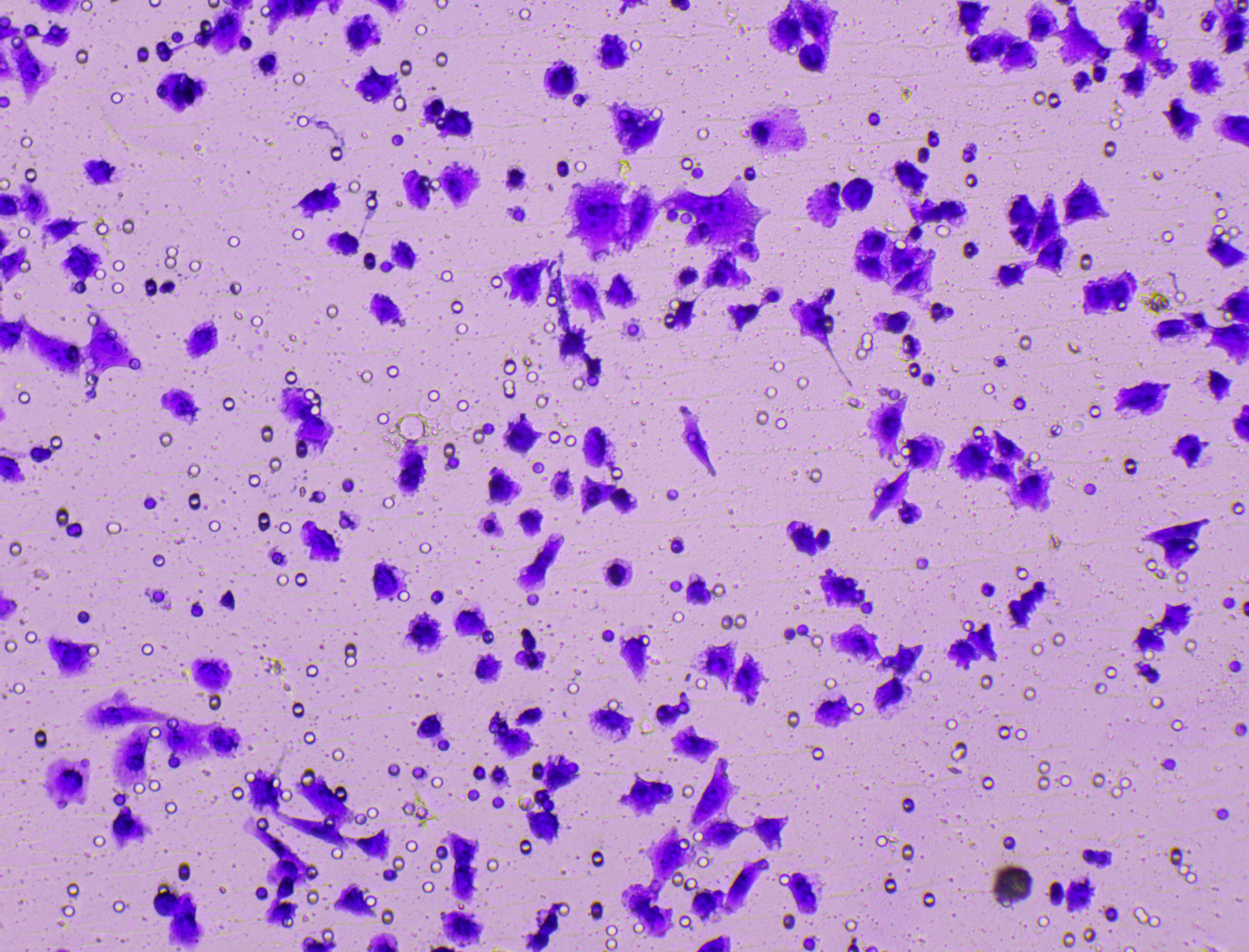

Supplement: Multimedia component 1 [file mmc1.zip › the raw data/Figure 4A/Figure 4A migiration/OV-MBYL2.jpg]

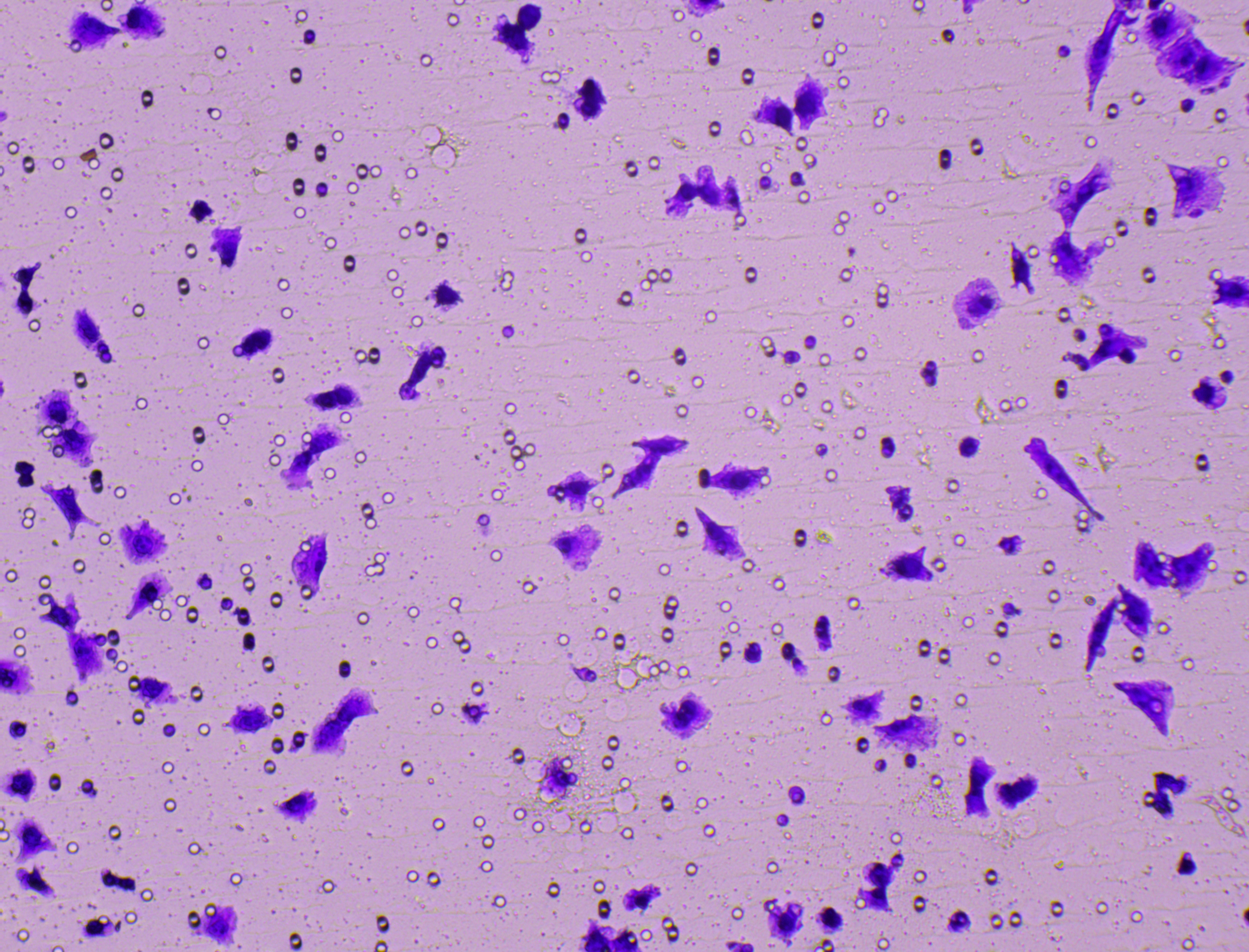

Supplement: Multimedia component 1 [file mmc1.zip › the raw data/Figure 4A/Figure 4A migiration/OV-NC.jpg]

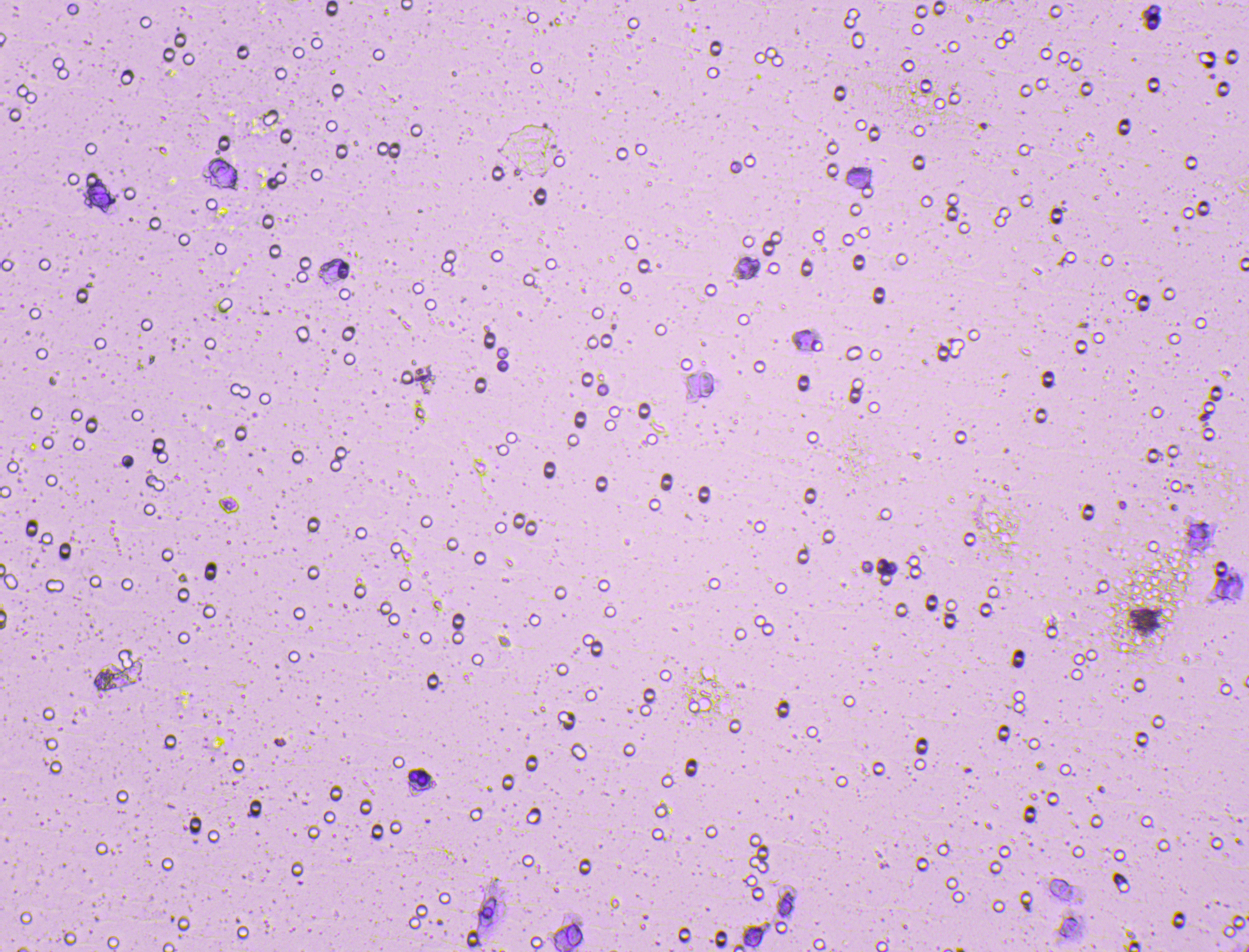

Supplement: Multimedia component 1 [file mmc1.zip › the raw data/Figure 4A/Figure 4A migiration/si-MBYL2.jpg]

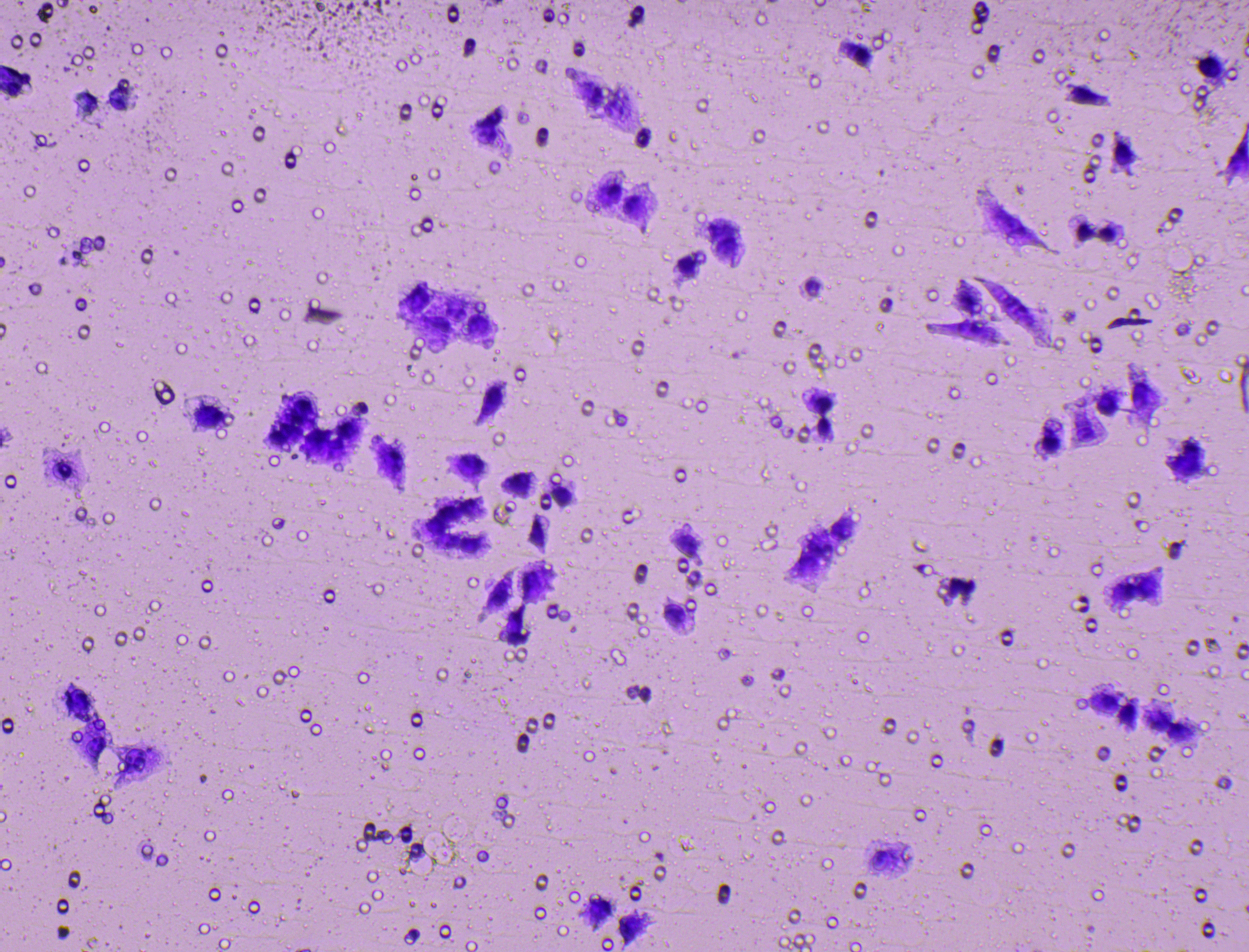

Supplement: Multimedia component 1 [file mmc1.zip › the raw data/Figure 4A/Figure 4A migiration/Si-NC.jpg]

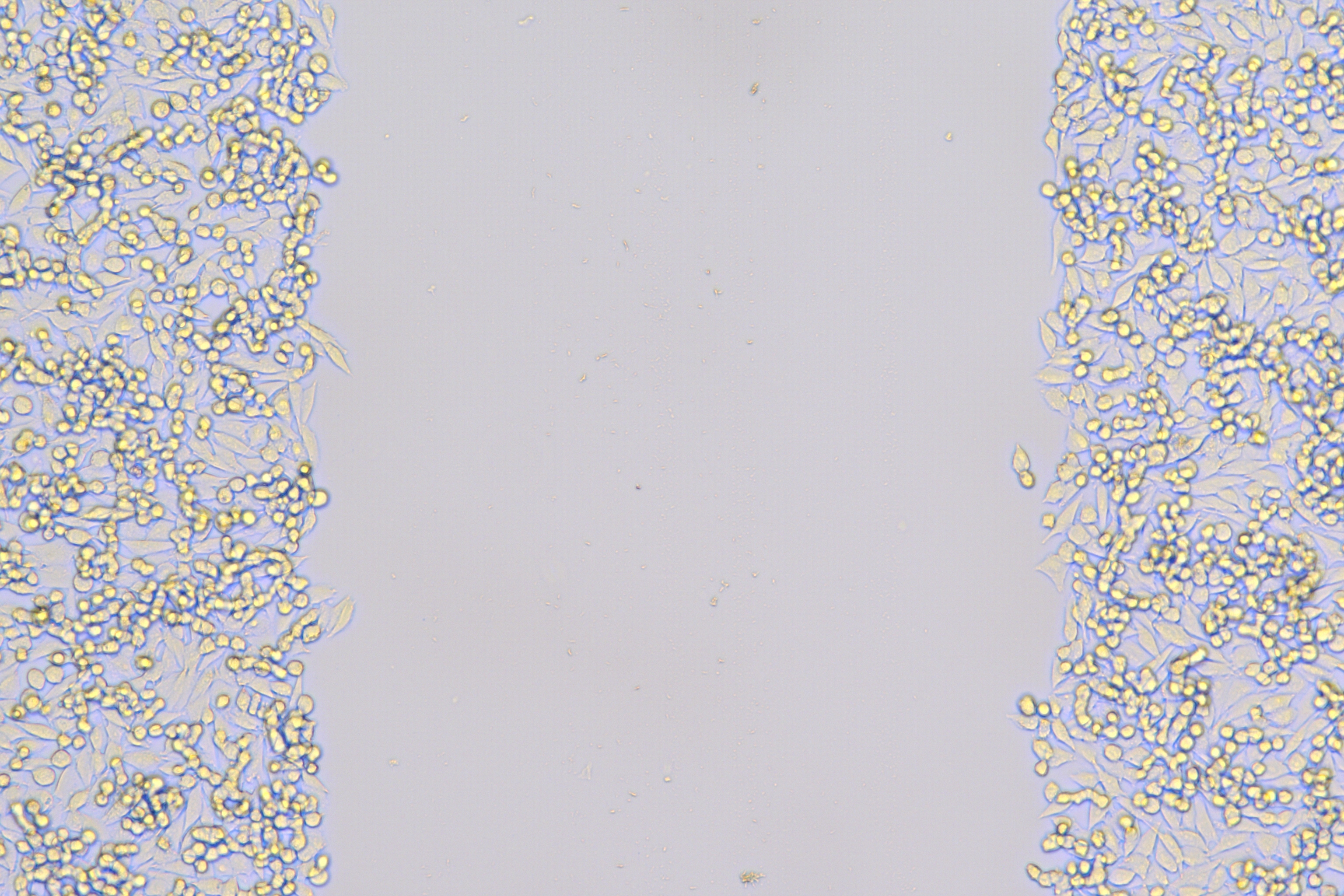

Supplement: Multimedia component 1 [file mmc1.zip › the raw data/Figure 4A/Figure 4A wound healing/0 h/OV-MYBL2.jpg]

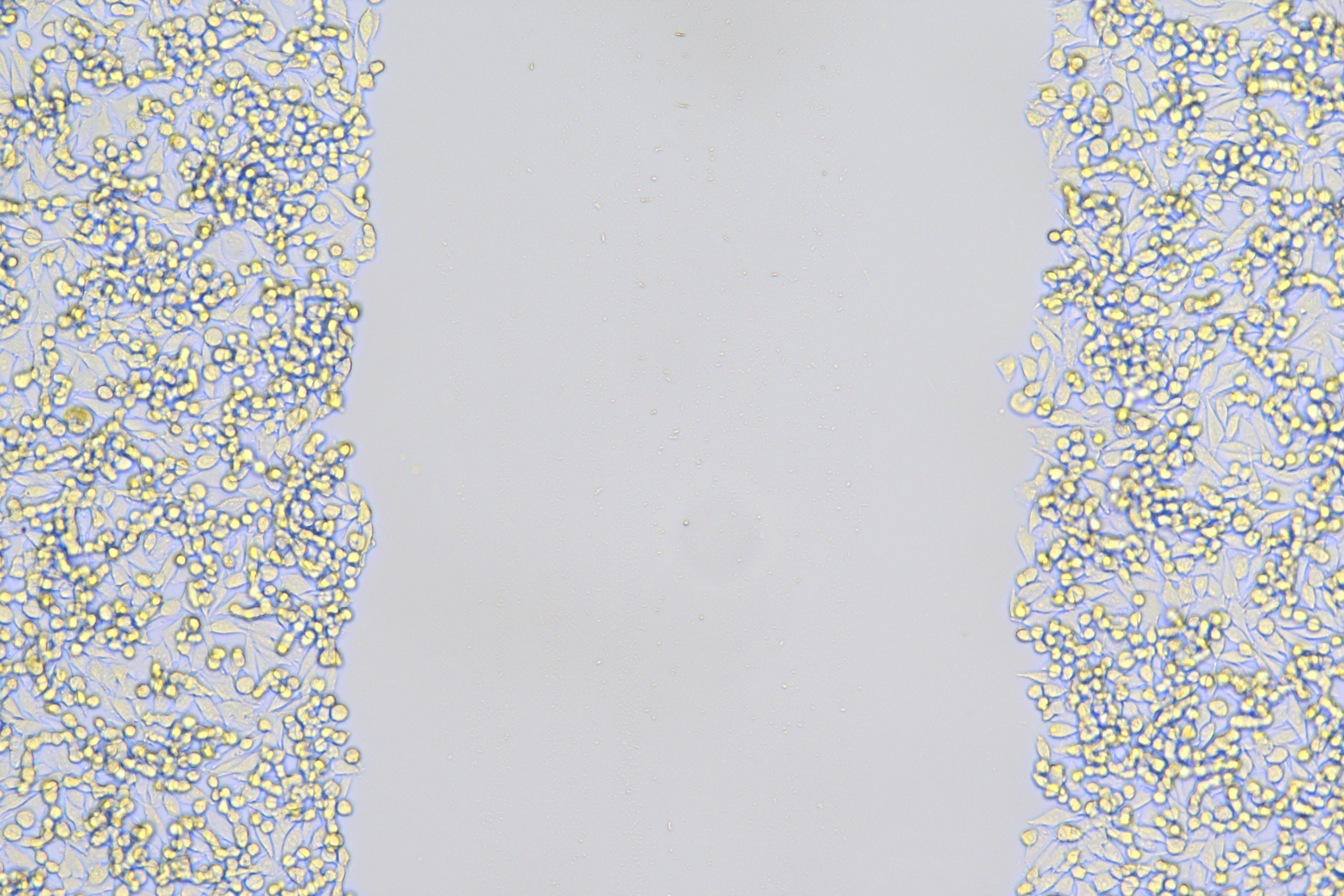

Supplement: Multimedia component 1 [file mmc1.zip › the raw data/Figure 4A/Figure 4A wound healing/0 h/OV-NC.jpg]

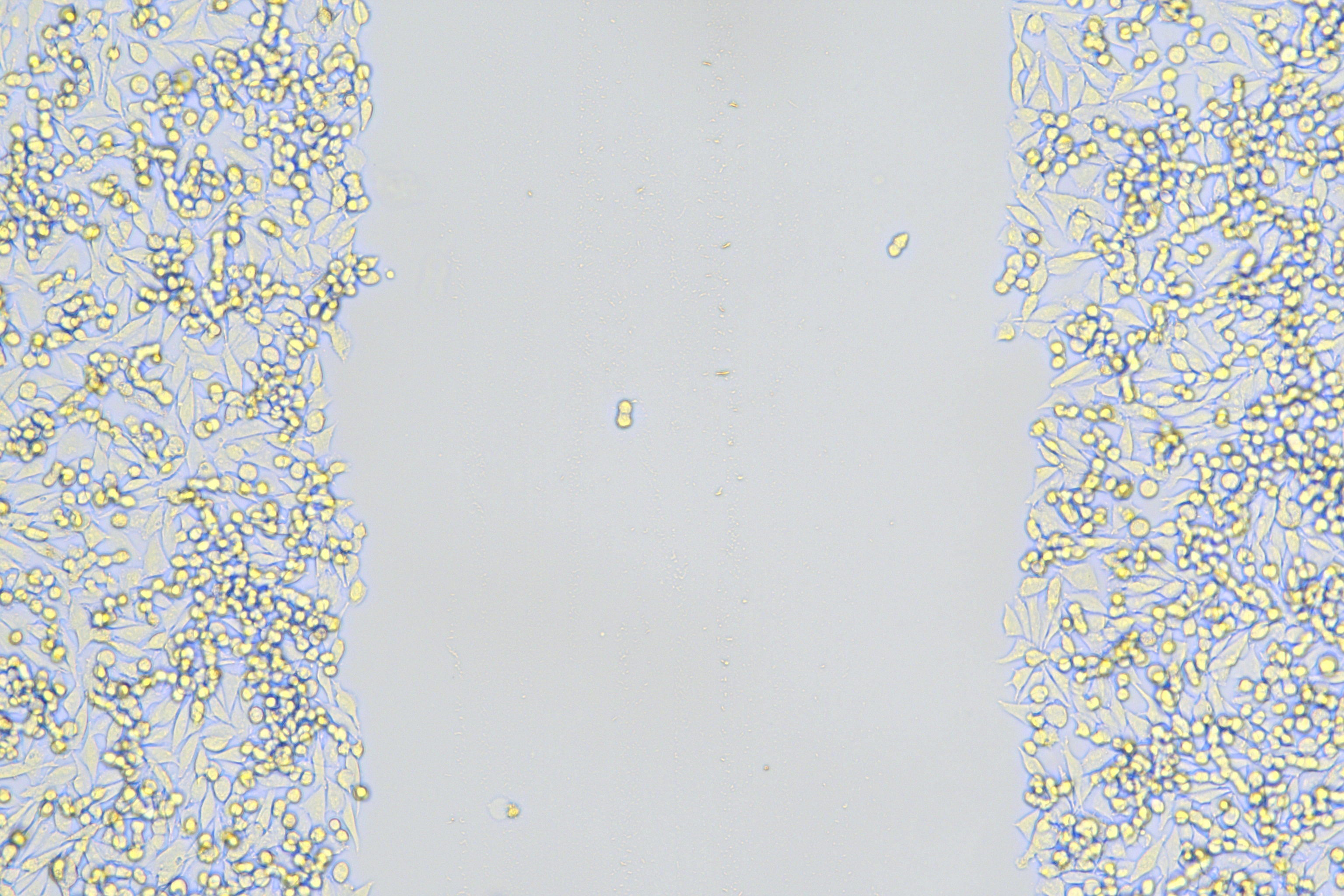

Supplement: Multimedia component 1 [file mmc1.zip › the raw data/Figure 4A/Figure 4A wound healing/0 h/Si-MYBL2.jpg]

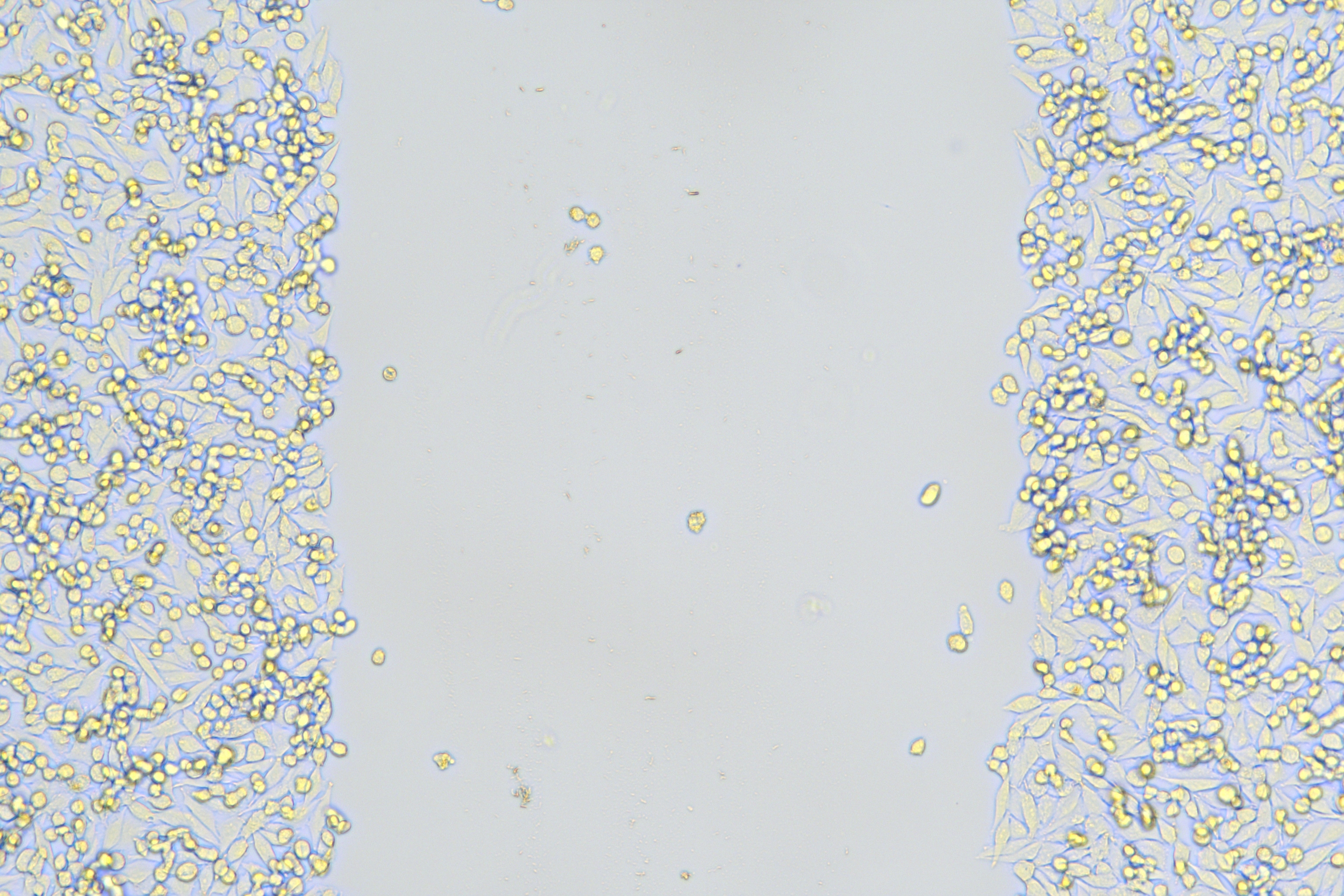

Supplement: Multimedia component 1 [file mmc1.zip › the raw data/Figure 4A/Figure 4A wound healing/0 h/Si-NC.jpg]

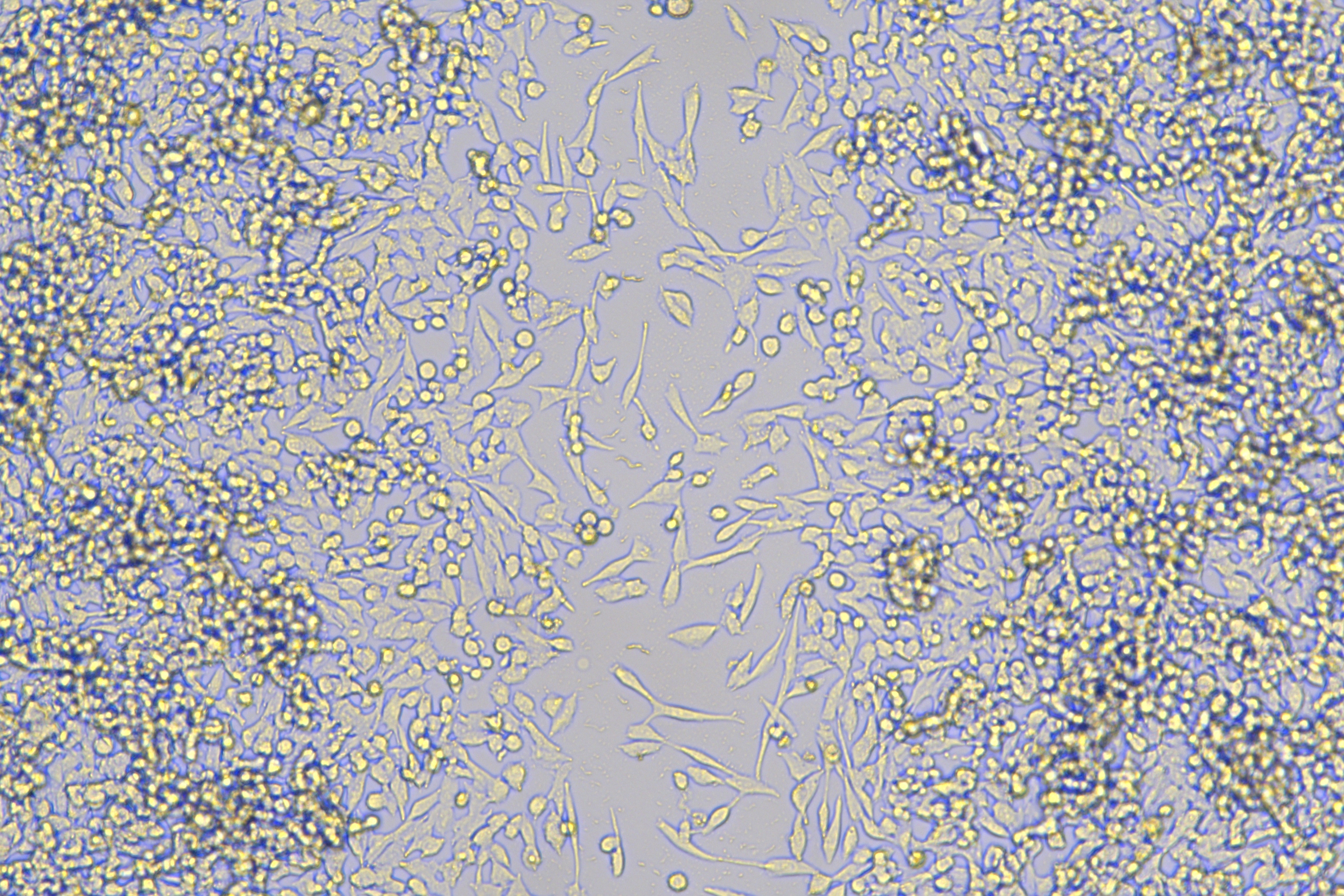

Supplement: Multimedia component 1 [file mmc1.zip › the raw data/Figure 4A/Figure 4A wound healing/24 h/OV-MYBL2.jpg]

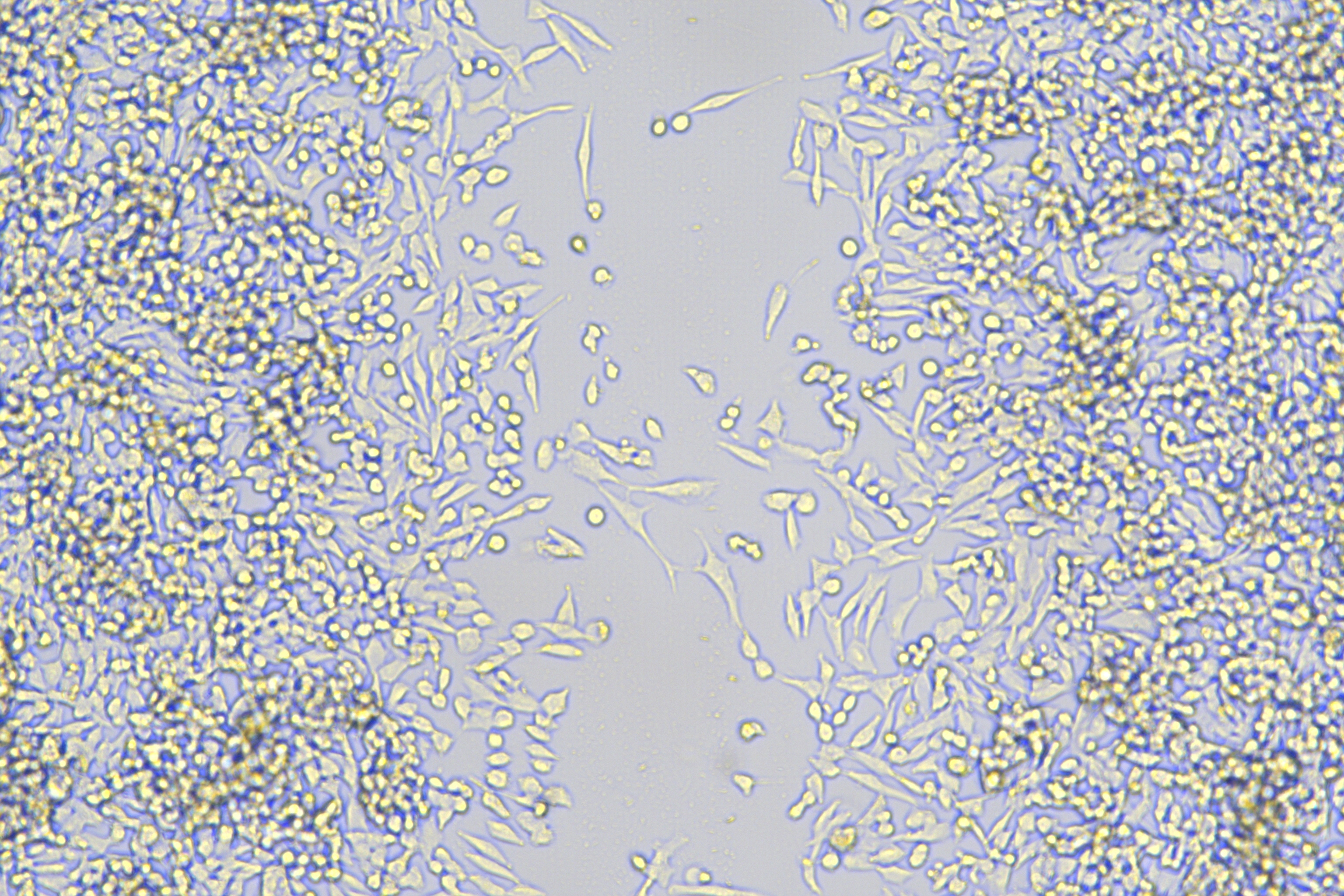

Supplement: Multimedia component 1 [file mmc1.zip › the raw data/Figure 4A/Figure 4A wound healing/24 h/OV-NC.jpg]

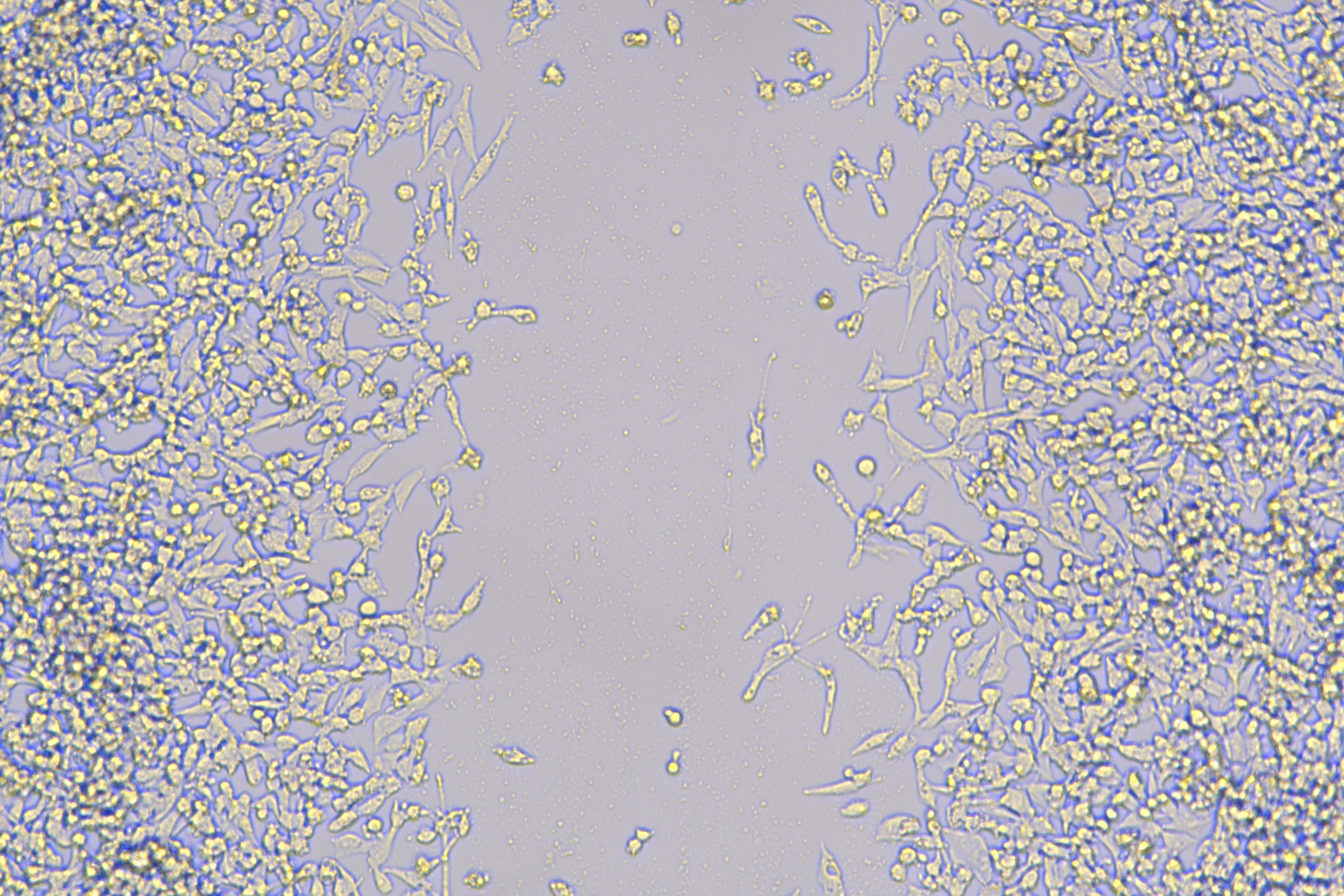

Supplement: Multimedia component 1 [file mmc1.zip › the raw data/Figure 4A/Figure 4A wound healing/24 h/Si-MYBL2.jpg]

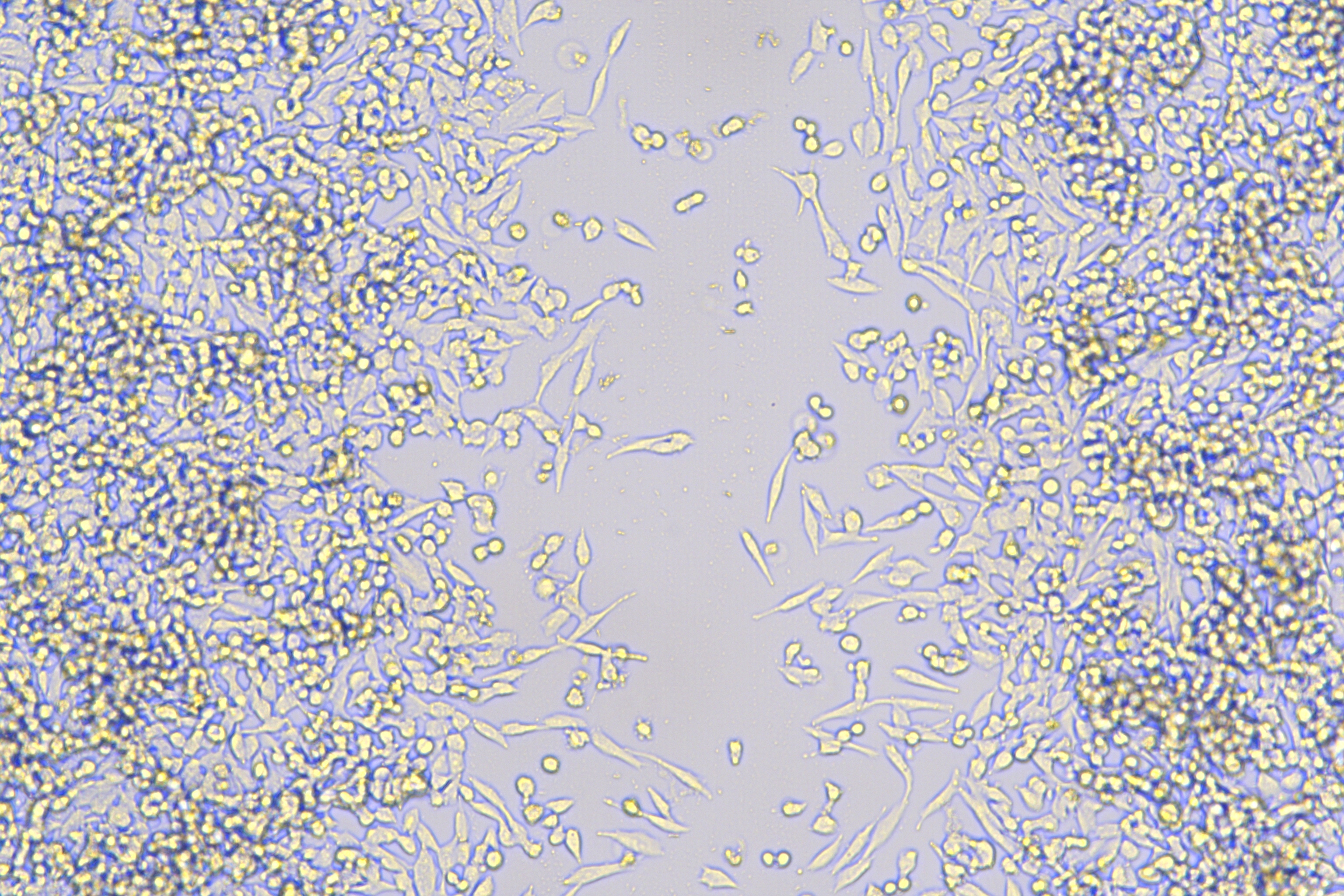

Supplement: Multimedia component 1 [file mmc1.zip › the raw data/Figure 4A/Figure 4A wound healing/24 h/Si-NC.jpg]

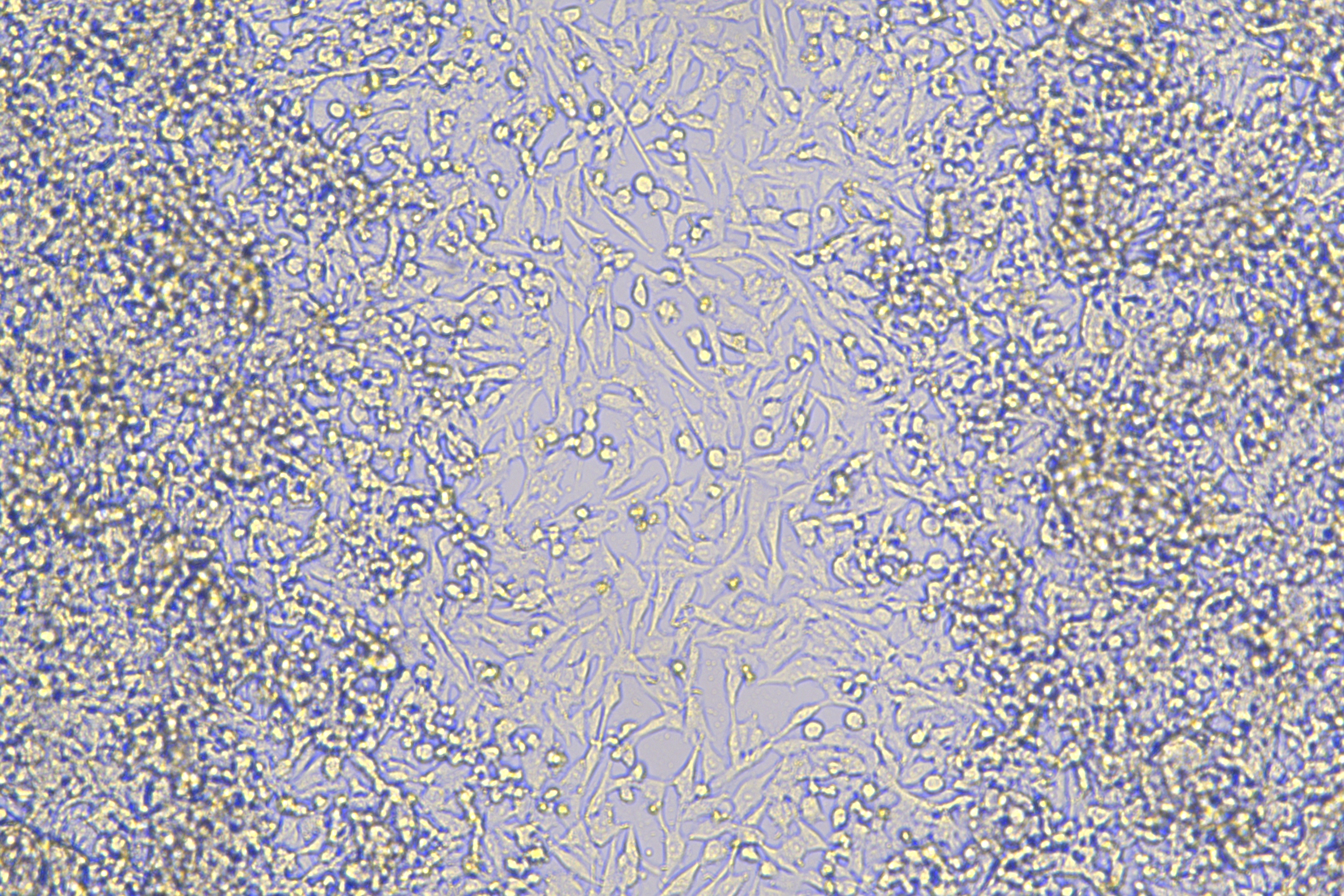

Supplement: Multimedia component 1 [file mmc1.zip › the raw data/Figure 4A/Figure 4A wound healing/48 h/OV-MYBL2.jpg]

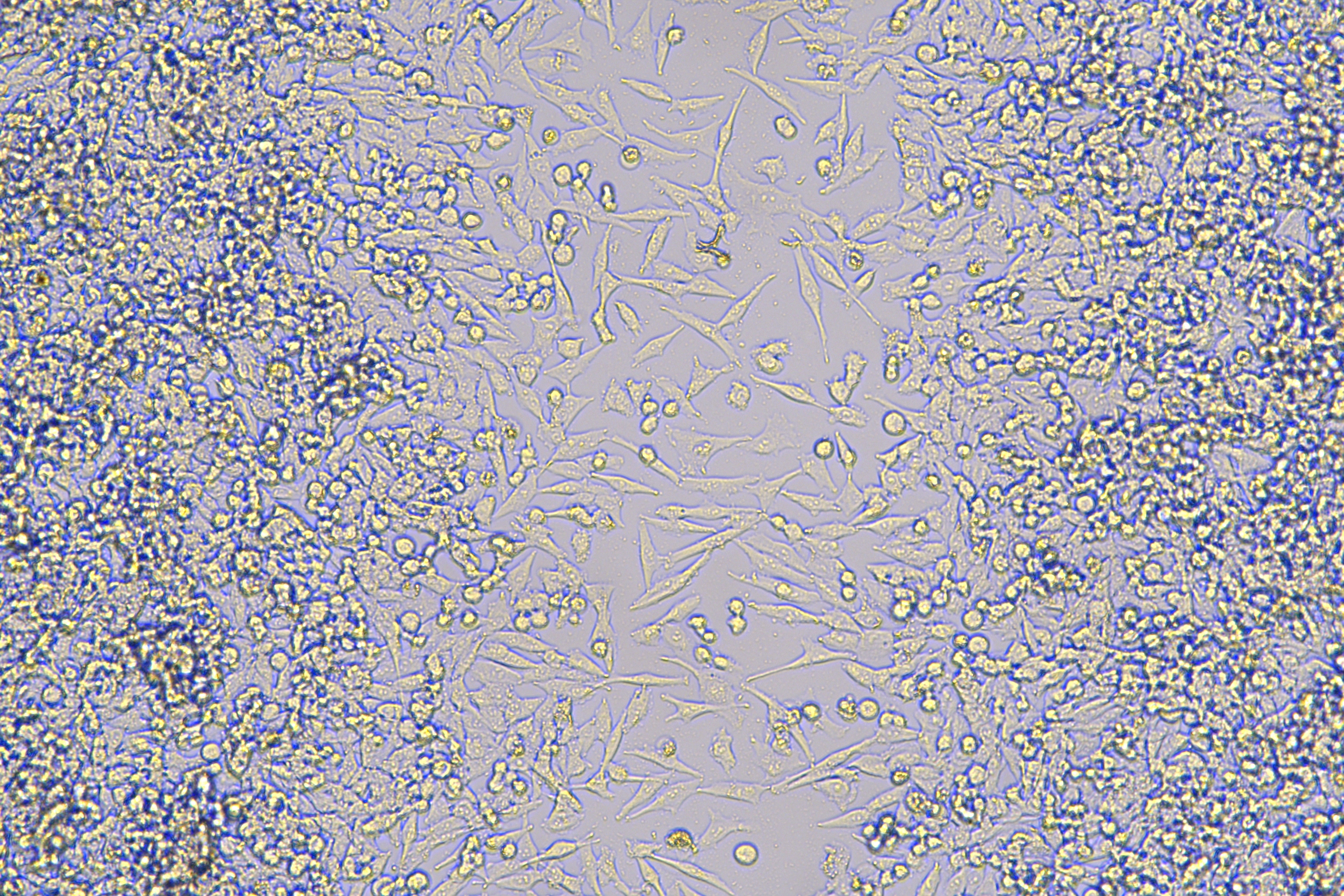

Supplement: Multimedia component 1 [file mmc1.zip › the raw data/Figure 4A/Figure 4A wound healing/48 h/OV-NC.jpg]

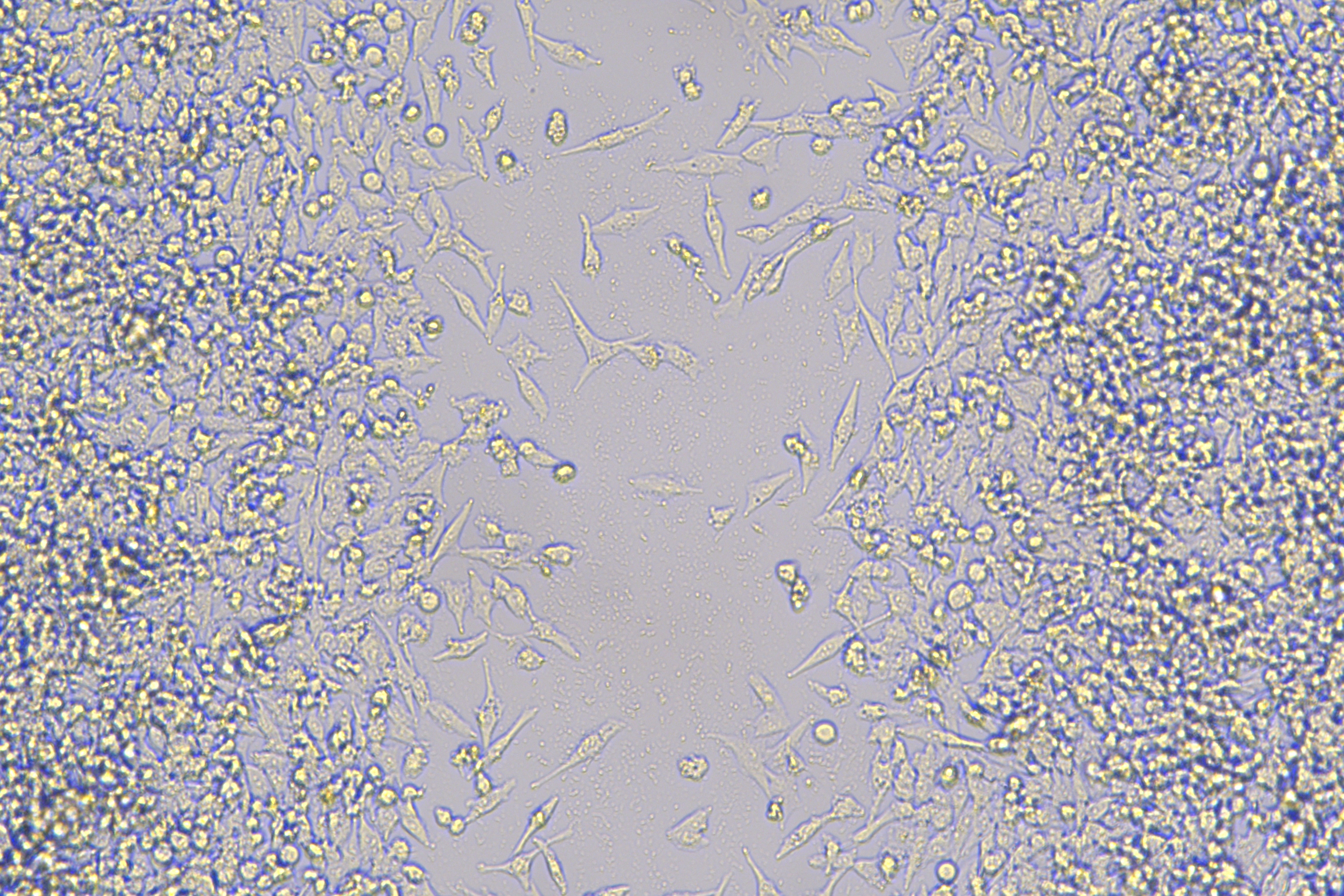

Supplement: Multimedia component 1 [file mmc1.zip › the raw data/Figure 4A/Figure 4A wound healing/48 h/Si-MYBL2.jpg]

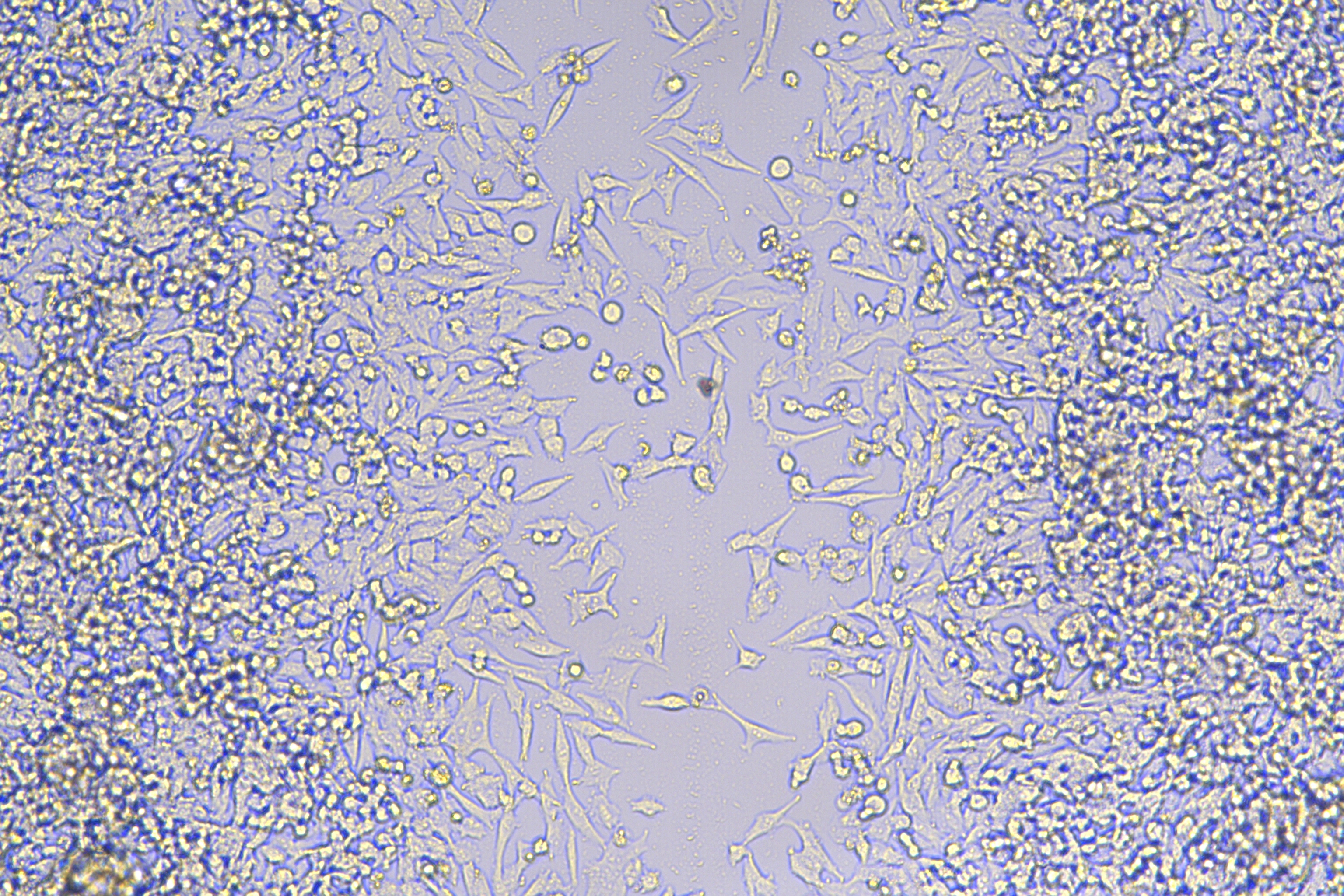

Supplement: Multimedia component 1 [file mmc1.zip › the raw data/Figure 4A/Figure 4A wound healing/48 h/Si-NC.jpg]

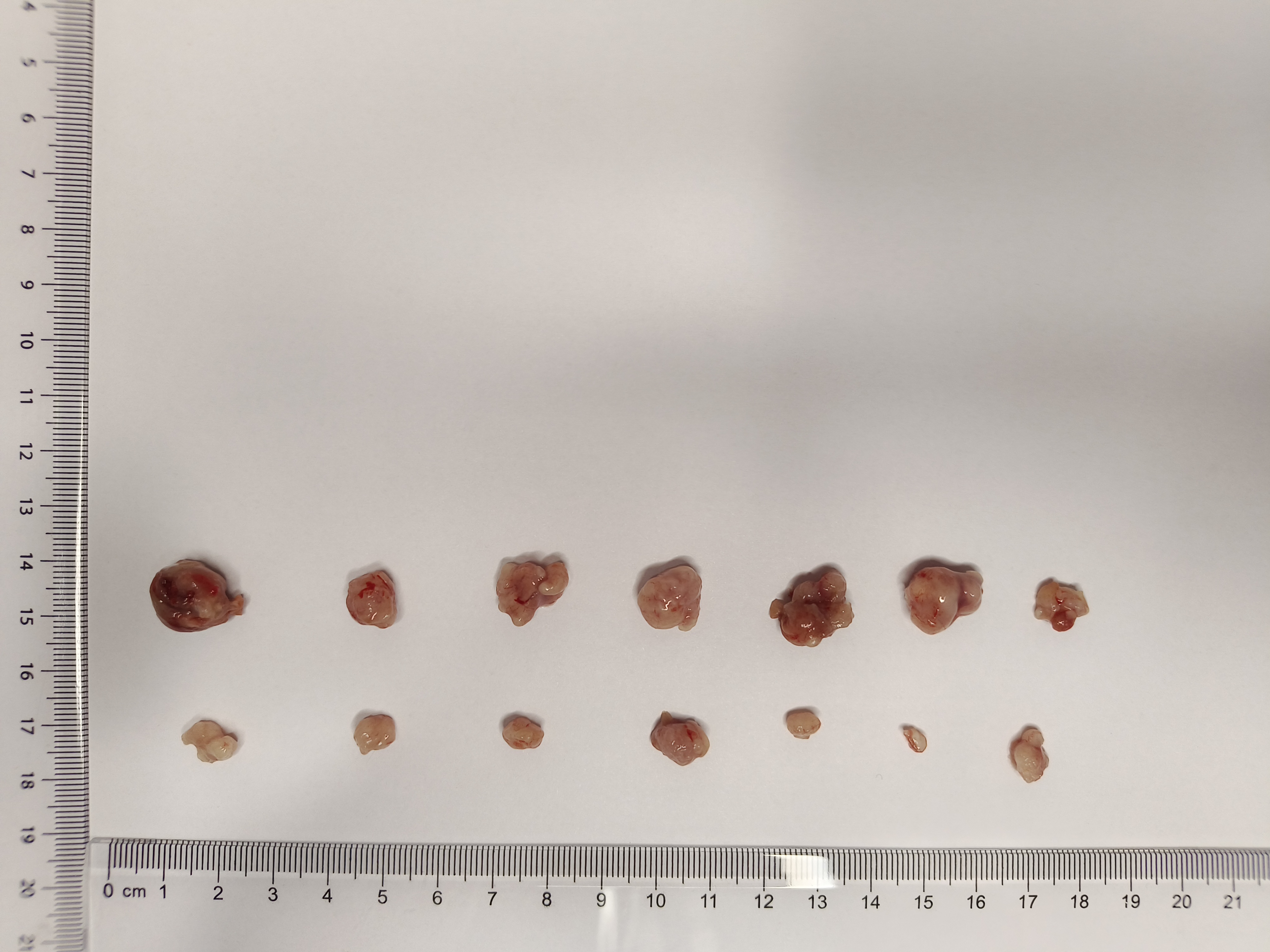

Supplement: Multimedia component 1 [file mmc1.zip › the raw data/Figure 5A/Tumor sizes in BALBC nude mice.jpg]

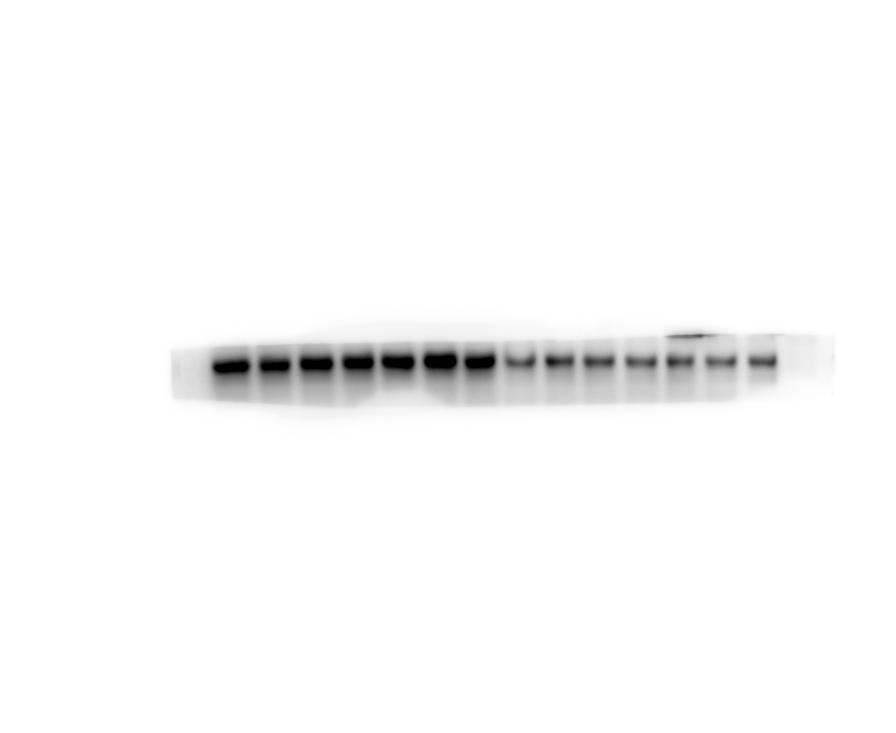

Supplement: Multimedia component 1 [file mmc1.zip › the raw data/Figure 5D/CACD8.jpg]

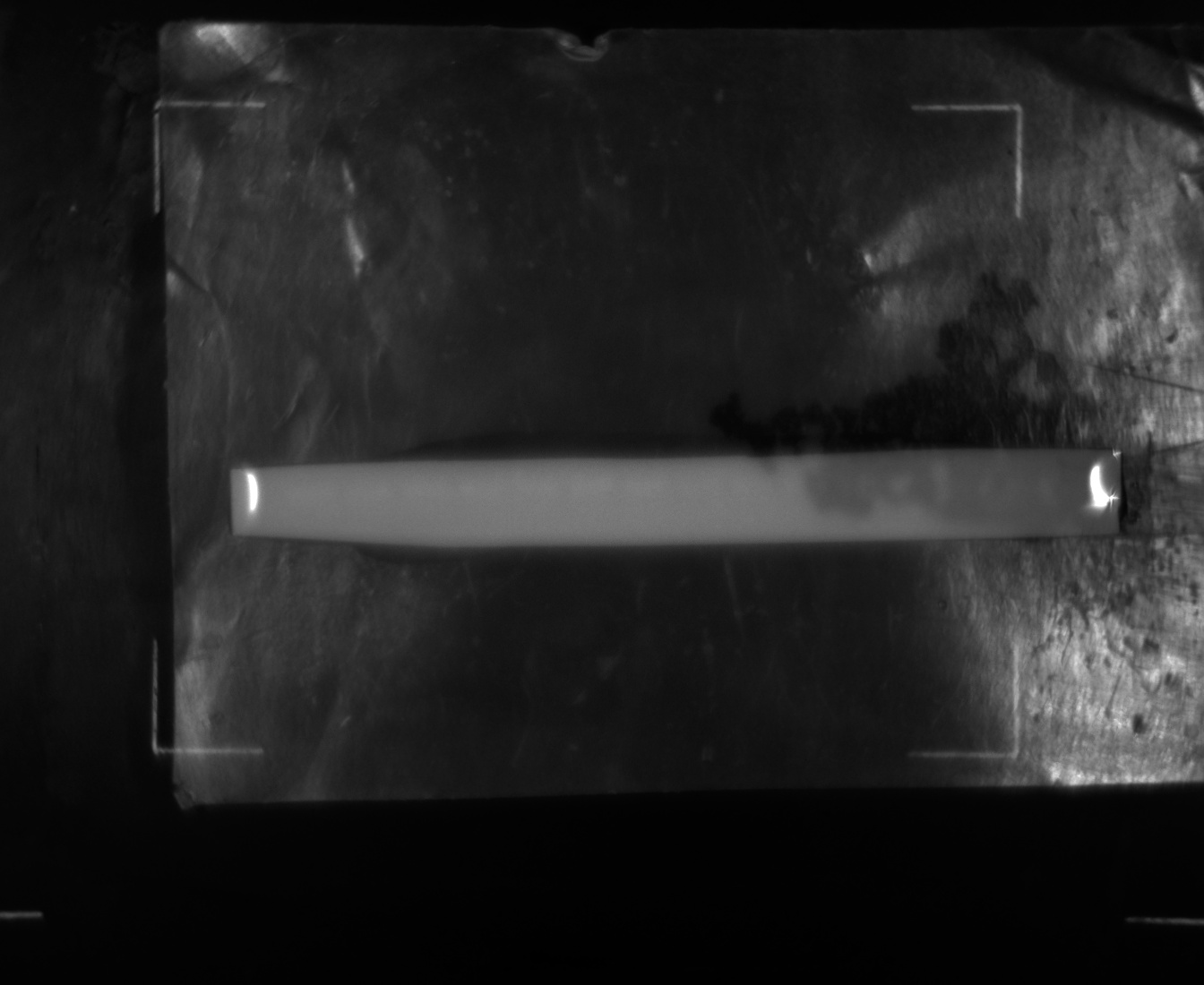

Supplement: Multimedia component 1 [file mmc1.zip › the raw data/Figure 5D/CDCA8-bright-field image of PVDF membrane.jpg]

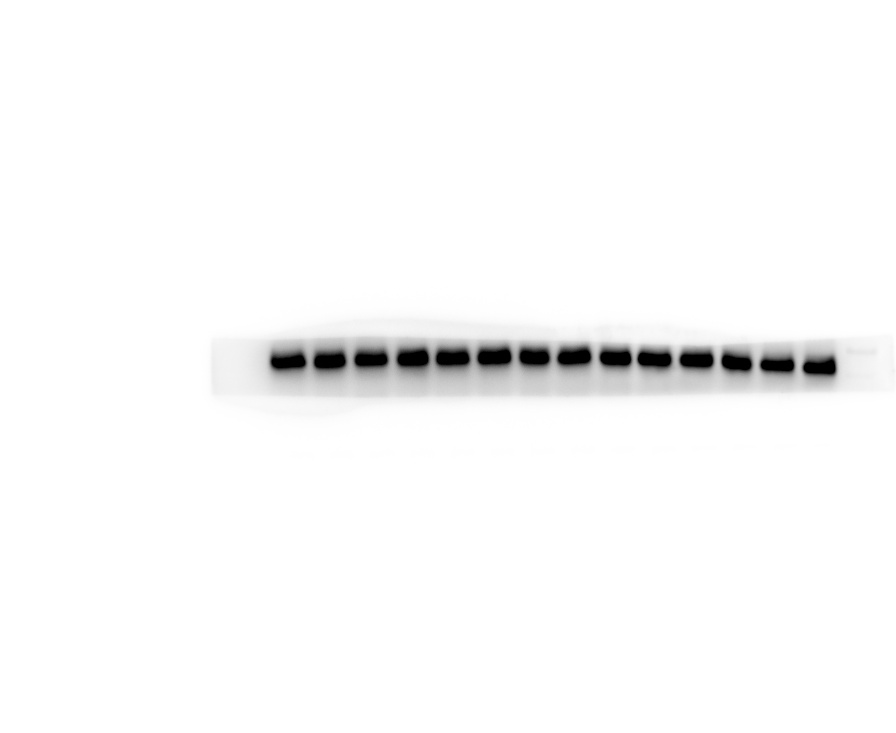

Supplement: Multimedia component 1 [file mmc1.zip › the raw data/Figure 5D/GAPDH.jpg]

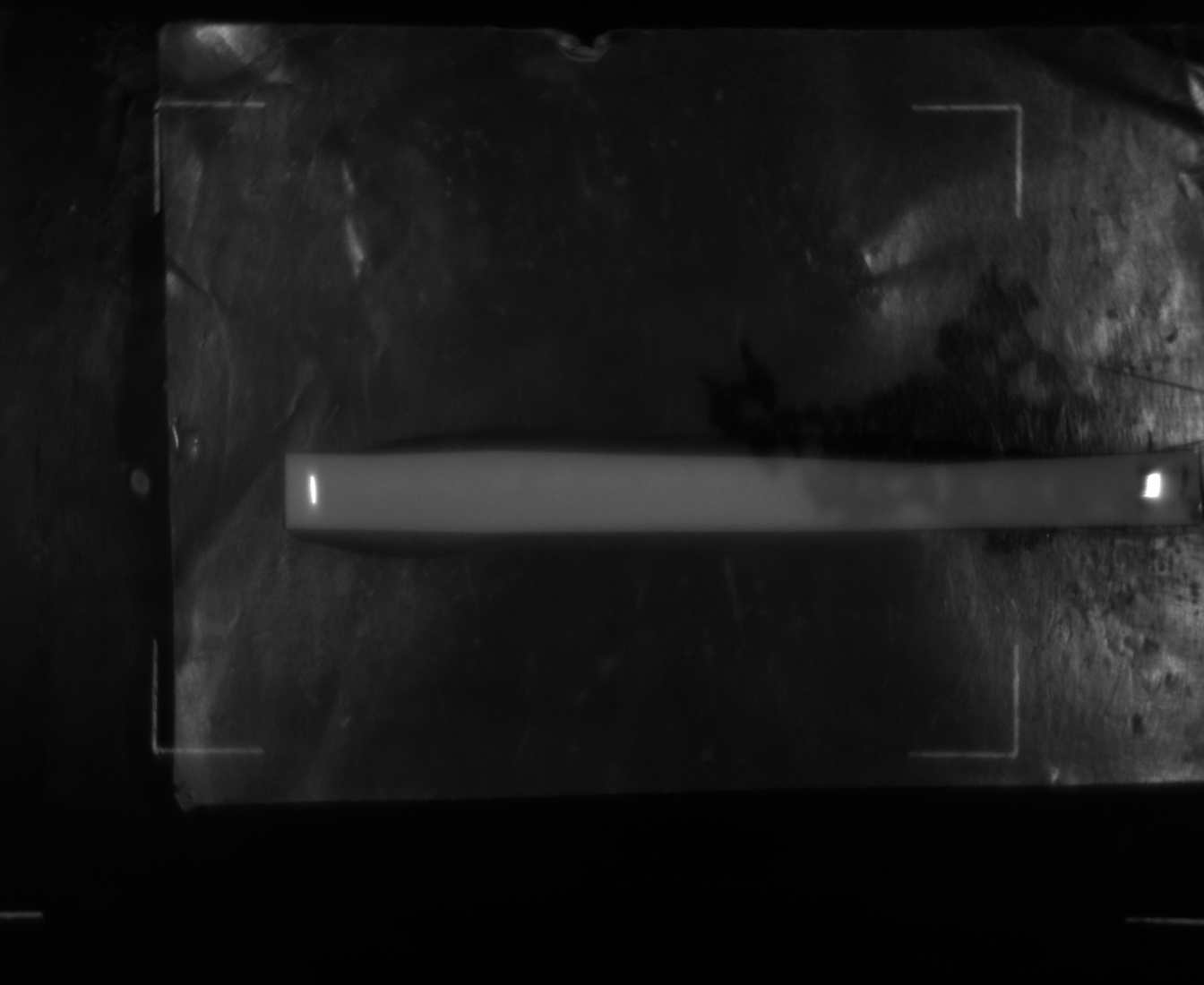

Supplement: Multimedia component 1 [file mmc1.zip › the raw data/Figure 5D/GAPDH-bright-field image of PVDF membrane.jpg]

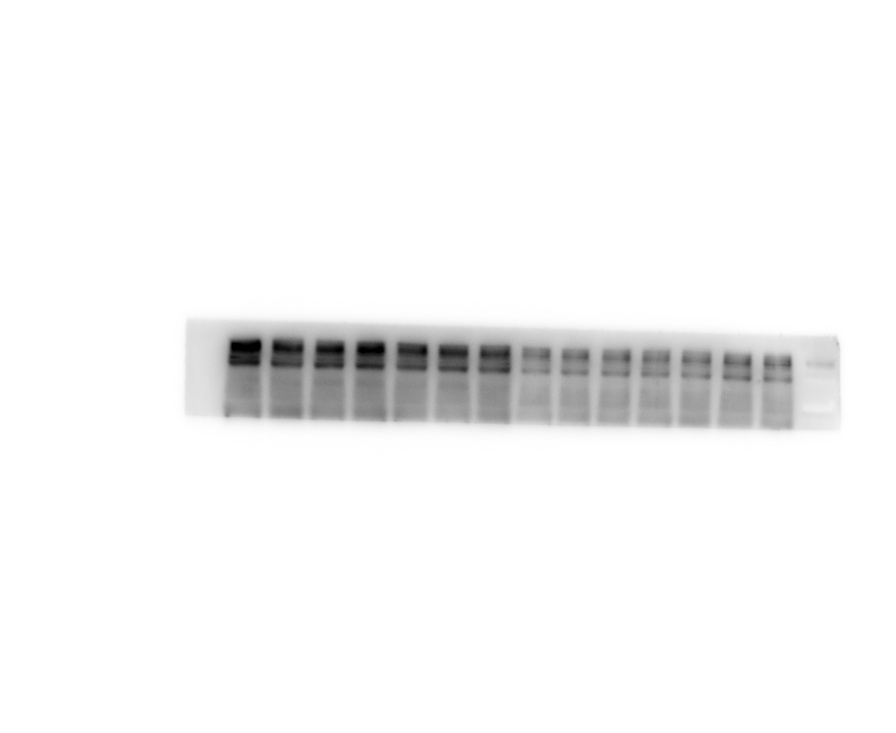

Supplement: Multimedia component 1 [file mmc1.zip › the raw data/Figure 5D/MYBL2.jpg]

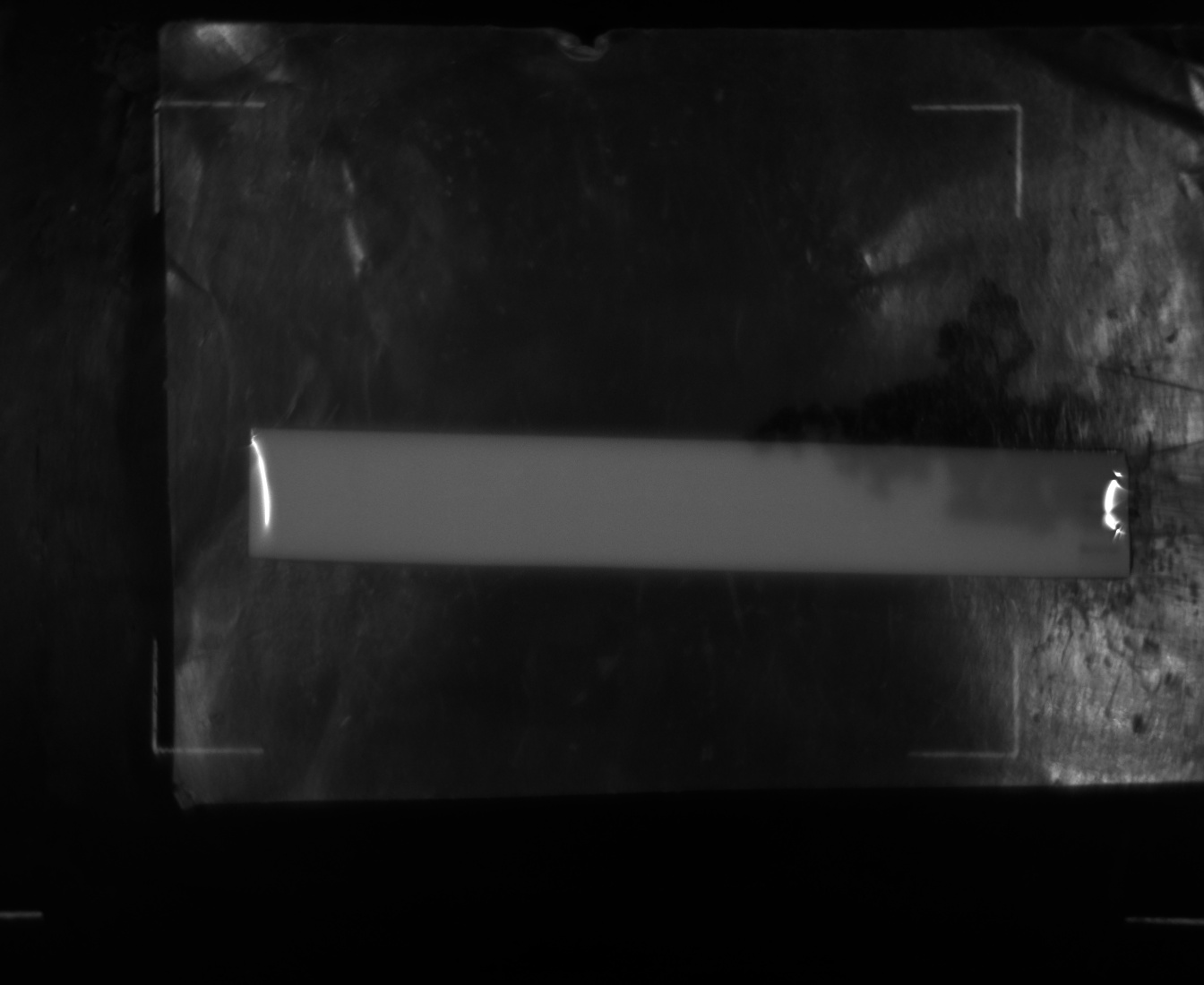

Supplement: Multimedia component 1 [file mmc1.zip › the raw data/Figure 5D/MYBL2-bright-field image of PVDF membrane.jpg]

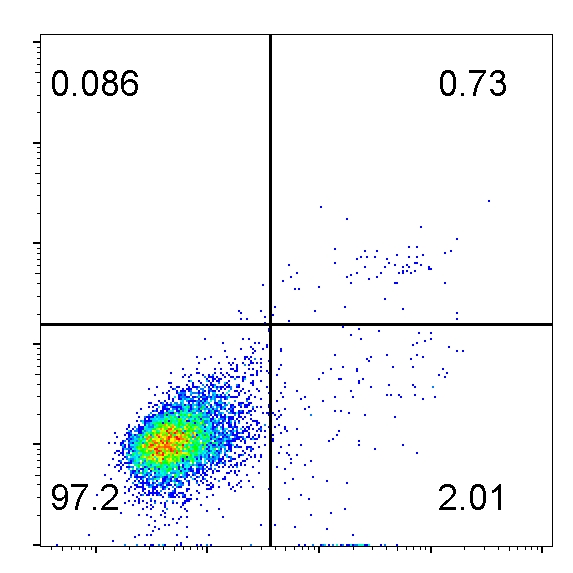

Supplement: Multimedia component 1 [file mmc1.zip › the raw data/Figure 6A/Figure 6A FCM/OV-CDCA8.jpg]

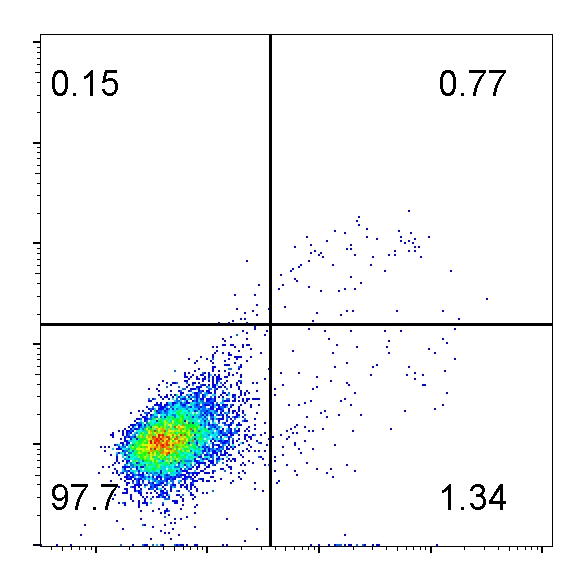

Supplement: Multimedia component 1 [file mmc1.zip › the raw data/Figure 6A/Figure 6A FCM/OV-MYBL2.jpg]

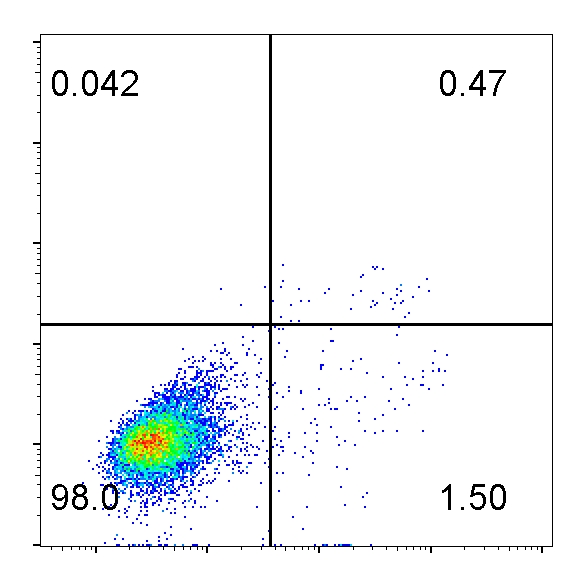

Supplement: Multimedia component 1 [file mmc1.zip › the raw data/Figure 6A/Figure 6A FCM/OV-MYBL2+OV-CDCA8.jpg]

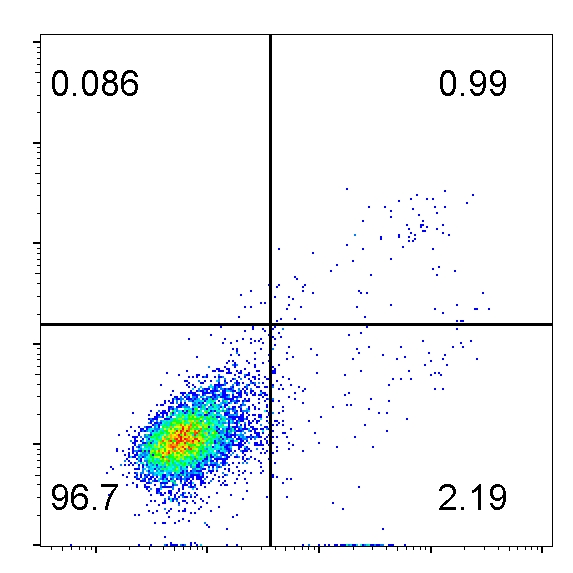

Supplement: Multimedia component 1 [file mmc1.zip › the raw data/Figure 6A/Figure 6A FCM/OV-NC.jpg]

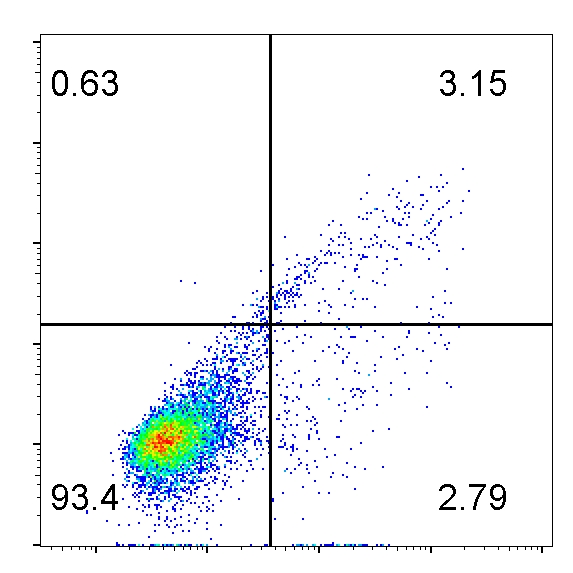

Supplement: Multimedia component 1 [file mmc1.zip › the raw data/Figure 6A/Figure 6A FCM/Si-CDCA8.jpg]

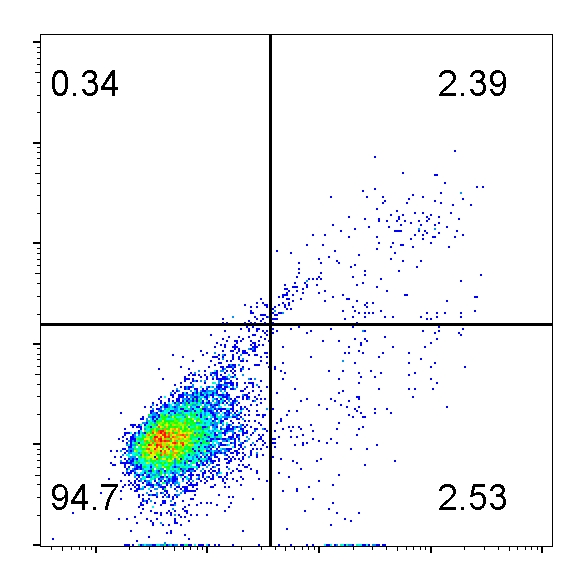

Supplement: Multimedia component 1 [file mmc1.zip › the raw data/Figure 6A/Figure 6A FCM/Si-MYBL2.jpg]

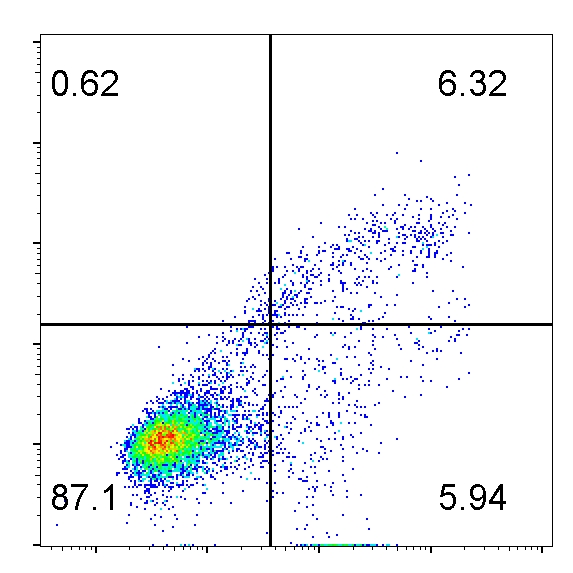

Supplement: Multimedia component 1 [file mmc1.zip › the raw data/Figure 6A/Figure 6A FCM/Si-MYBL2+Si-CDCA8.jpg]

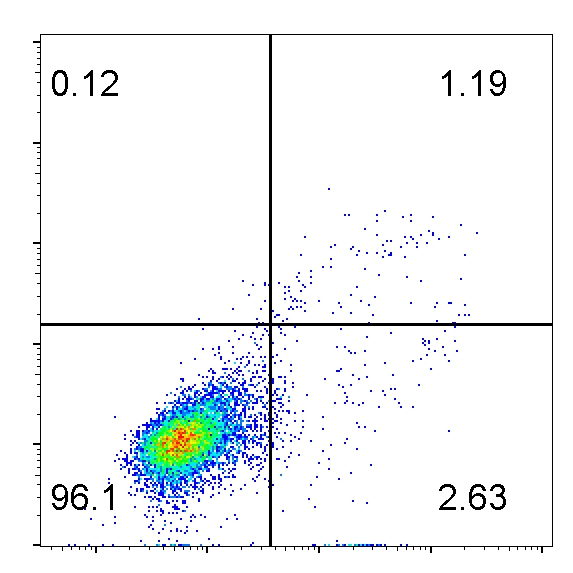

Supplement: Multimedia component 1 [file mmc1.zip › the raw data/Figure 6A/Figure 6A FCM/Si-NC.jpg]

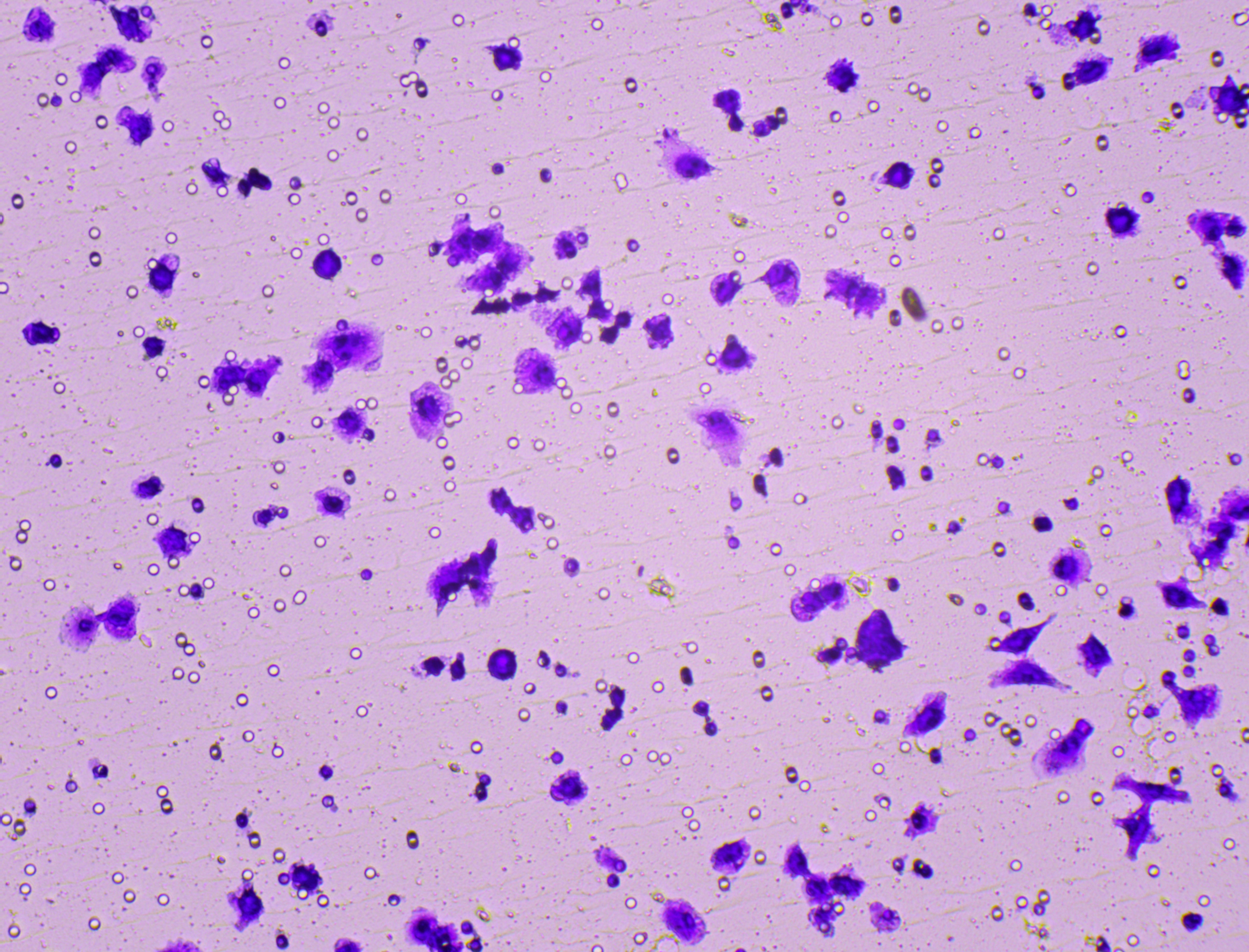

Supplement: Multimedia component 1 [file mmc1.zip › the raw data/Figure 6A/Figure 6A invasion/OV-CDCA8.jpg]

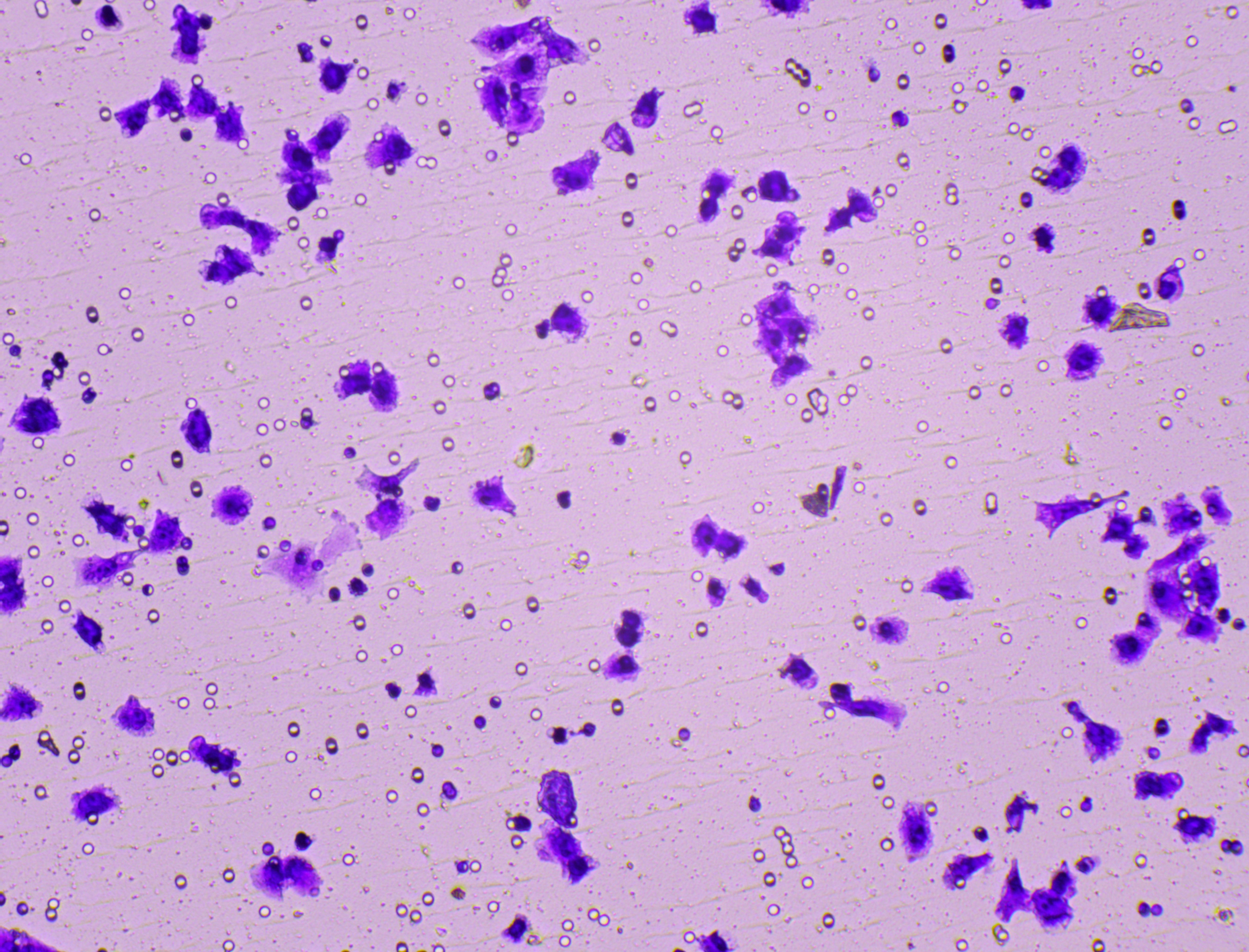

Supplement: Multimedia component 1 [file mmc1.zip › the raw data/Figure 6A/Figure 6A invasion/OV-MYBL2.jpg]

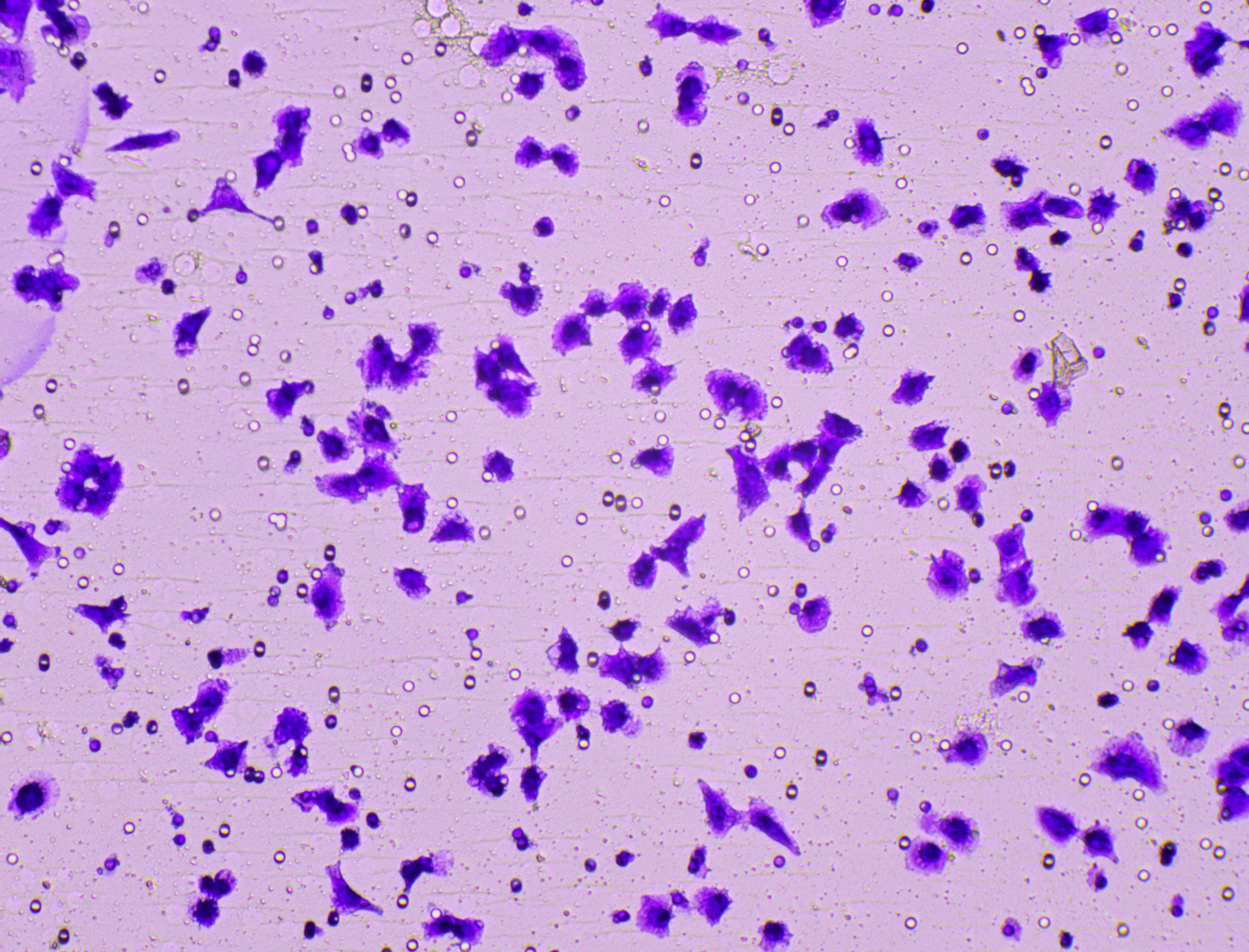

Supplement: Multimedia component 1 [file mmc1.zip › the raw data/Figure 6A/Figure 6A invasion/OV-MYBL2+OV-CDCA8.jpg]

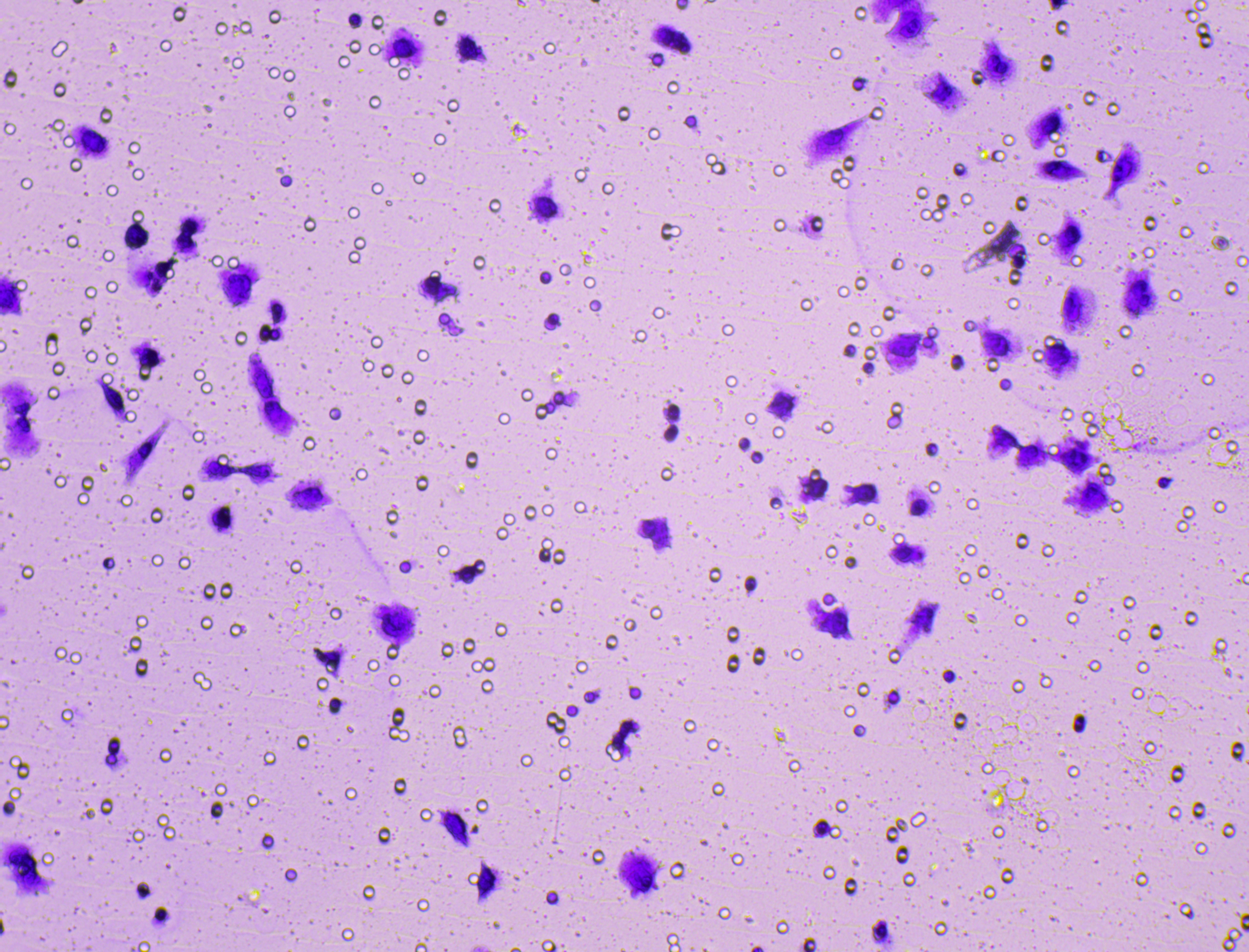

Supplement: Multimedia component 1 [file mmc1.zip › the raw data/Figure 6A/Figure 6A invasion/OV-NC.jpg]

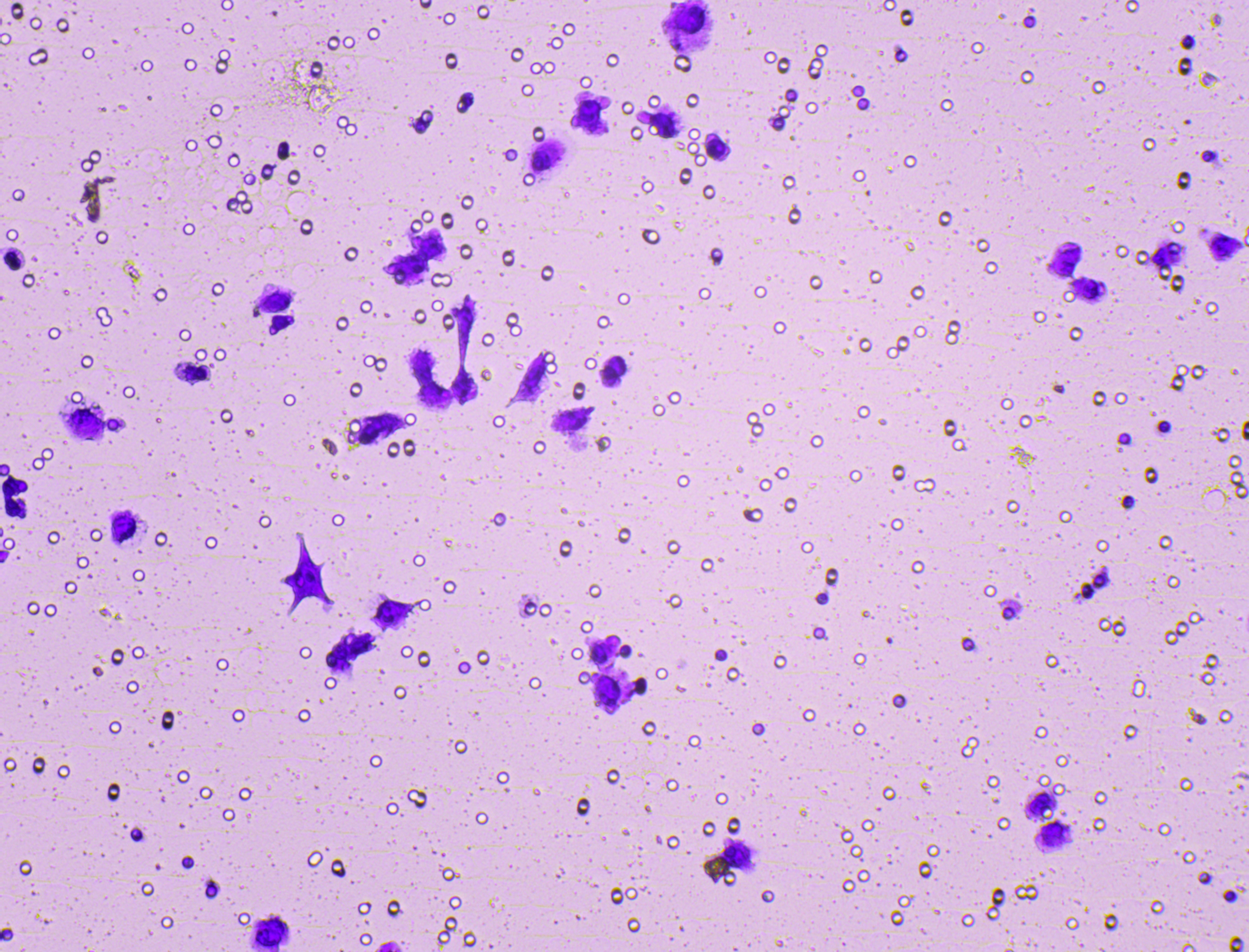

Supplement: Multimedia component 1 [file mmc1.zip › the raw data/Figure 6A/Figure 6A invasion/Si-CDCA8.jpg]

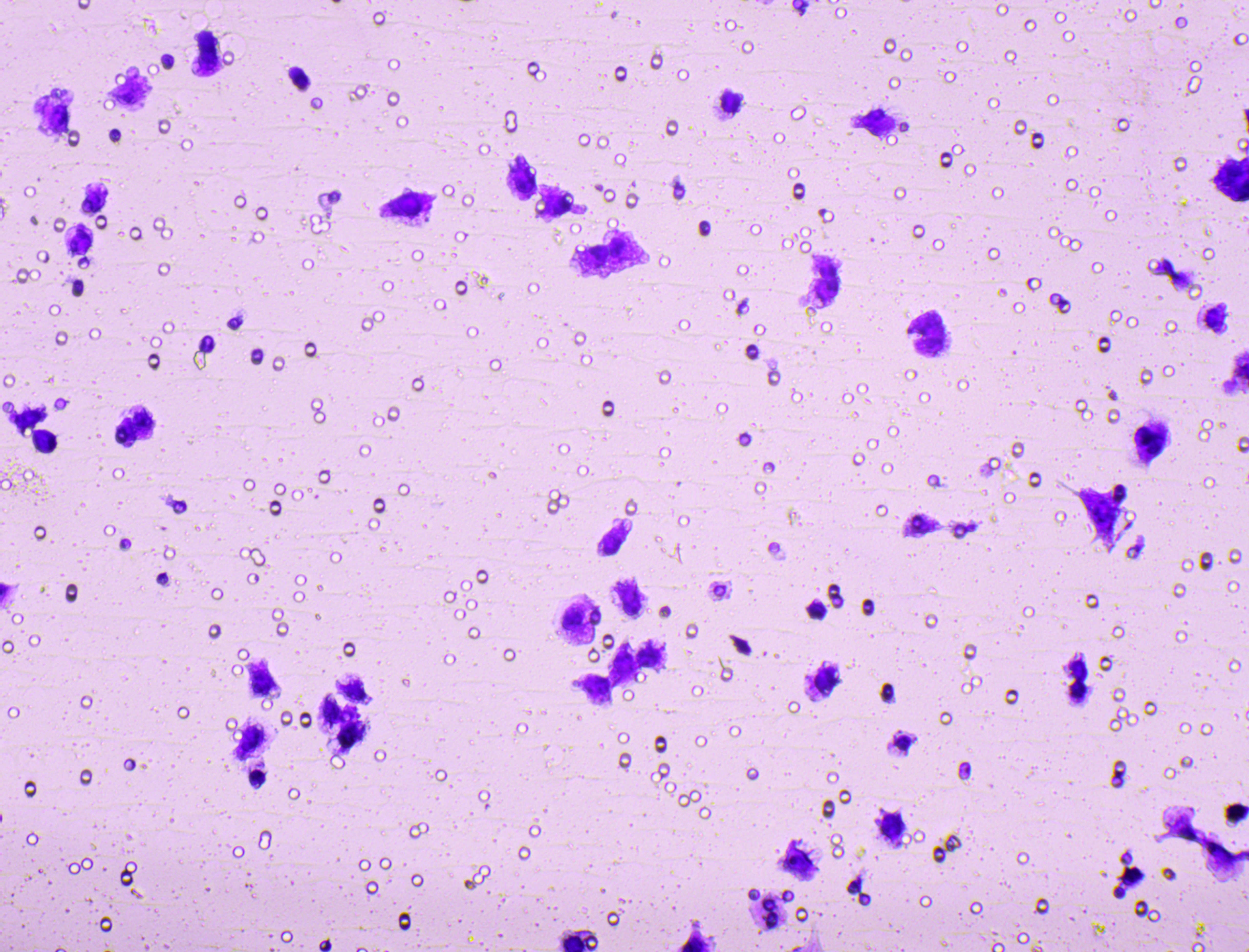

Supplement: Multimedia component 1 [file mmc1.zip › the raw data/Figure 6A/Figure 6A invasion/Si-MYBL2.jpg]

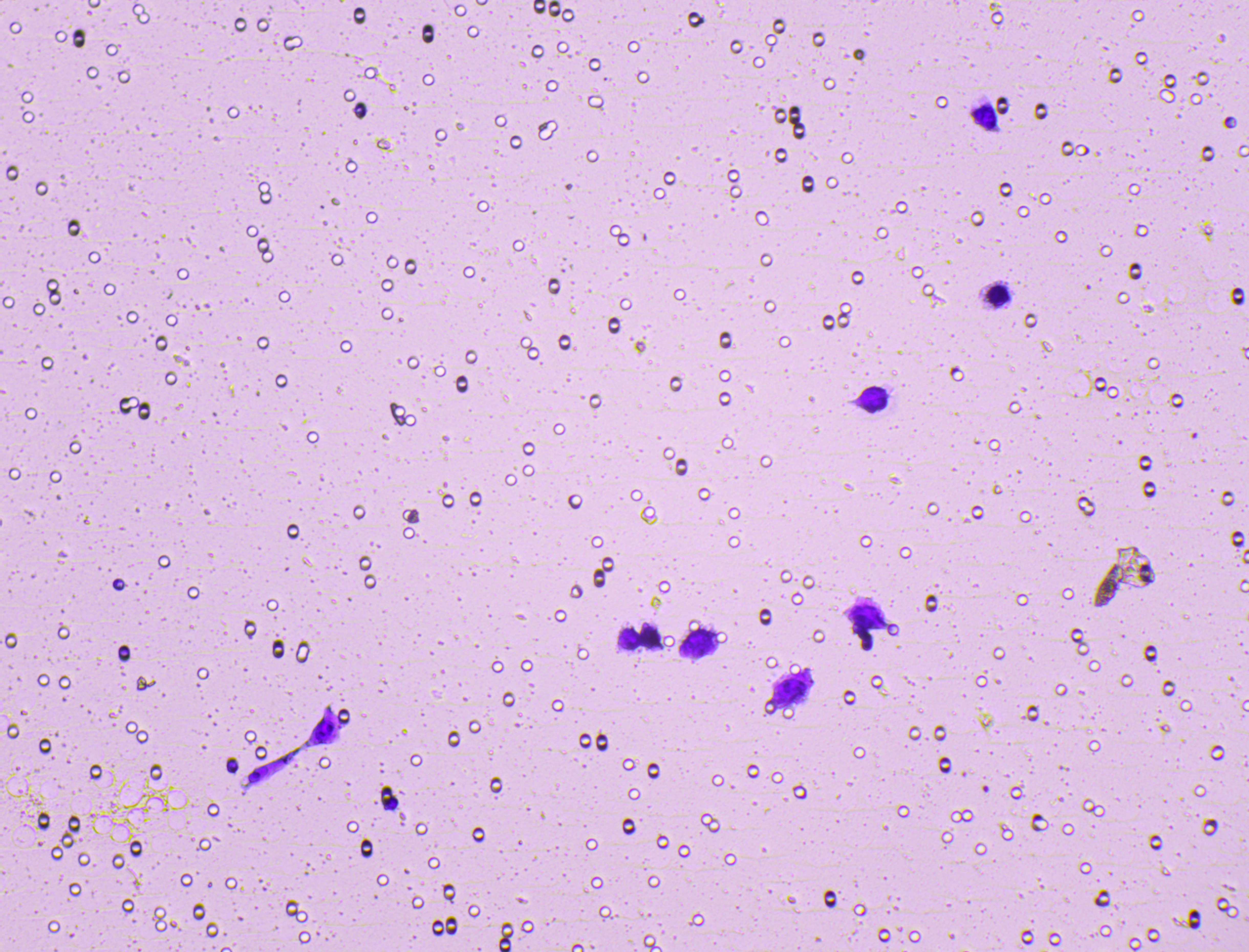

Supplement: Multimedia component 1 [file mmc1.zip › the raw data/Figure 6A/Figure 6A invasion/Si-MYBL2+si-CDCA8.jpg]

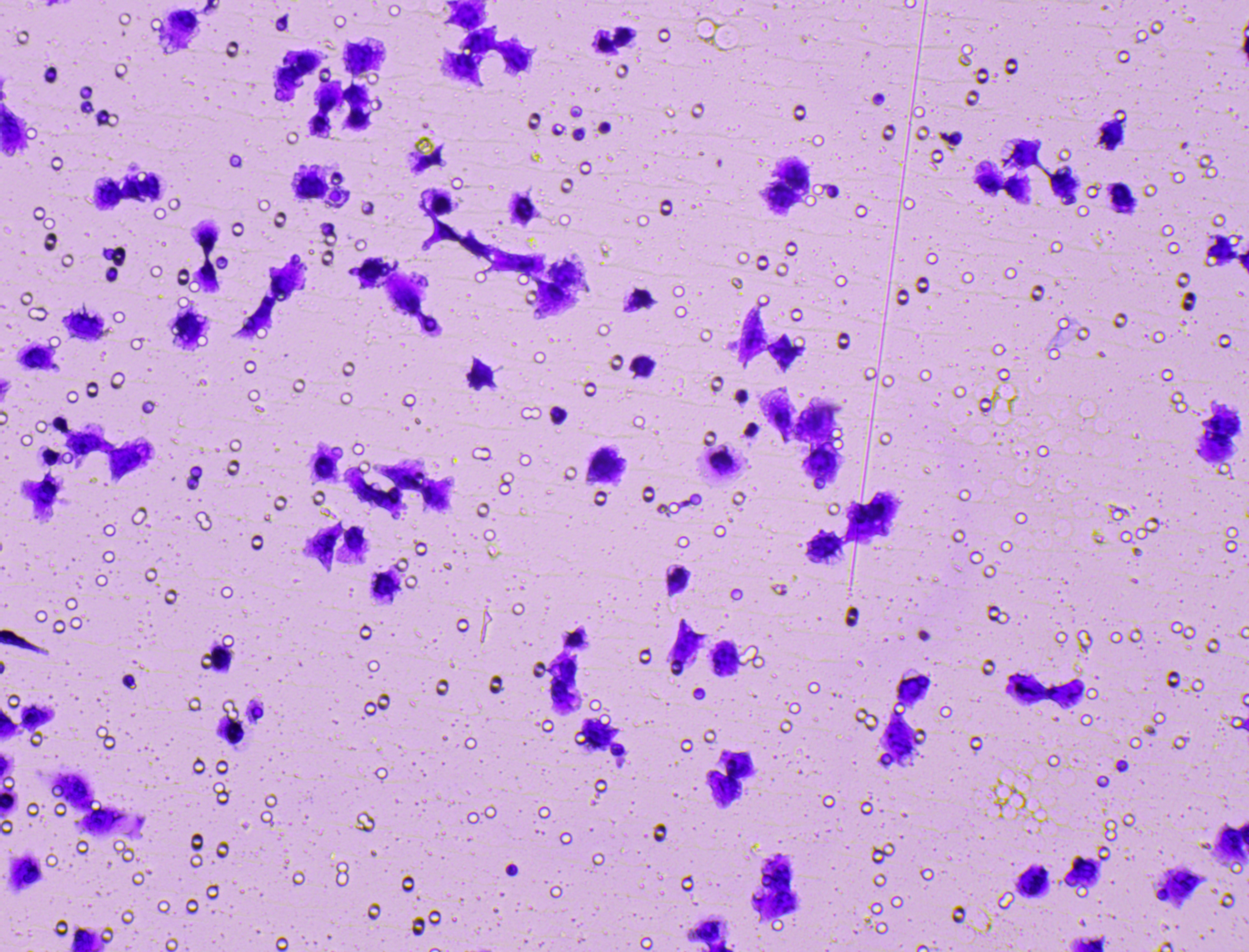

Supplement: Multimedia component 1 [file mmc1.zip › the raw data/Figure 6A/Figure 6A invasion/Si-NC.jpg]

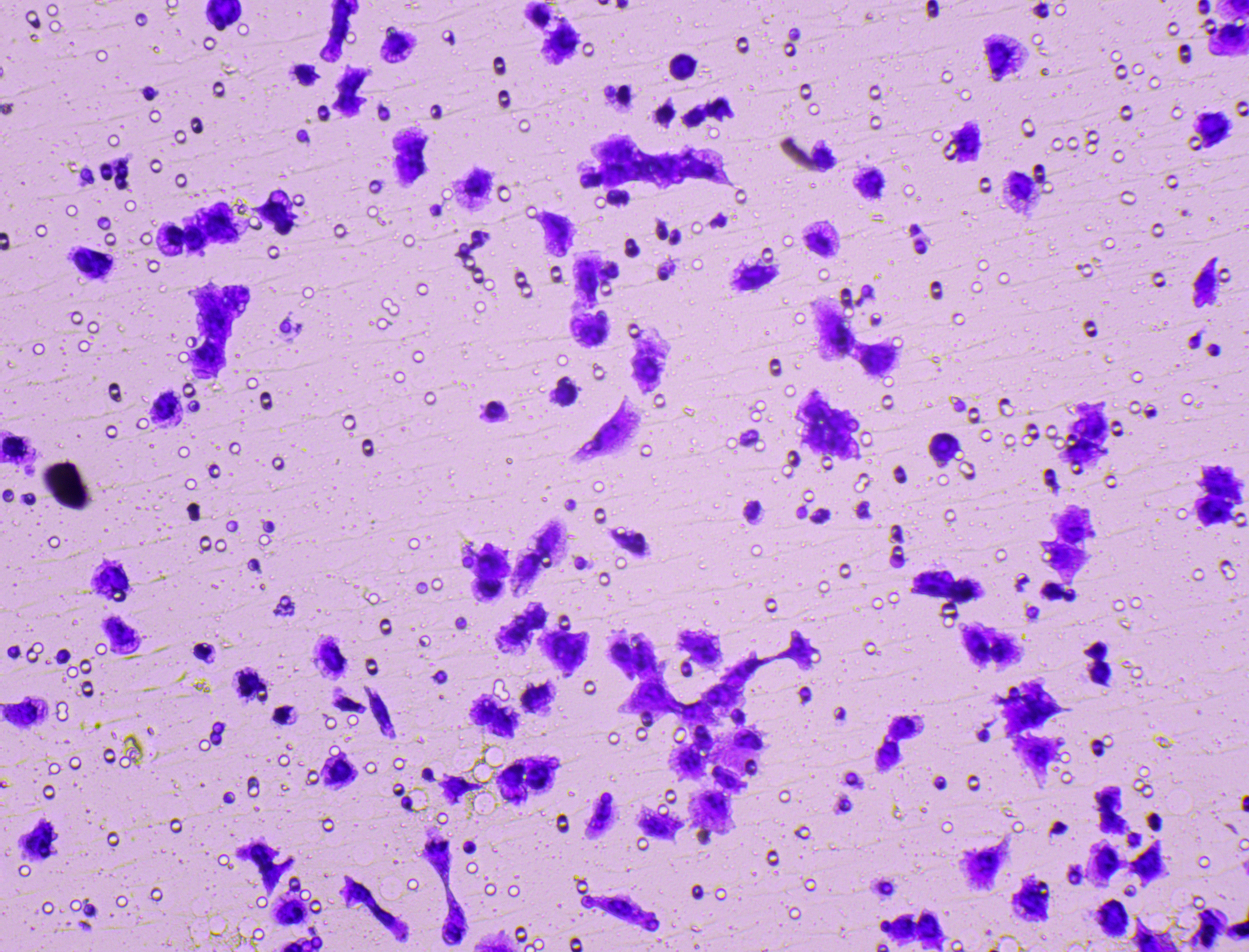

Supplement: Multimedia component 1 [file mmc1.zip › the raw data/Figure 6A/Figure 6A migiration/OV-CDCA8.jpg]

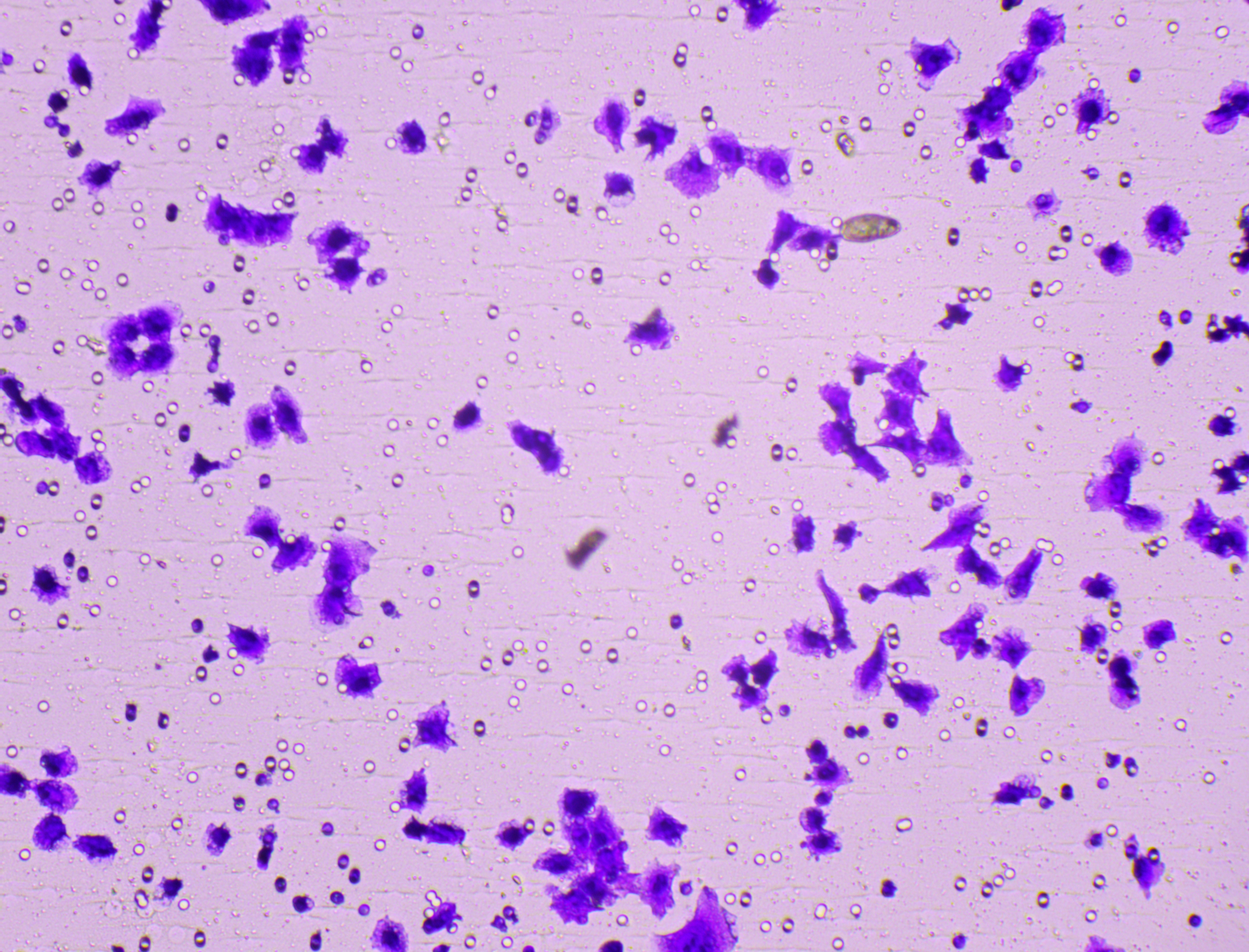

Supplement: Multimedia component 1 [file mmc1.zip › the raw data/Figure 6A/Figure 6A migiration/OV-MYBL2.jpg]

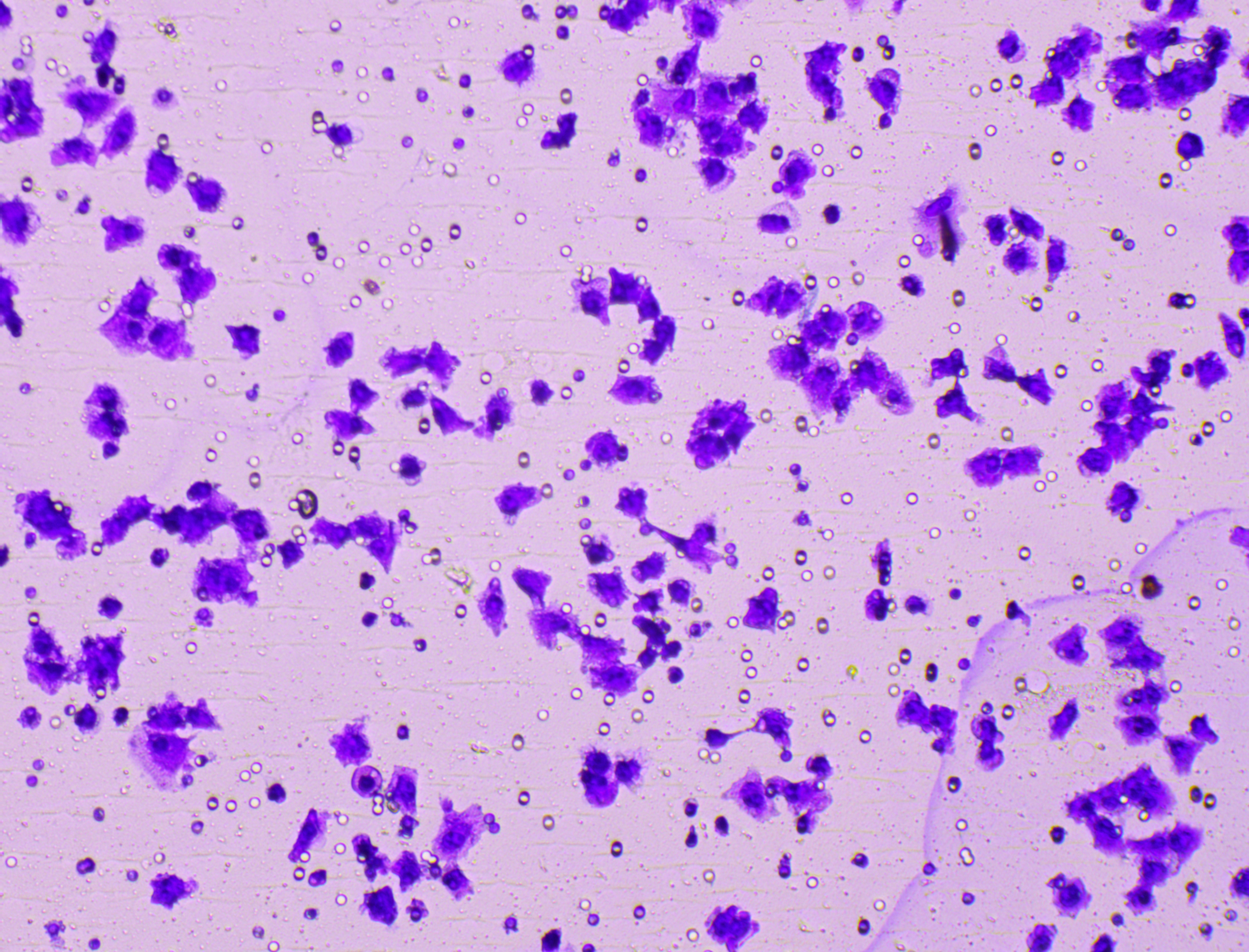

Supplement: Multimedia component 1 [file mmc1.zip › the raw data/Figure 6A/Figure 6A migiration/OV-MYBL2+OV-CDCA8.jpg]

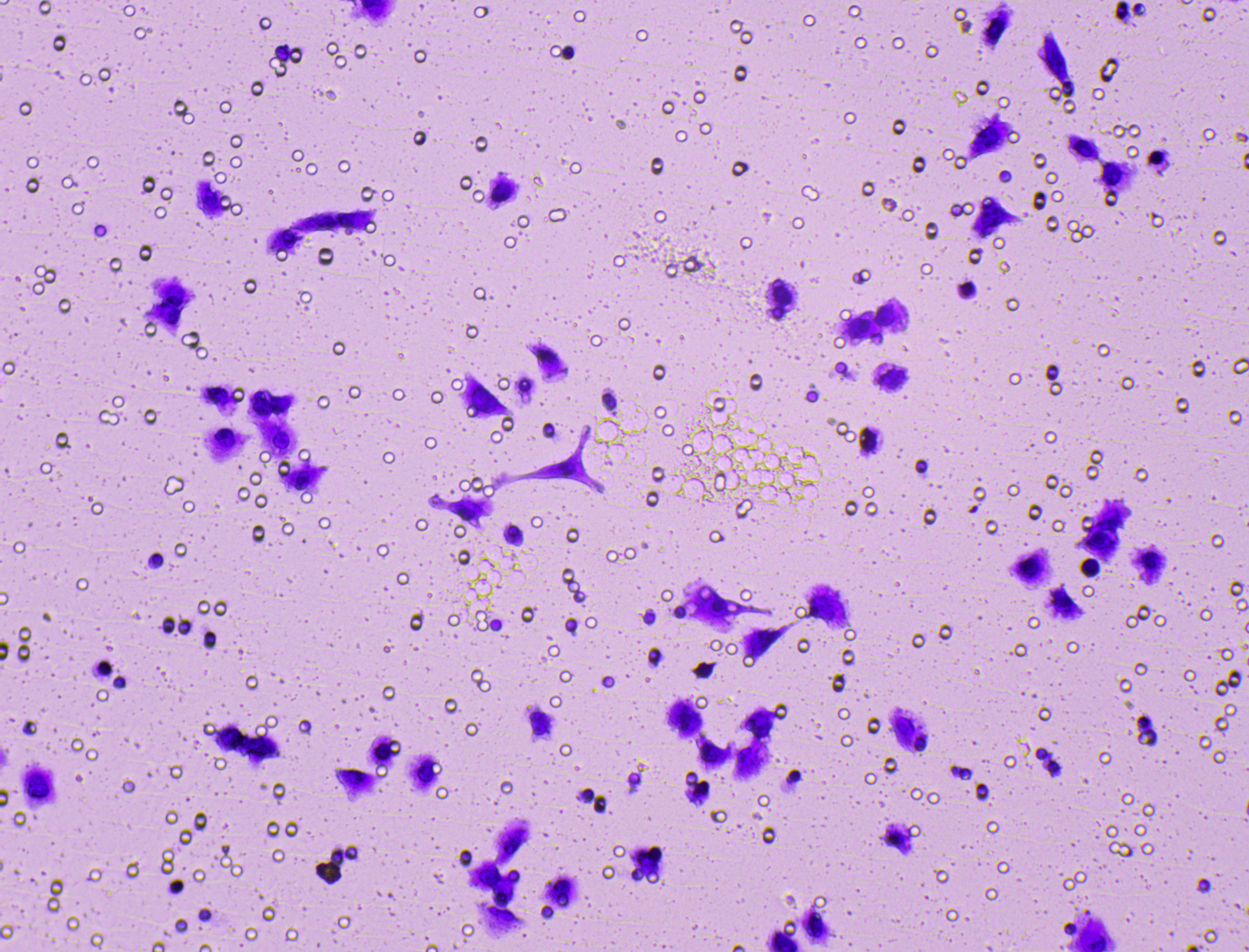

Supplement: Multimedia component 1 [file mmc1.zip › the raw data/Figure 6A/Figure 6A migiration/OV-NC.jpg]

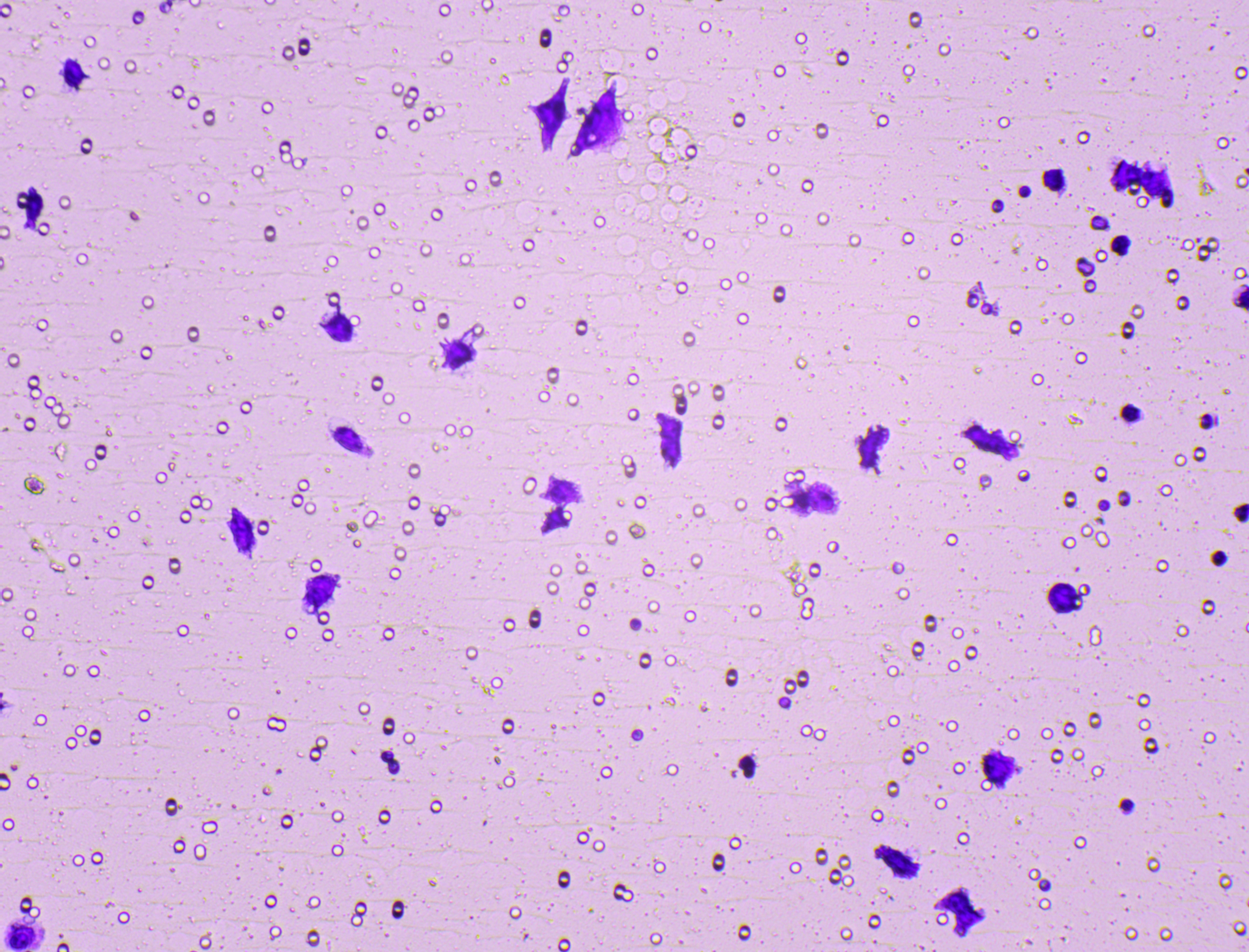

Supplement: Multimedia component 1 [file mmc1.zip › the raw data/Figure 6A/Figure 6A migiration/si-CDCA8.jpg]

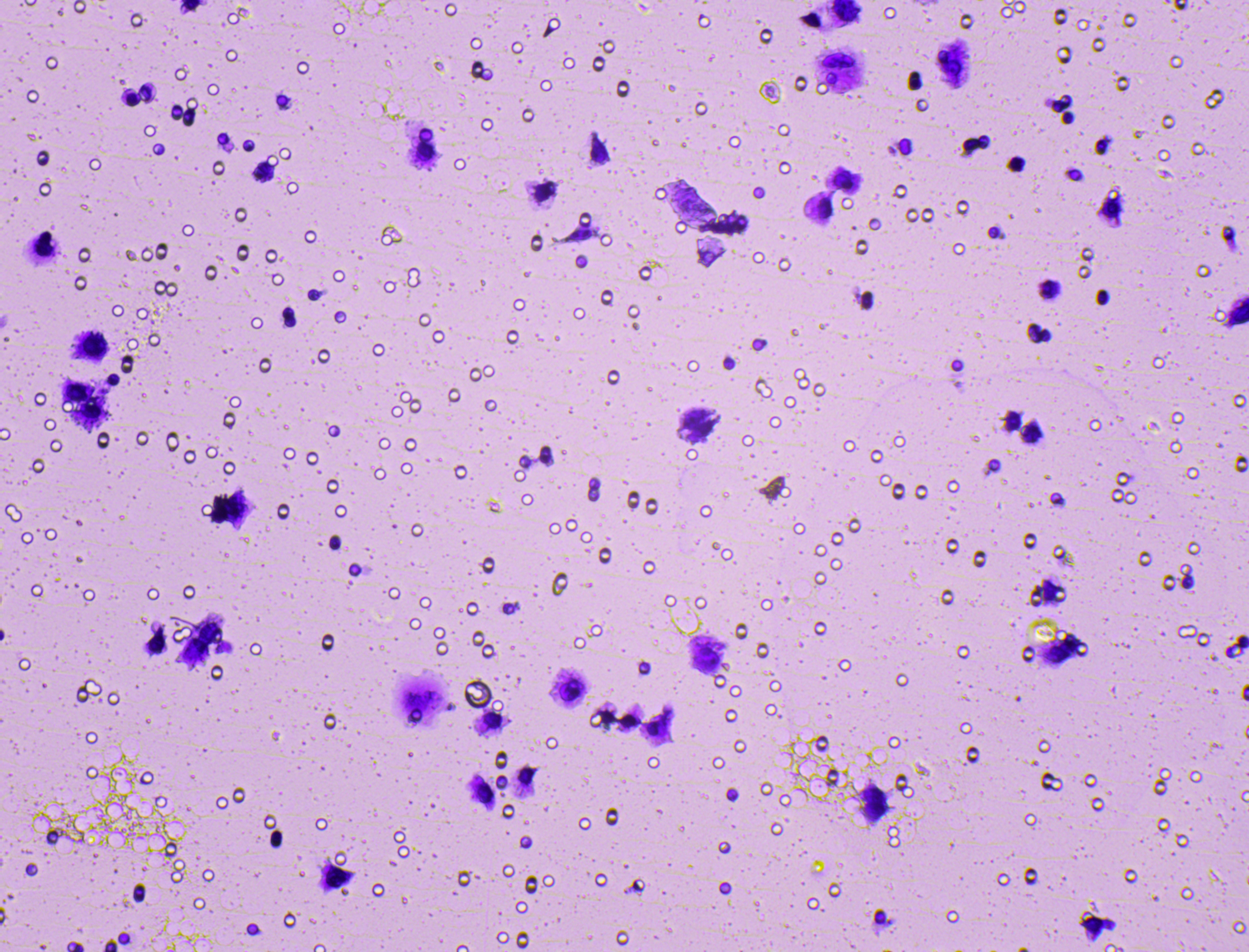

Supplement: Multimedia component 1 [file mmc1.zip › the raw data/Figure 6A/Figure 6A migiration/si-MYBL2.jpg]

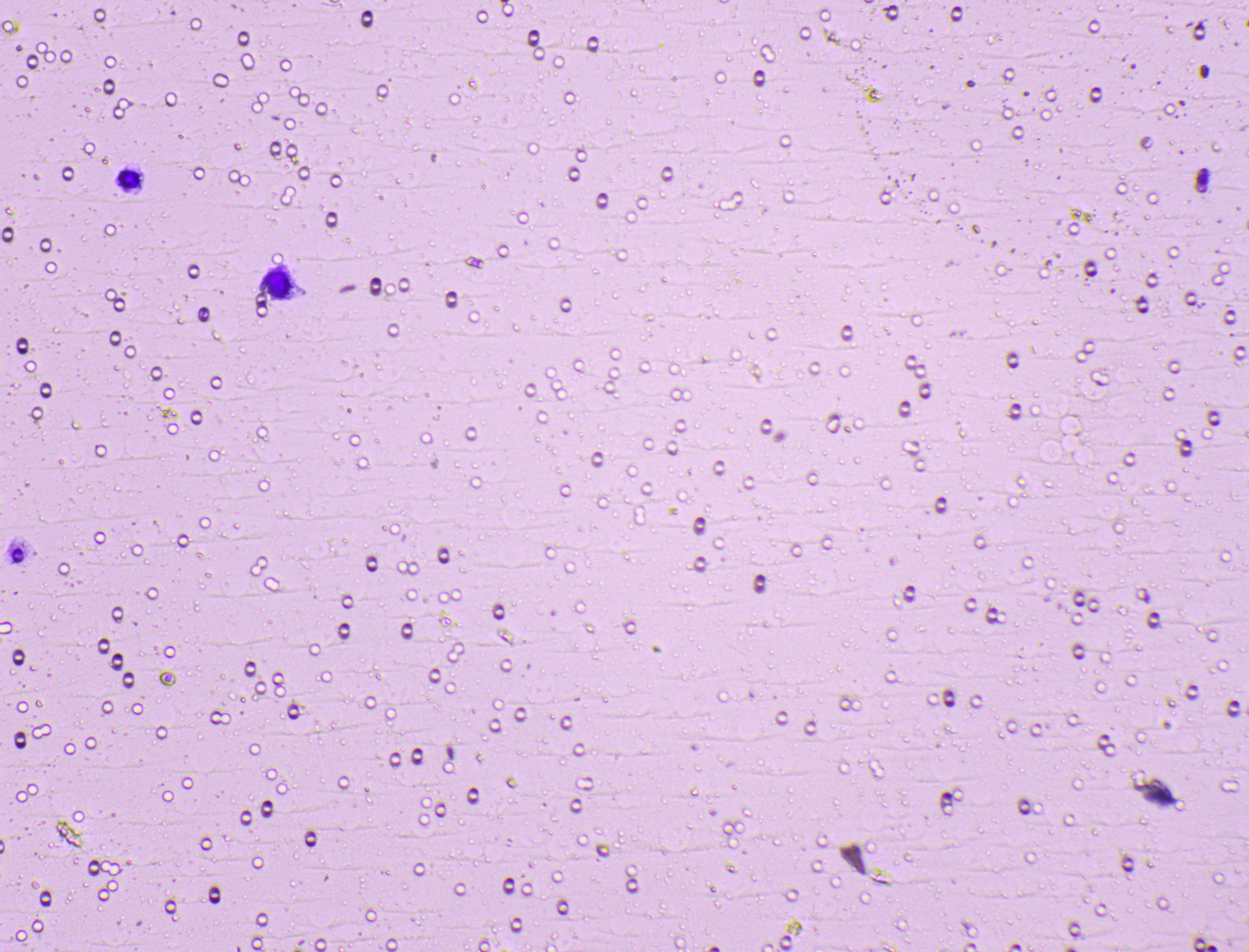

Supplement: Multimedia component 1 [file mmc1.zip › the raw data/Figure 6A/Figure 6A migiration/si-MYBL2+si-CDCA8.jpg]

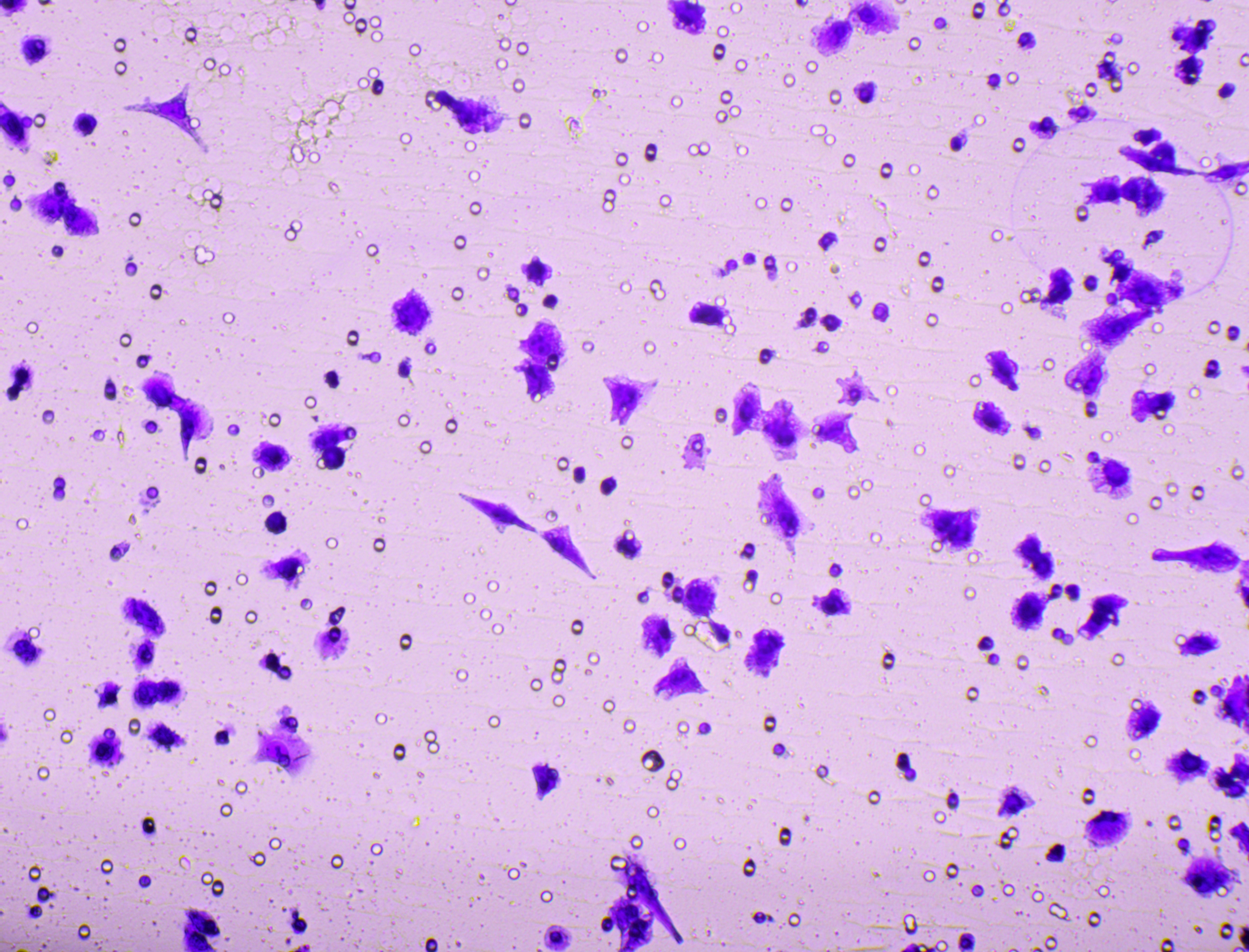

Supplement: Multimedia component 1 [file mmc1.zip › the raw data/Figure 6A/Figure 6A migiration/Si-NC.jpg]

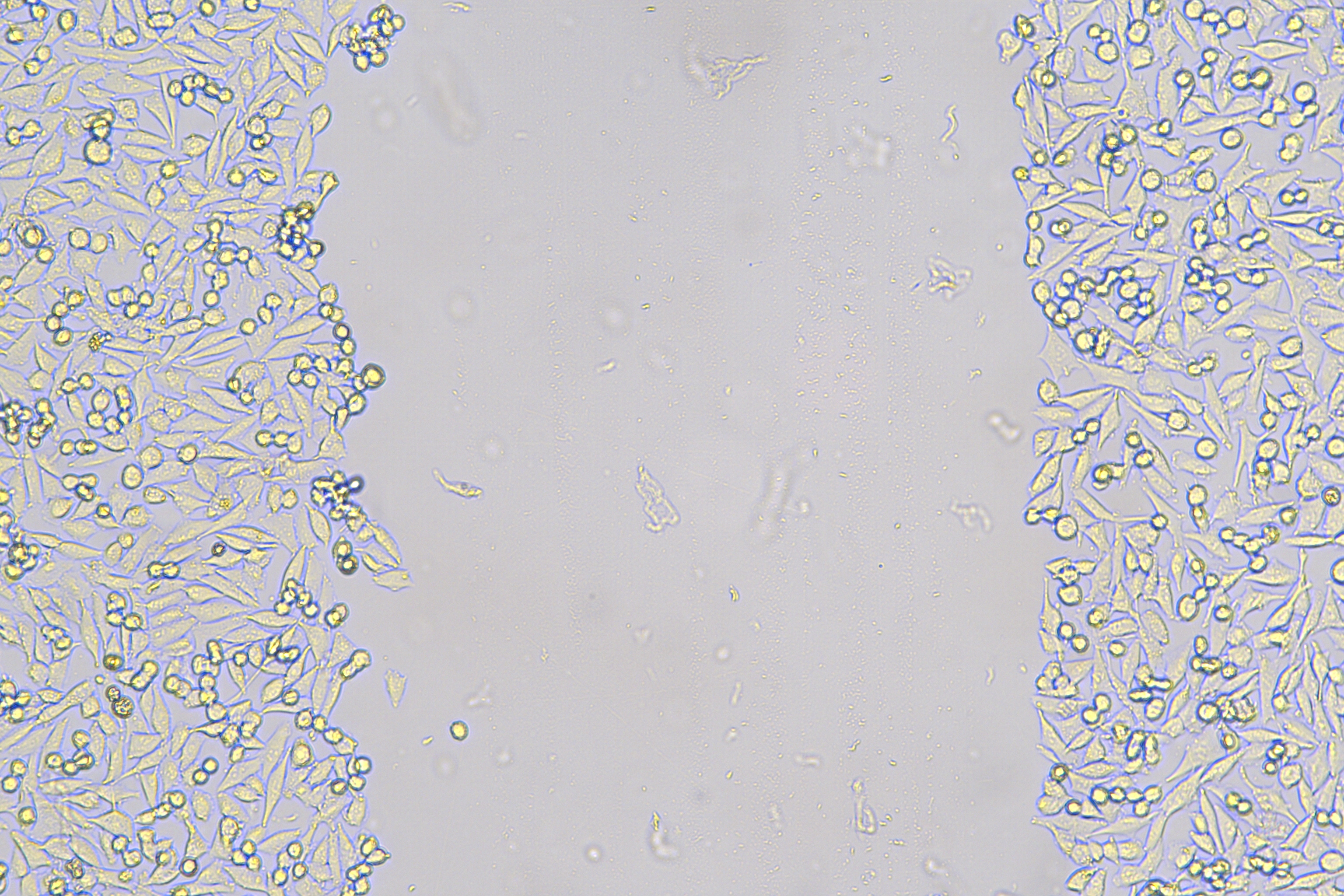

Supplement: Multimedia component 1 [file mmc1.zip › the raw data/Figure 6A/Figure 6A wound healing/0 h/OV-CDCA8.jpg]

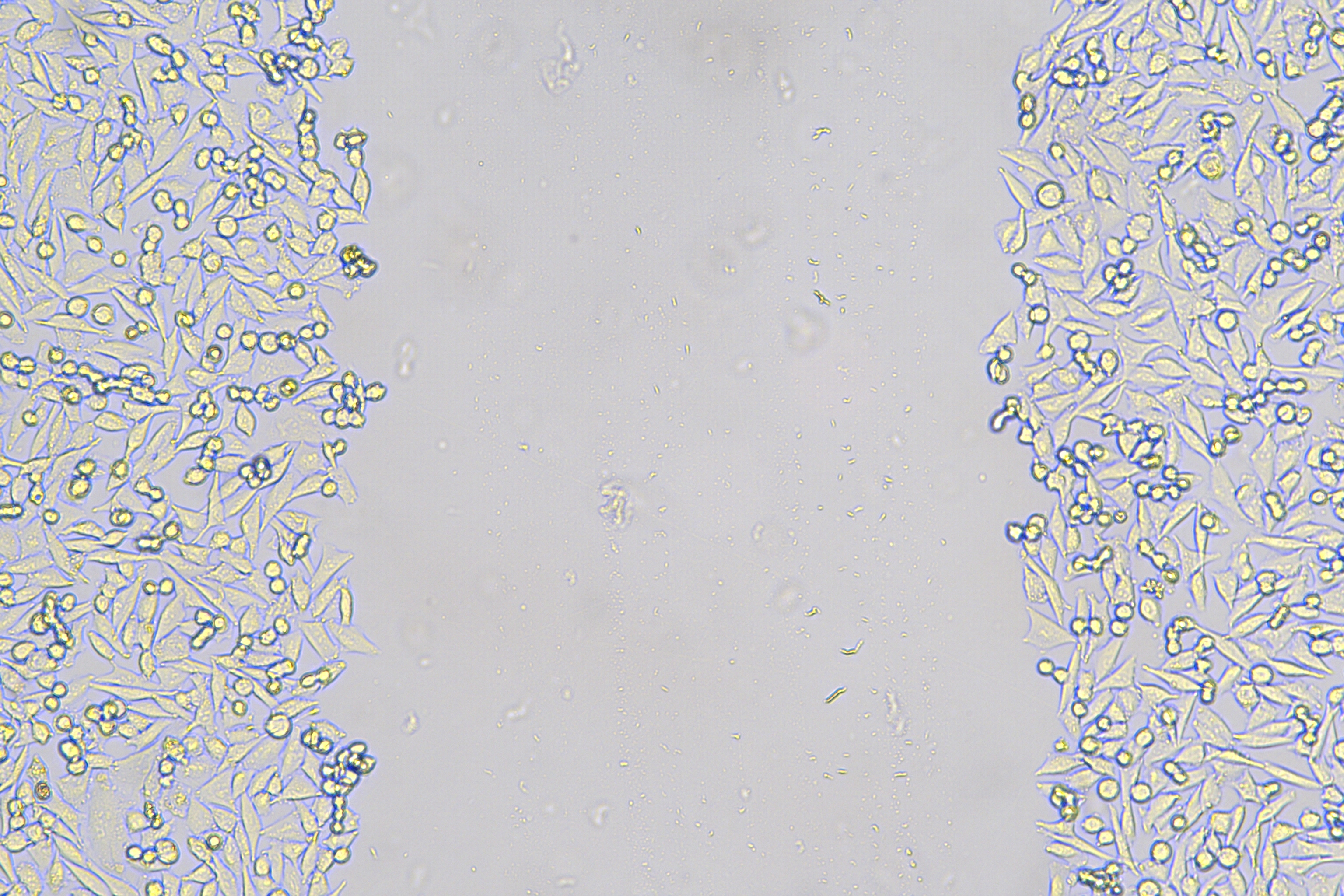

Supplement: Multimedia component 1 [file mmc1.zip › the raw data/Figure 6A/Figure 6A wound healing/0 h/OV-MYBL2.jpg]

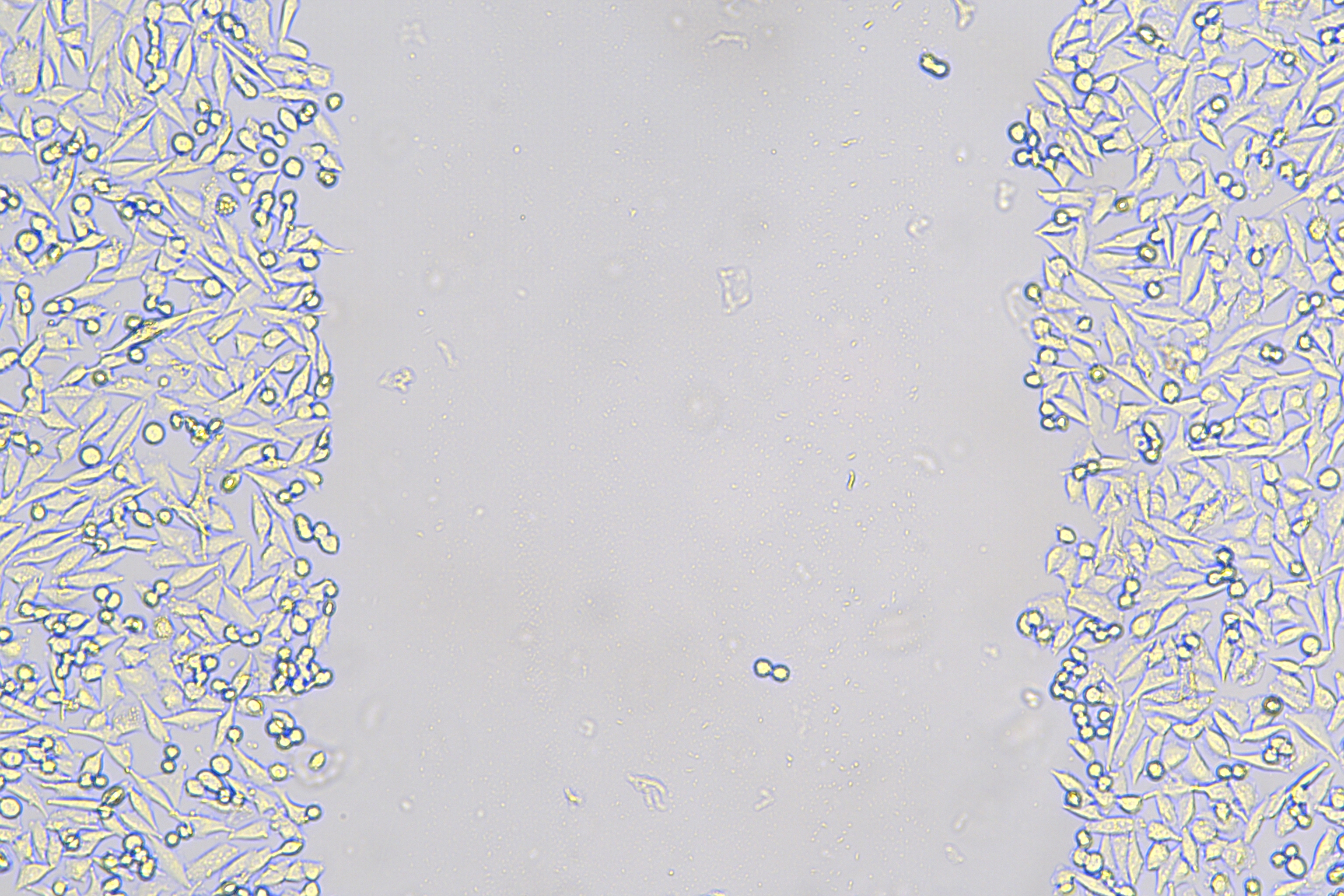

Supplement: Multimedia component 1 [file mmc1.zip › the raw data/Figure 6A/Figure 6A wound healing/0 h/OV-MYBL2+CDCA8.jpg]

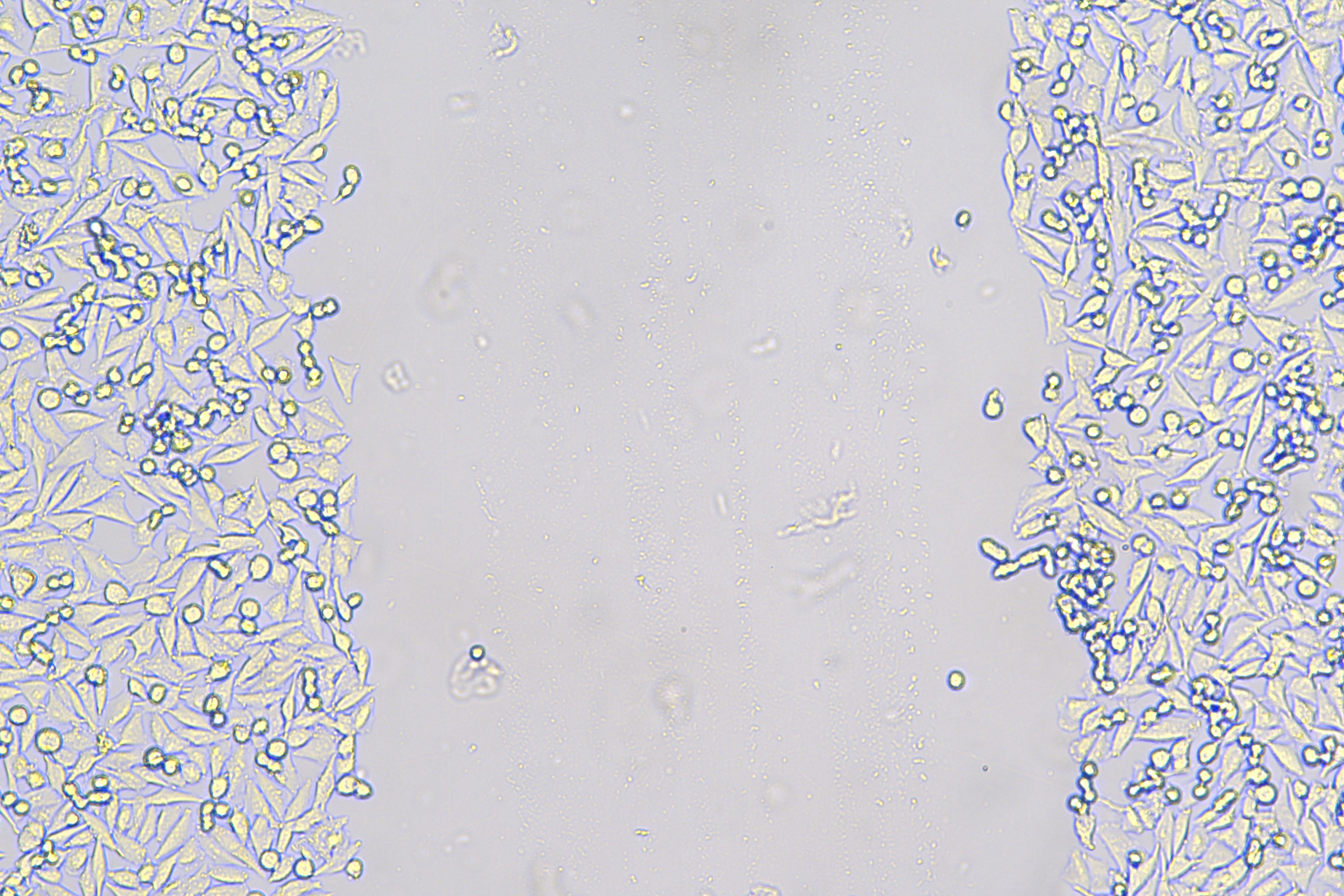

Supplement: Multimedia component 1 [file mmc1.zip › the raw data/Figure 6A/Figure 6A wound healing/0 h/OV-NC.jpg]

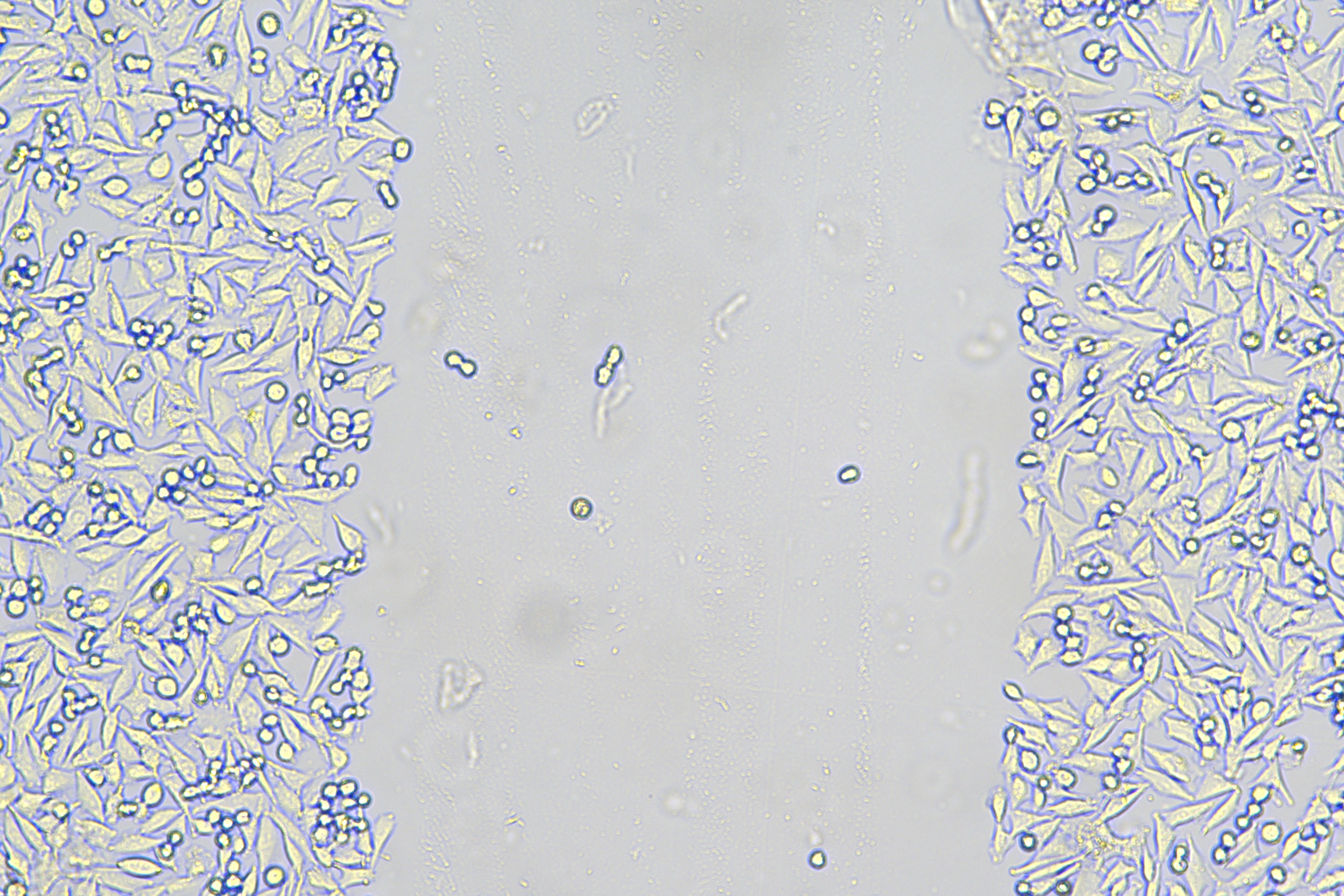

Supplement: Multimedia component 1 [file mmc1.zip › the raw data/Figure 6A/Figure 6A wound healing/0 h/Si-CDCA8.jpg]

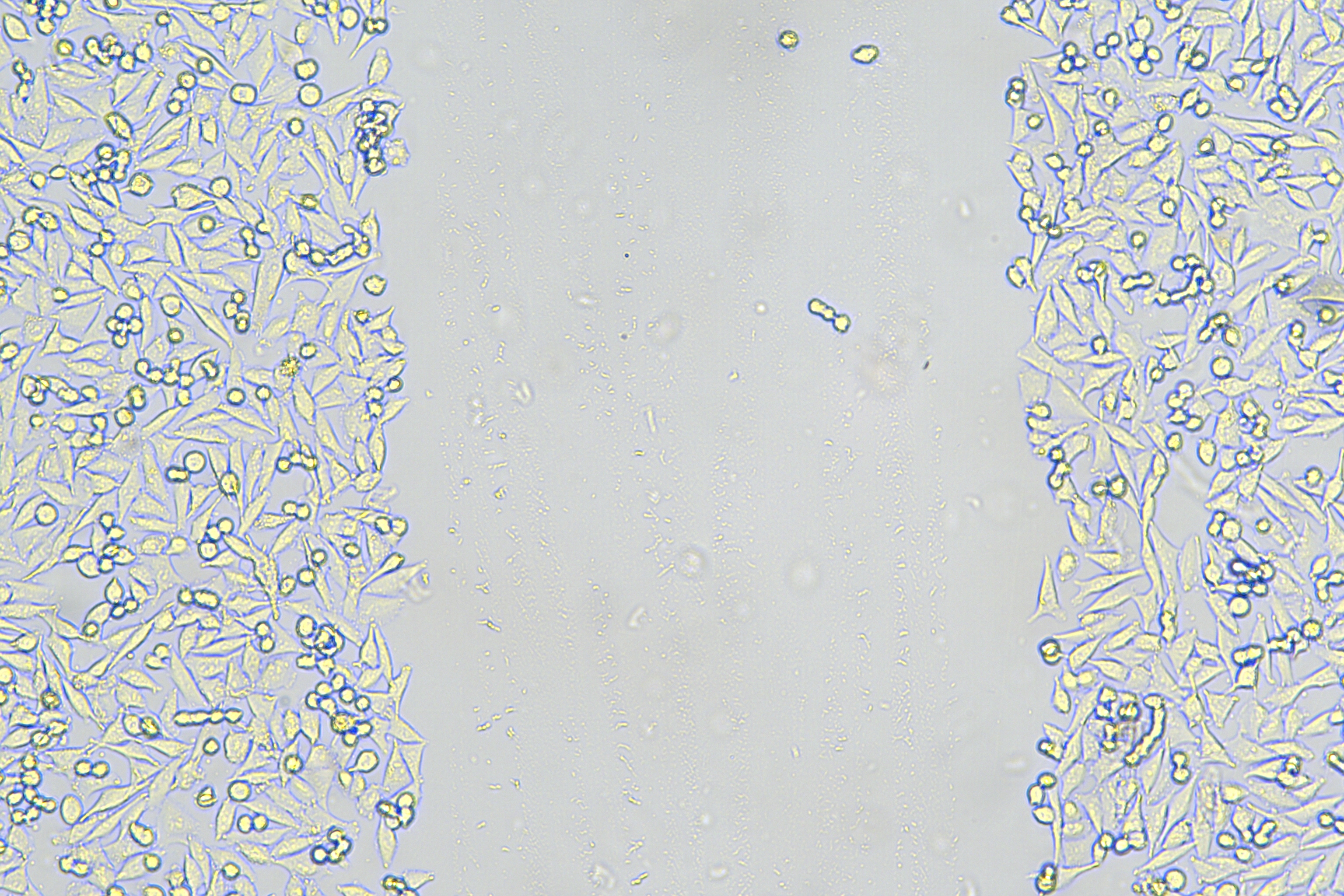

Supplement: Multimedia component 1 [file mmc1.zip › the raw data/Figure 6A/Figure 6A wound healing/0 h/Si-MYBL2.jpg]

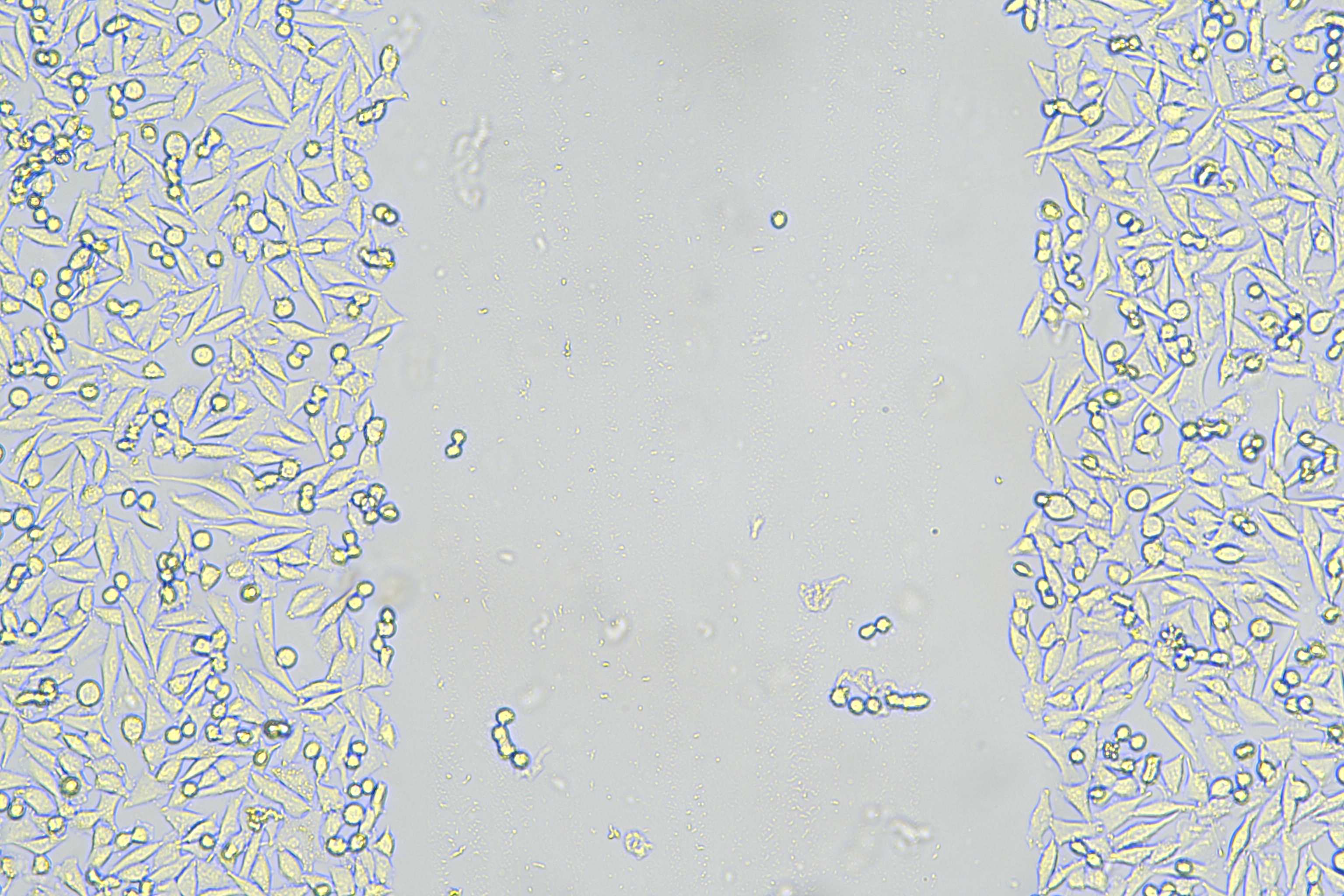

Supplement: Multimedia component 1 [file mmc1.zip › the raw data/Figure 6A/Figure 6A wound healing/0 h/Si-MYBL2+CDCA8.jpg]

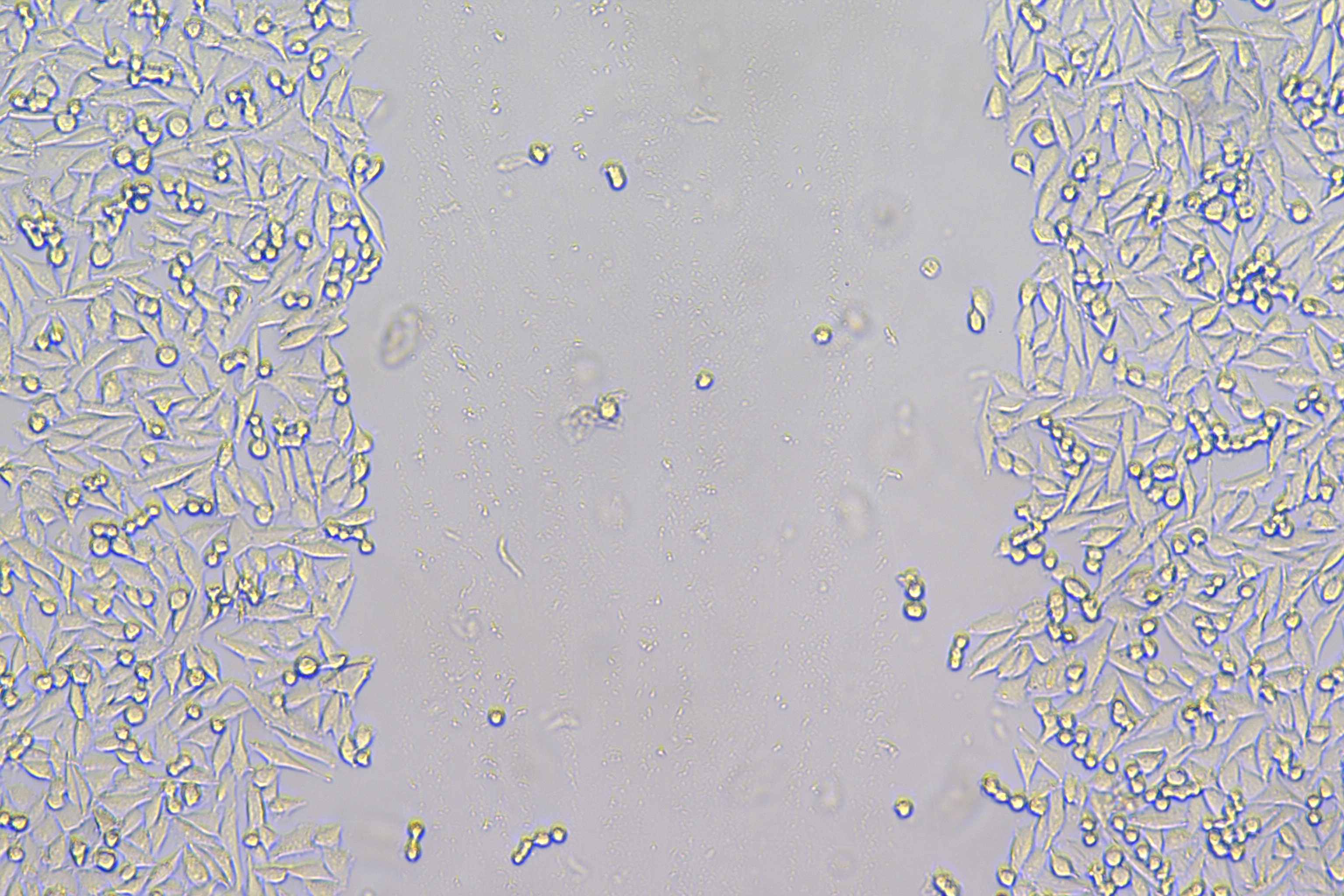

Supplement: Multimedia component 1 [file mmc1.zip › the raw data/Figure 6A/Figure 6A wound healing/0 h/Si-NC.jpg]

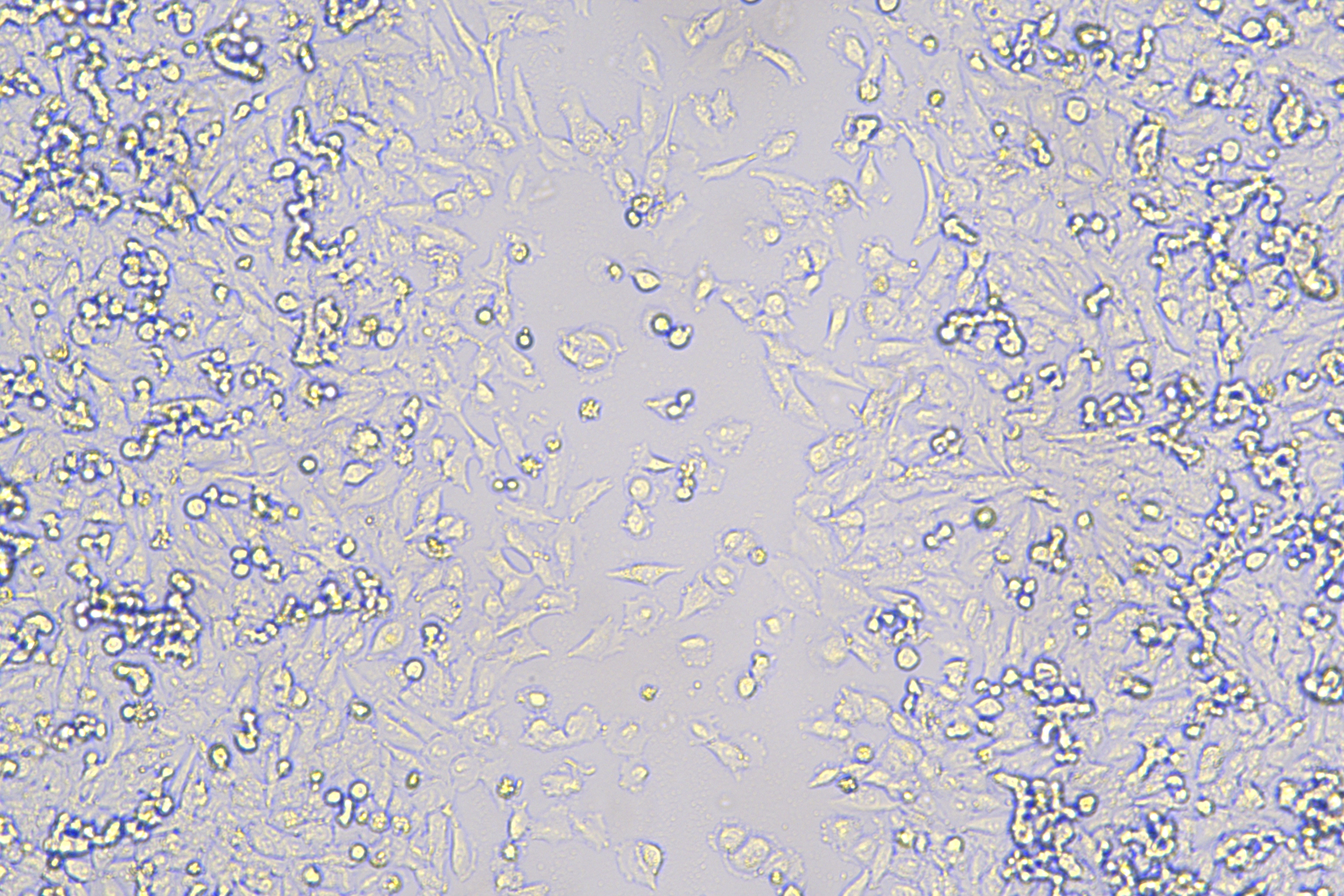

Supplement: Multimedia component 1 [file mmc1.zip › the raw data/Figure 6A/Figure 6A wound healing/24 h/OV-CDCA8.jpg]

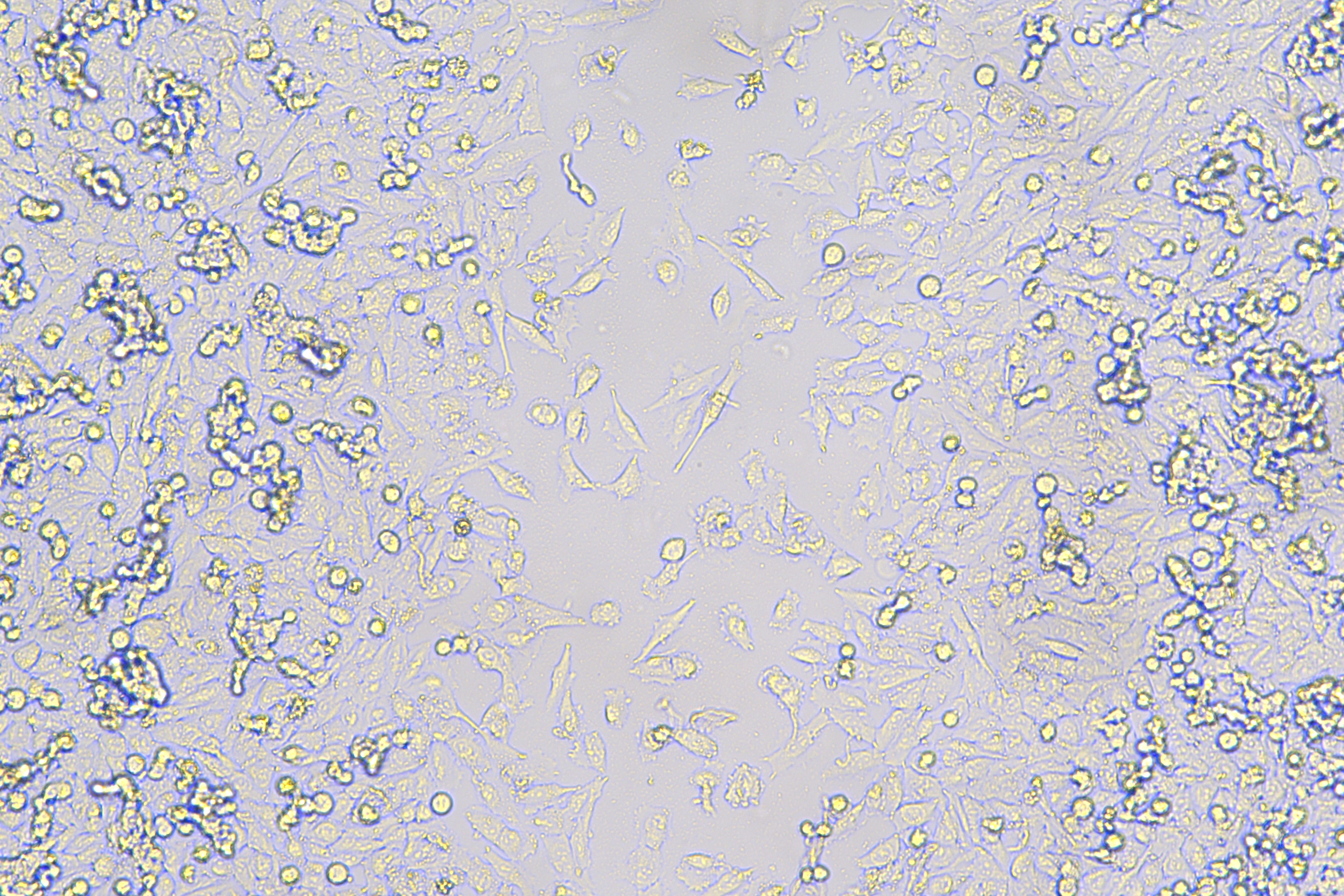

Supplement: Multimedia component 1 [file mmc1.zip › the raw data/Figure 6A/Figure 6A wound healing/24 h/OV-MYBL2.jpg]

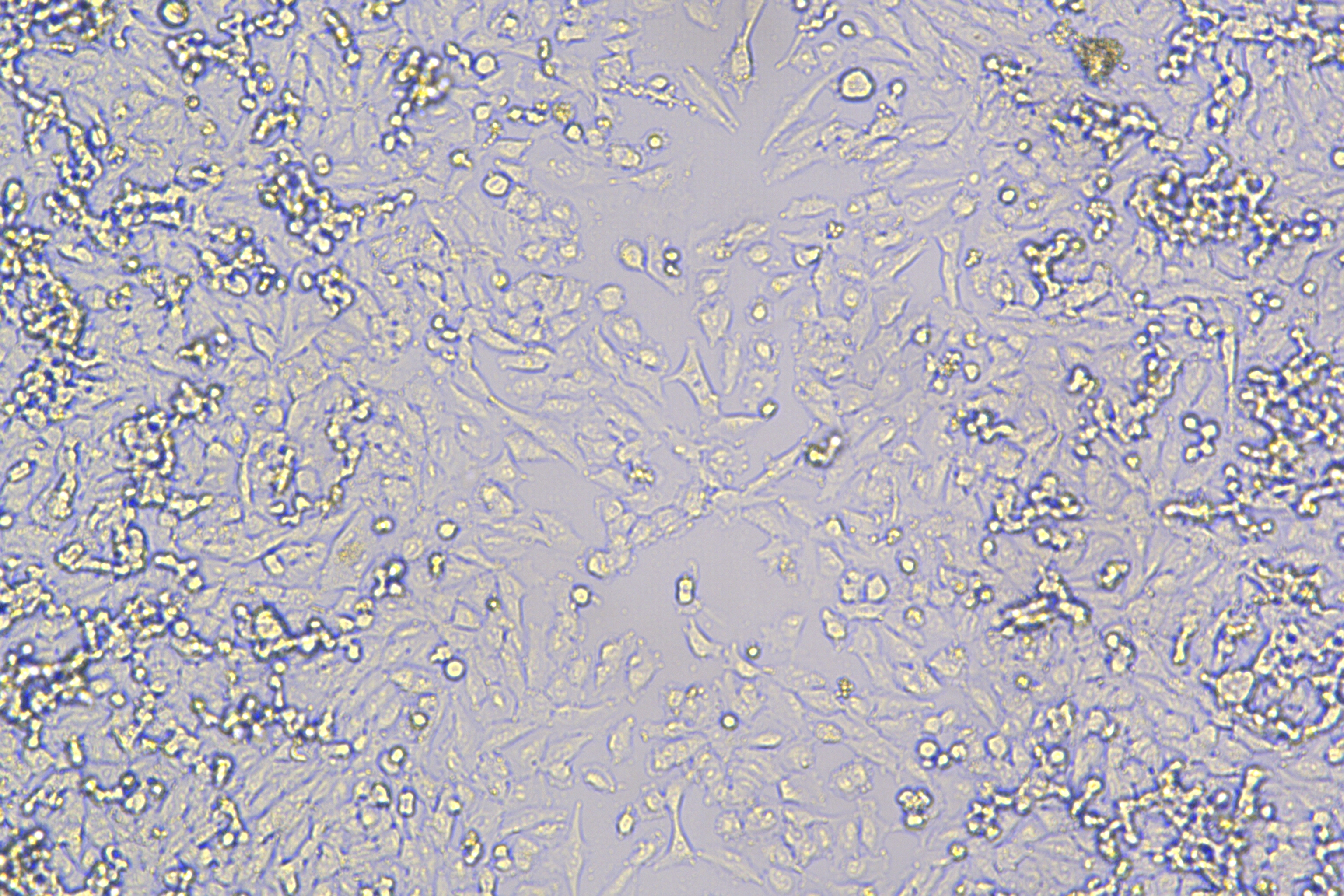

Supplement: Multimedia component 1 [file mmc1.zip › the raw data/Figure 6A/Figure 6A wound healing/24 h/OV-MYBL2+CDCA8.jpg]

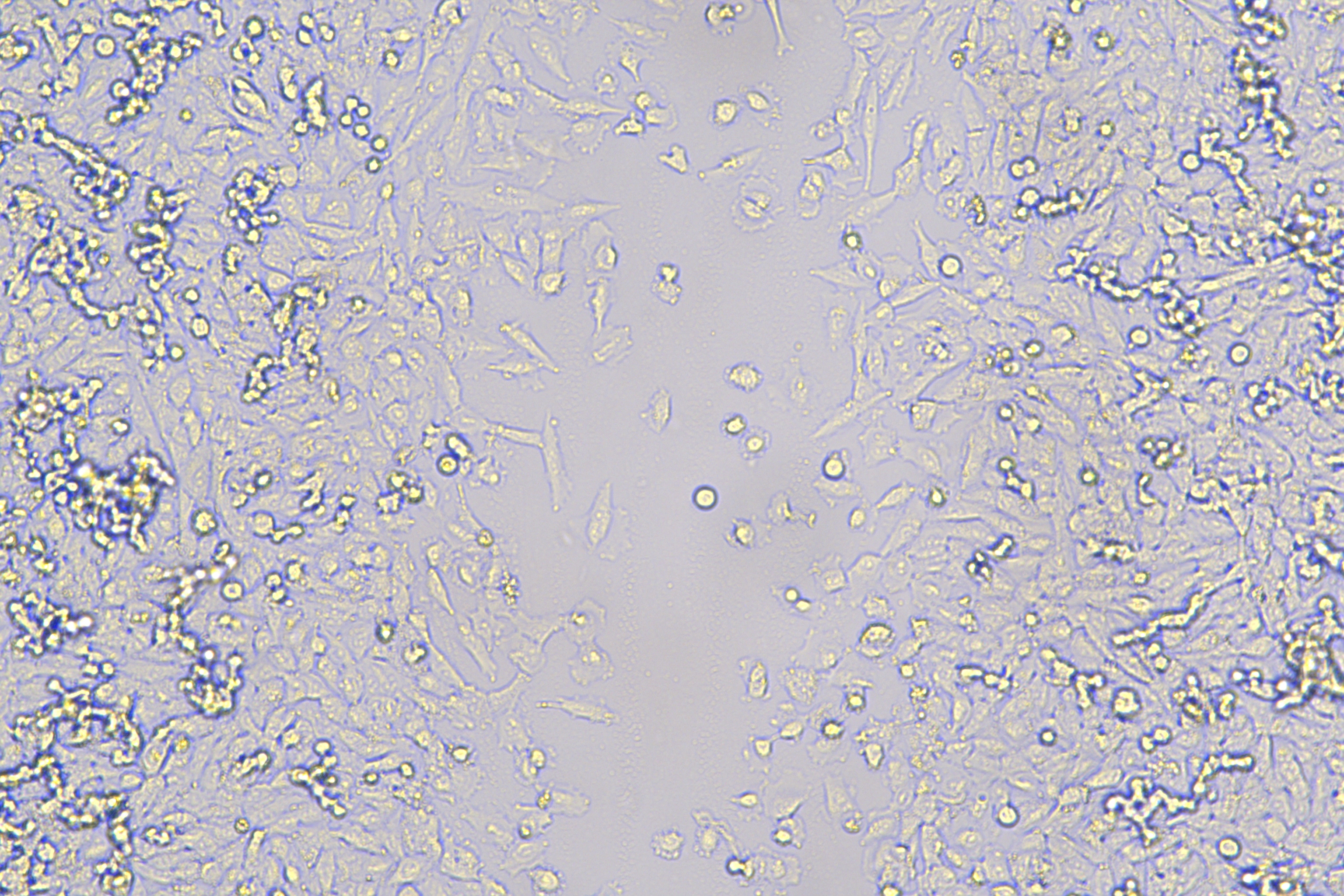

Supplement: Multimedia component 1 [file mmc1.zip › the raw data/Figure 6A/Figure 6A wound healing/24 h/OV-NC.jpg]

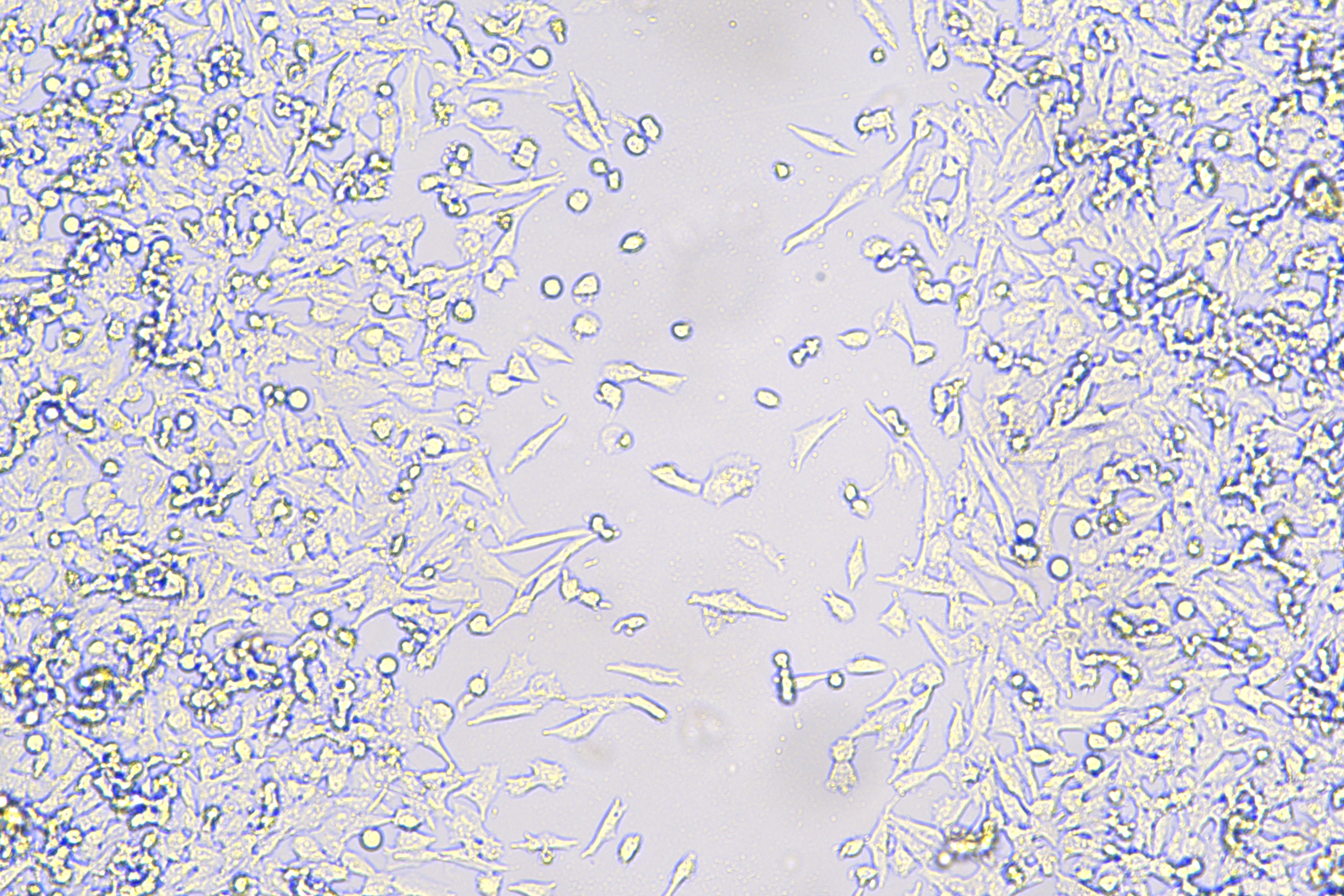

Supplement: Multimedia component 1 [file mmc1.zip › the raw data/Figure 6A/Figure 6A wound healing/24 h/Si-CDCA8.jpg]

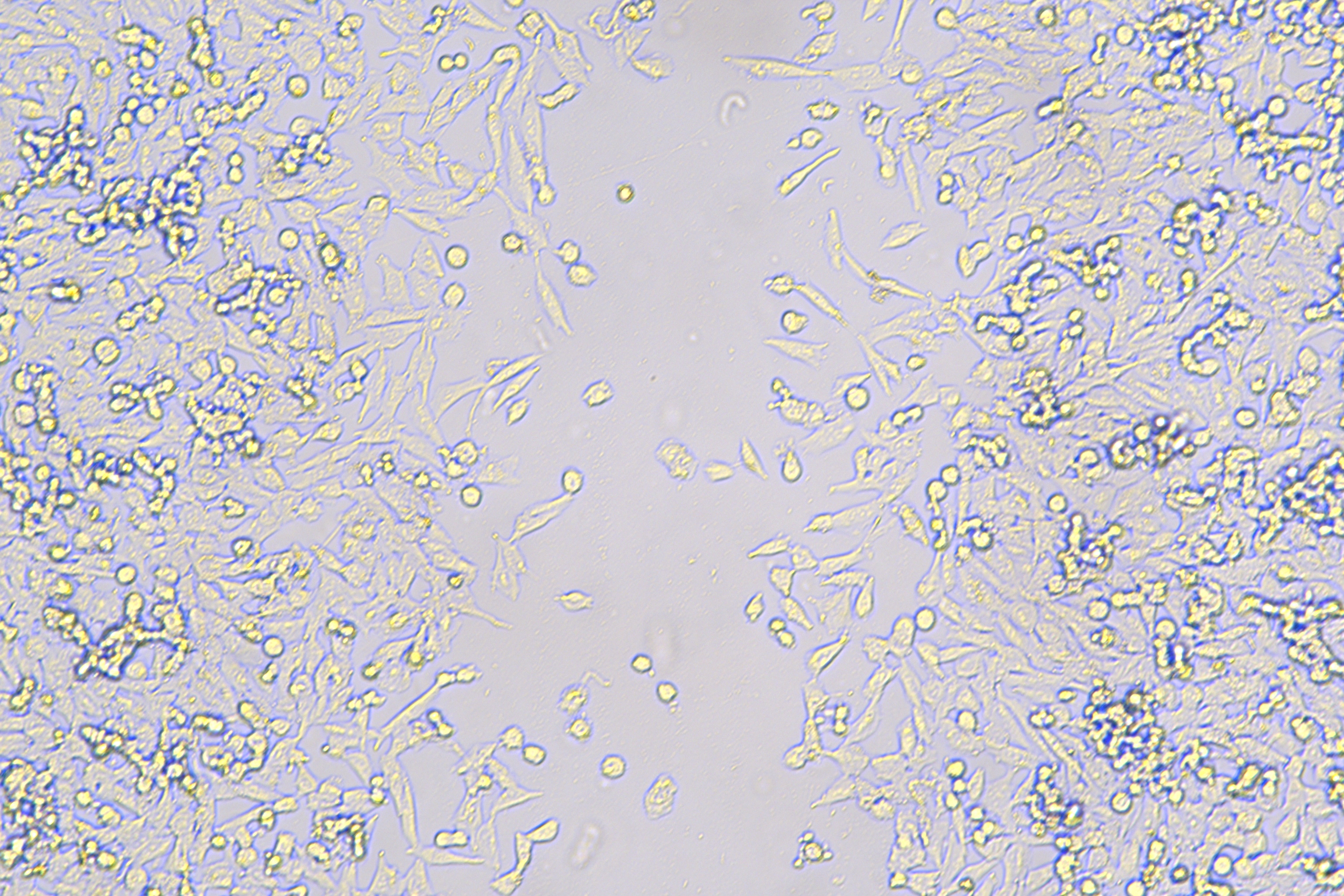

Supplement: Multimedia component 1 [file mmc1.zip › the raw data/Figure 6A/Figure 6A wound healing/24 h/Si-MYBL2.jpg]

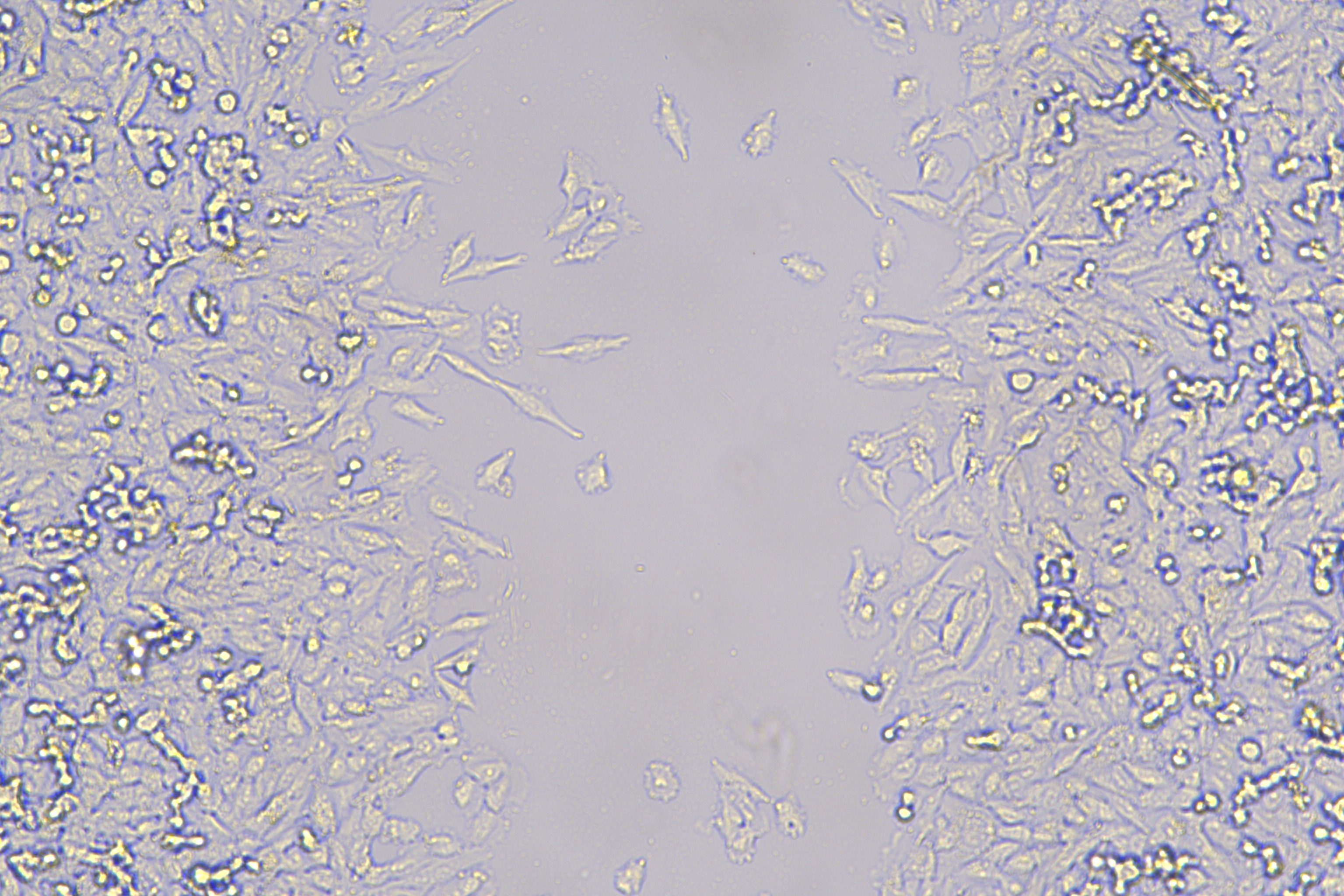

Supplement: Multimedia component 1 [file mmc1.zip › the raw data/Figure 6A/Figure 6A wound healing/24 h/Si-MYBL2+CDCA8.jpg]

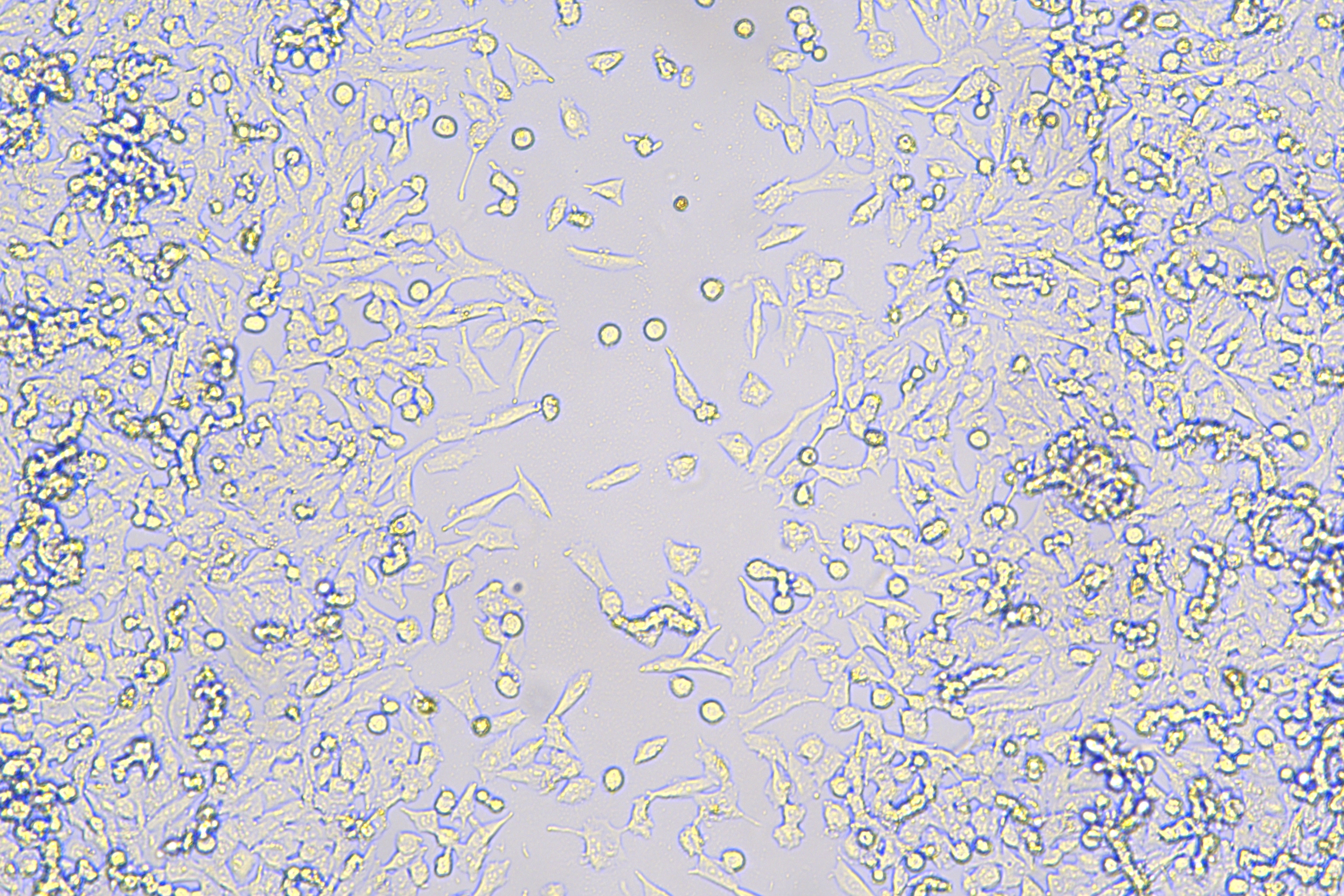

Supplement: Multimedia component 1 [file mmc1.zip › the raw data/Figure 6A/Figure 6A wound healing/24 h/Si-NC.jpg]

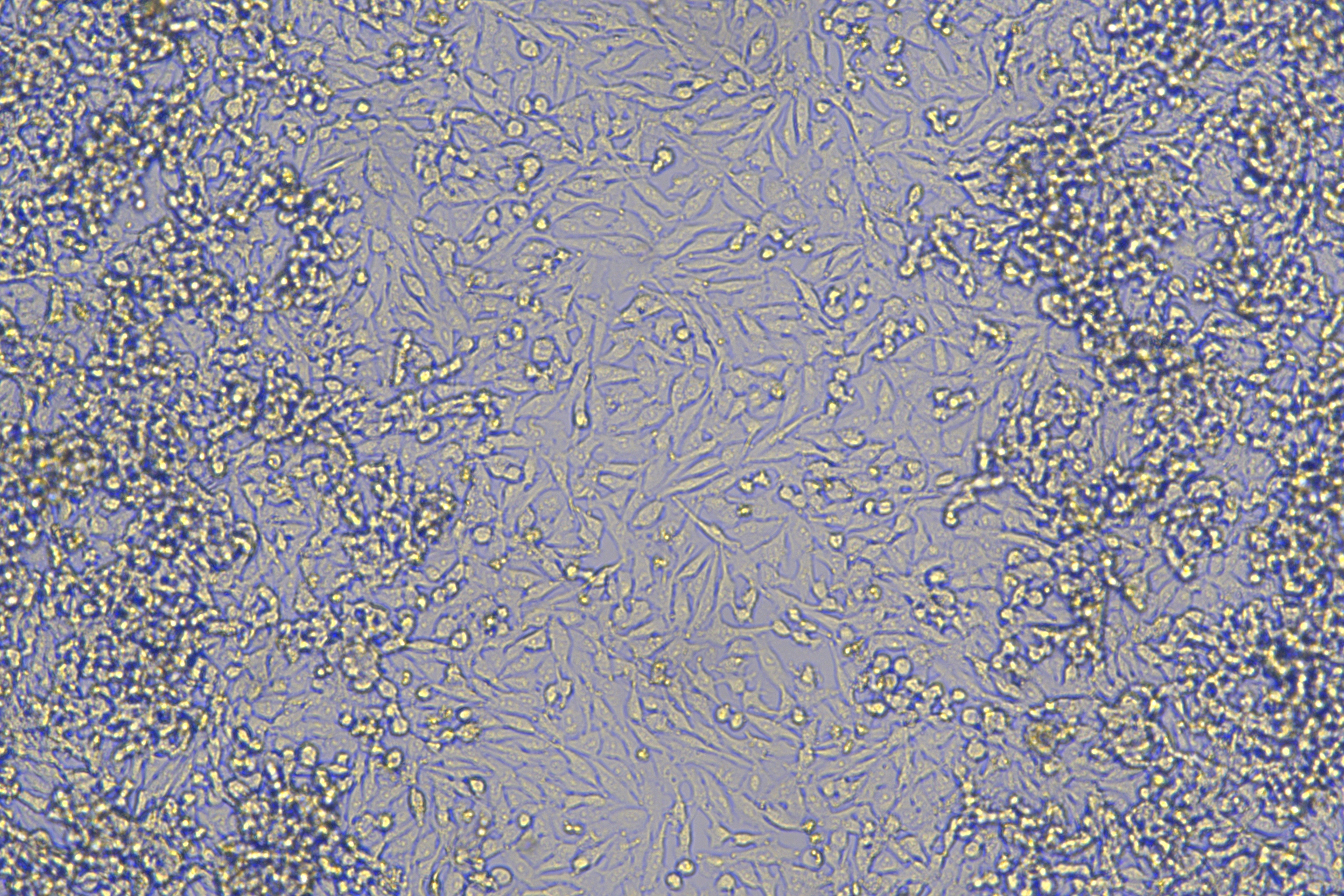

Supplement: Multimedia component 1 [file mmc1.zip › the raw data/Figure 6A/Figure 6A wound healing/48 h/OV-CDCA8.jpg]

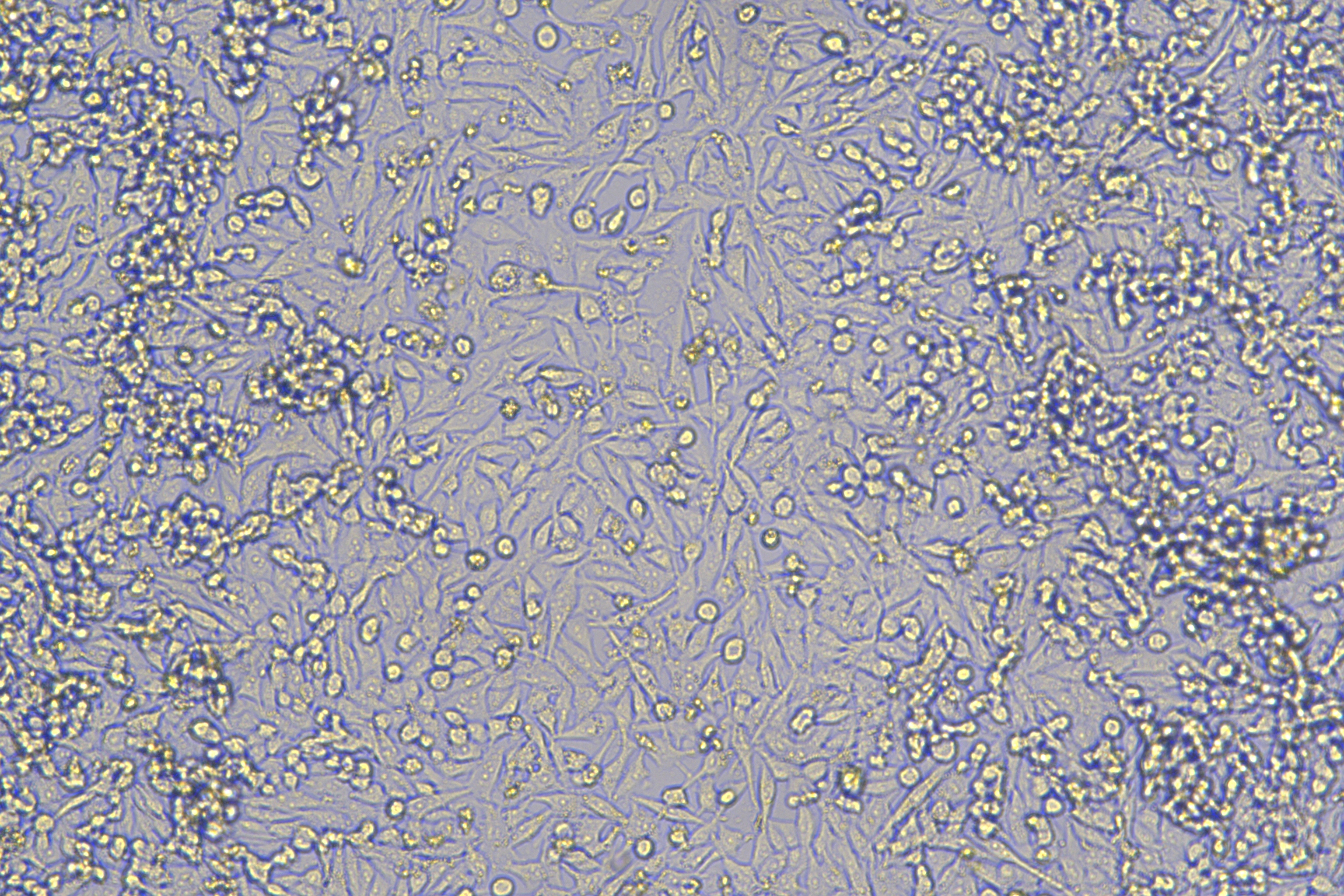

Supplement: Multimedia component 1 [file mmc1.zip › the raw data/Figure 6A/Figure 6A wound healing/48 h/OV-MYBL2.jpg]

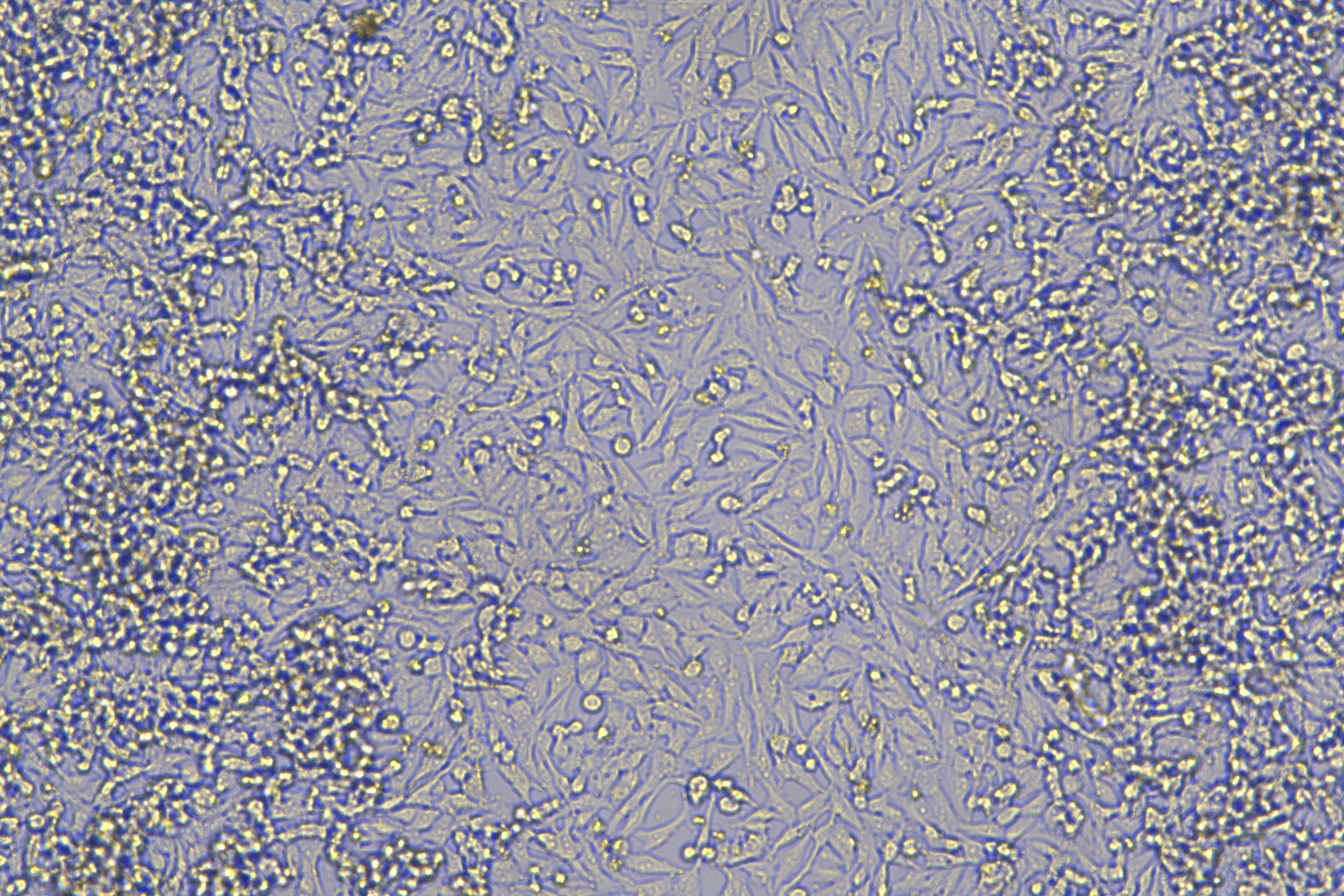

Supplement: Multimedia component 1 [file mmc1.zip › the raw data/Figure 6A/Figure 6A wound healing/48 h/OV-MYBL2+CDCA8.jpg]

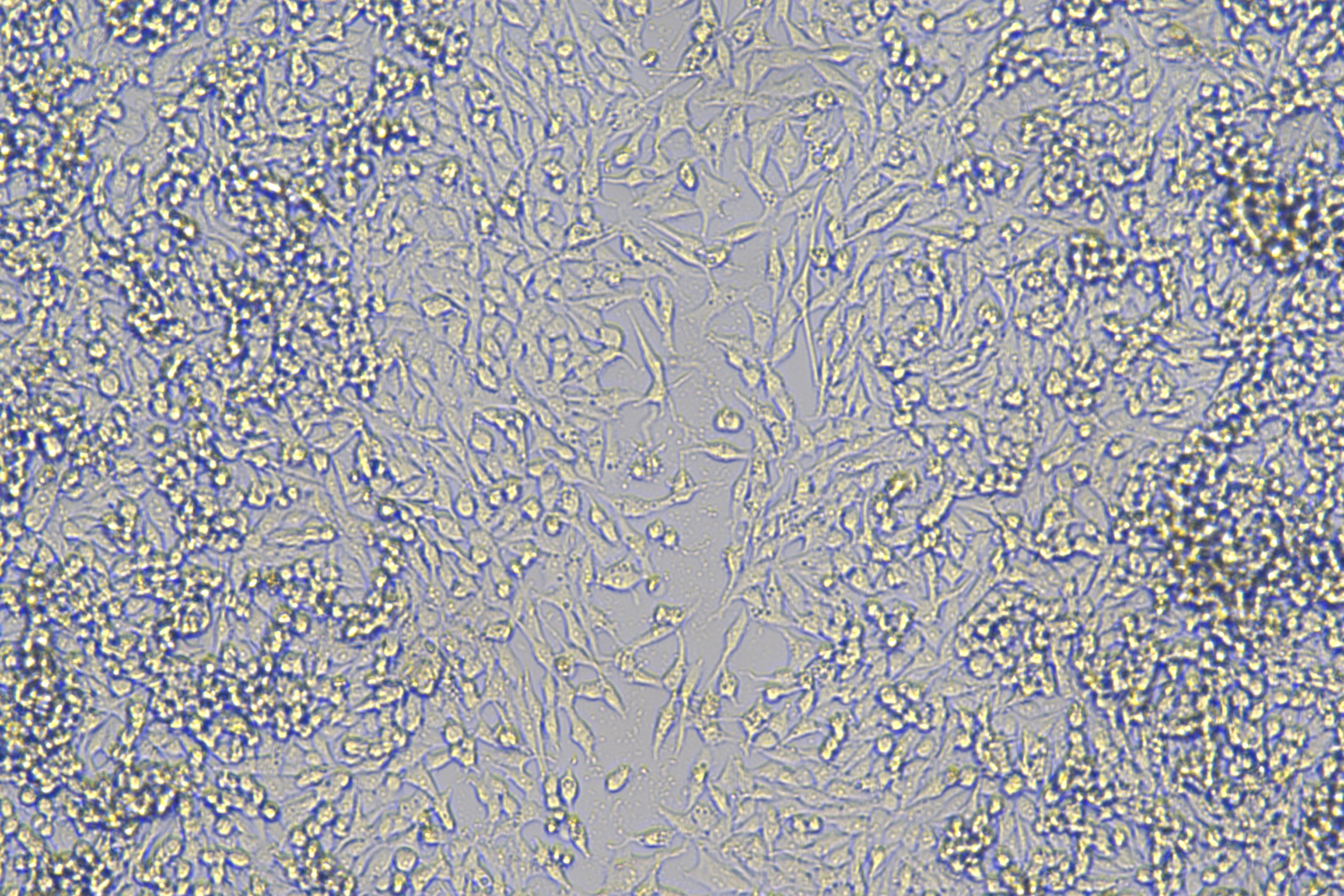

Supplement: Multimedia component 1 [file mmc1.zip › the raw data/Figure 6A/Figure 6A wound healing/48 h/OV-NC.jpg]

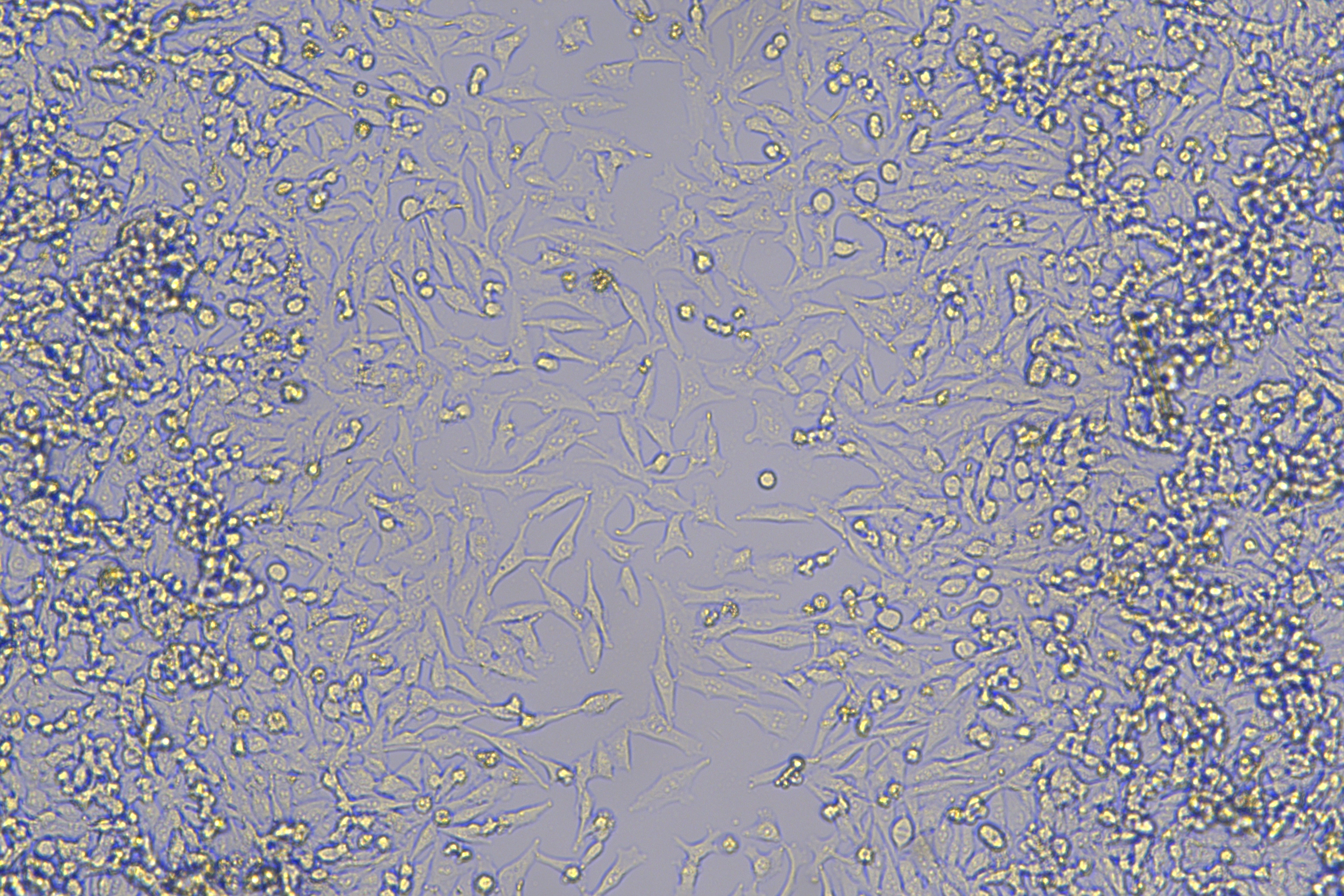

Supplement: Multimedia component 1 [file mmc1.zip › the raw data/Figure 6A/Figure 6A wound healing/48 h/Si-CDCA8.jpg]

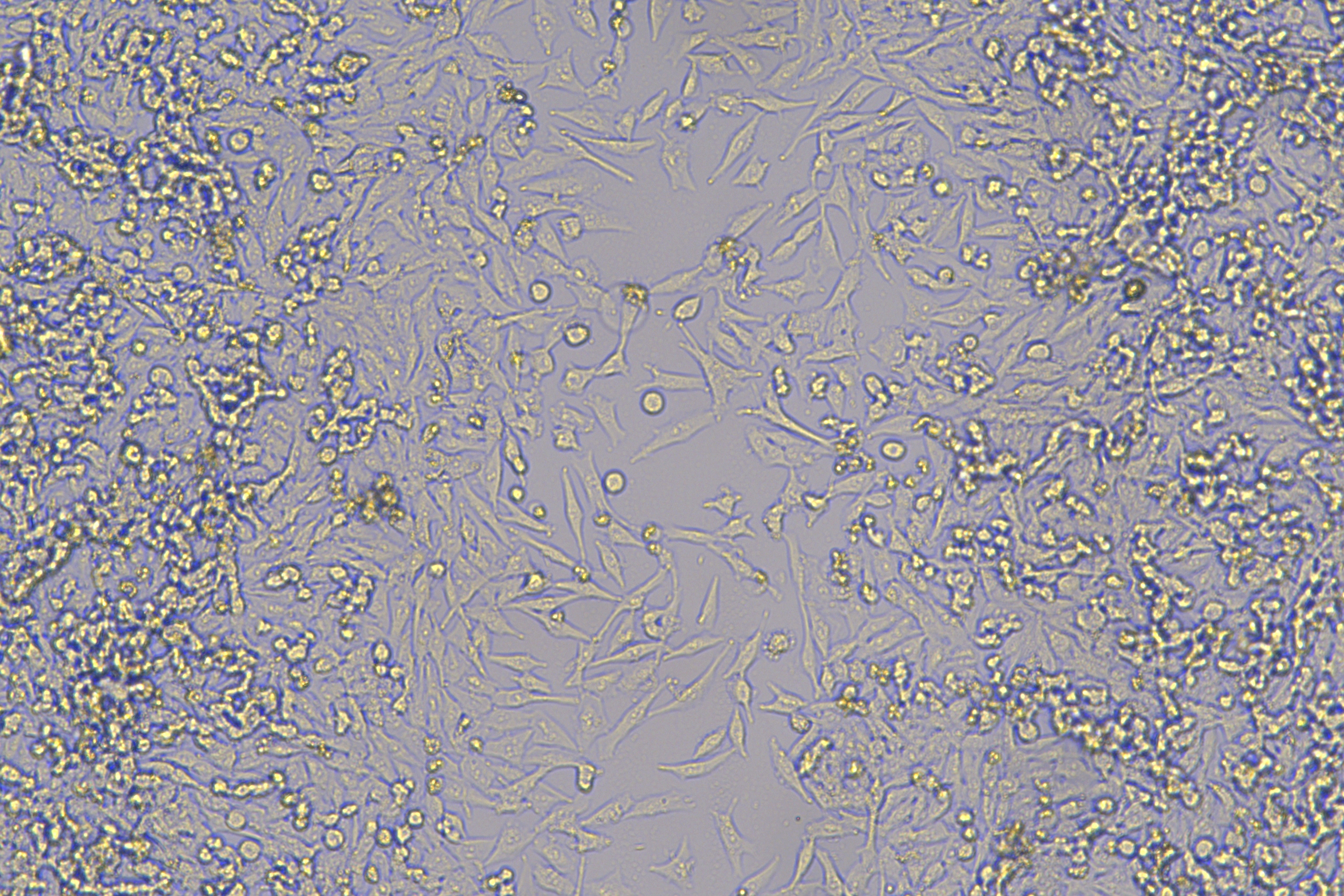

Supplement: Multimedia component 1 [file mmc1.zip › the raw data/Figure 6A/Figure 6A wound healing/48 h/Si-MYBL2.jpg]

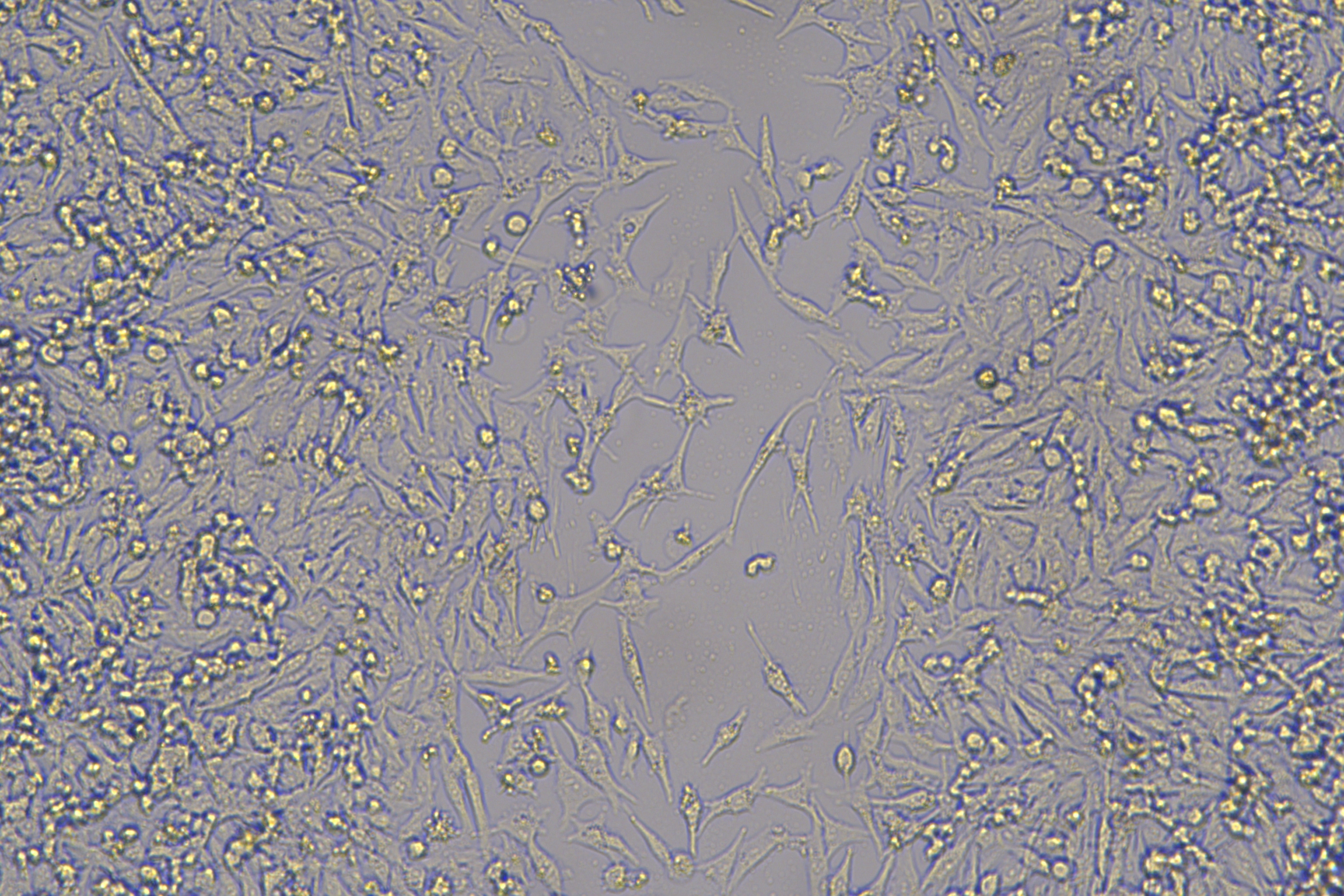

Supplement: Multimedia component 1 [file mmc1.zip › the raw data/Figure 6A/Figure 6A wound healing/48 h/Si-MYBL2+CDCA8.jpg]

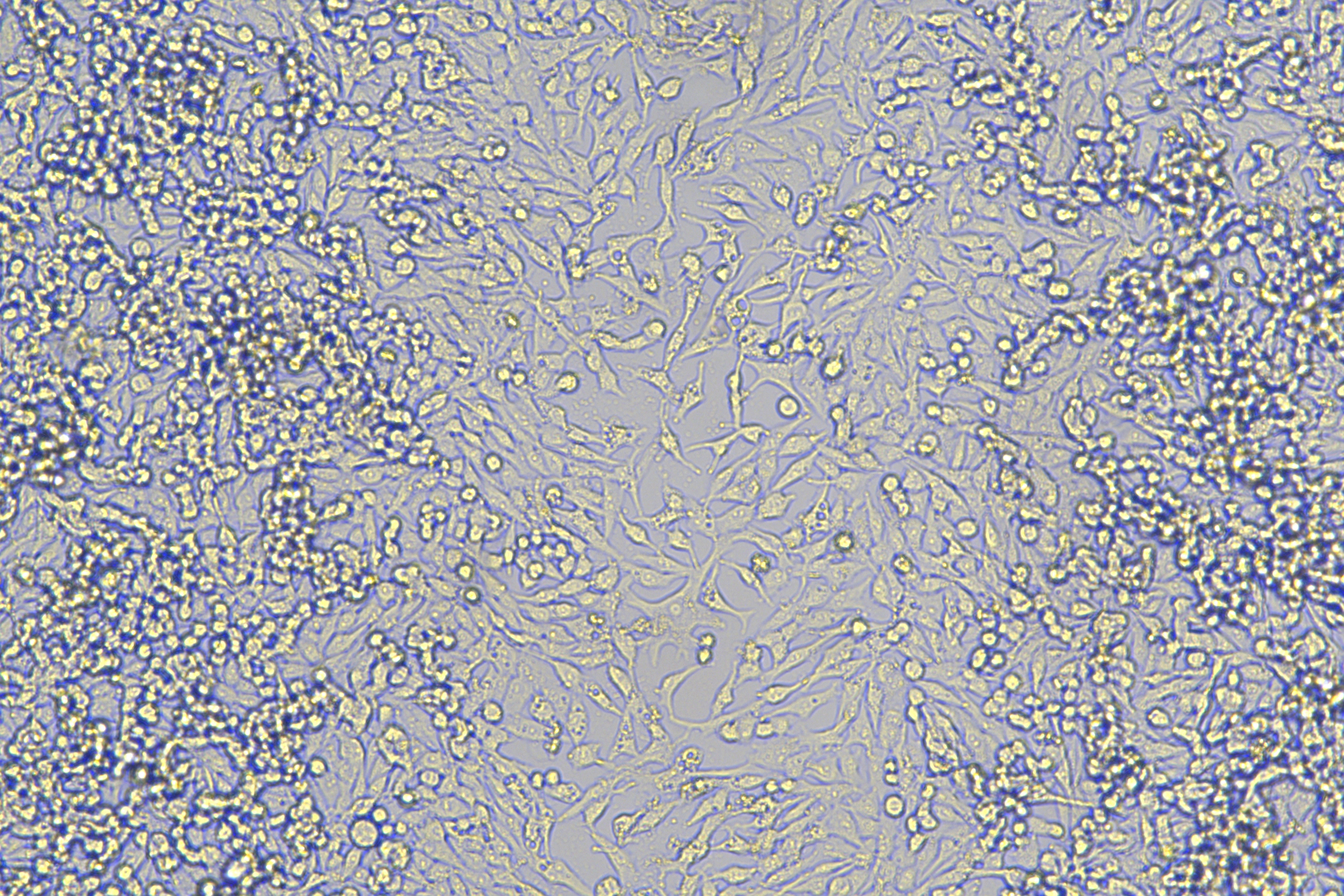

Supplement: Multimedia component 1 [file mmc1.zip › the raw data/Figure 6A/Figure 6A wound healing/48 h/Si-NC.jpg]
